# Interpretable machine learning for precision cognitive aging

**DOI:** 10.3389/fncom.2025.1560064

**Published:** 2025-05-16

**Authors:** Abdoul Jalil Djiberou Mahamadou, Emma A. Rodrigues, Vasily Vakorin, Violaine Antoine, Sylvain Moreno

**Affiliations:** ^1^Stanford Center for Biomedical Ethics, Stanford University, Stanford, CA, United States; ^2^School of Interactive Arts and Technology, Simon Fraser University, Surrey, BC, Canada; ^3^Department of Biomedical Physiology and Kinesiology, Simon Fraser University, Burnaby, BC, Canada; ^4^Royal Columbian Hospital, Fraser Health Authority, New Westminster, BC, Canada; ^5^CNRS ENSMSE LIMOS, Clermont Auvergne University, Clermont-Ferrand, France; ^6^Circle Innovation, Simon Fraser University, Surrey, BC, Canada

**Keywords:** explainable AI, explainable boosting machine, personalized cognitive aging, healthy aging, subtyping

## Abstract

**Introduction:**

Machine performance has surpassed human capabilities in various tasks, yet the opacity of complex models limits their adoption in critical fields such as healthcare. Explainable AI (XAI) has emerged to address this by enhancing transparency and trust in AI decision-making. However, a persistent gap exists between interpretability and performance, as black-box models, such as deep neural networks, often outperform white-box models, such as regression-based approaches. To bridge this gap, the Explainable Boosting Machine (EBM), a class of generalized additive models has been introduced, combining the strengths of interpretable and high-performing models. EBM may be particularly well-suited for cognitive health research, where traditional models struggle to capture nonlinear effects in cognitive aging and account for inter- and intra-individual variability.

**Methods:**

This cross-sectional study applies EBM to investigate the relationship between demographic, environmental, and lifestyle factors, and cognitive performance in a sample of 3,482 healthy older adults. The EBM’s performance is compared against Logistic Regression, Support Vector Machines, Random Forests, Multilayer Perceptron, and Extreme Gradient Boosting, evaluating predictive accuracy and interpretability.

**Results:**

The findings reveal that EBM provides valuable insights into cognitive aging, surpassing traditional models while maintaining competitive accuracy with more complex machine learning approaches. Notably, EBM highlights variations in how lifestyle activities impact cognitive performance, particularly differences between engaging in and refraining from specific activities, challenging regression-based assumptions. Moreover, our results show that the effects of lifestyle factors are heterogeneous across cognitive groups, with some individuals demonstrating significant cognitive changes while others remain resilient to these influences.

**Discussion:**

These findings highlight EBM’s potential in cognitive aging research, offering both interpretability and accuracy to inform personalized strategies for mitigating cognitive decline. By bridging the gap between explainability and performance, this study advances the use of XAI in healthcare and cognitive aging research.

## Introduction

Artificial Intelligence (AI) is transforming scientific research, driving advancements in medical diagnosis (Aggarwal et al., 2023; Kumar et al., 2023), protein structure prediction (Jumper et al., 2021), drug discovery and development (Paul et al., 2021), strategic gameplay (Silver et al., 2017) and natural language processing (Bommasani et al., 2022). In aging research, AI has the potential to promote cognitive health and support independent living by identifying key determinants of cognitive aging (Pollack, 2005). With the global population of adults over 65 projected to surpass 1.6 billion by 2050 (Jumper et al., 2021), understanding modifiable factors that support healthy cognitive aging is an urgent priority. AI-powered approaches enable real-time monitoring of vital signs, cognition and daily activities (Czaja and Ceruso, 2022), providing new insights into aging processes. However, despite its potential, a major challenge remains as many AI models lack interpretability, limiting their scientific and practical application in cognitive aging research (Martin et al., 2023). The black-box nature of many high-performing models results in unexplainable, unjustifiable, and unaccountable decision-making processes. This is a significant limitation for healthcare and cognitive science, where transparency is essential (Hassija et al., 2023).

To address these challenges, Explainable AI (XAI) has emerged as a key approach for improving the interpretability, auditability, and trustworthiness of AI models (Gilpin et al., 2018; Hassija et al., 2023; Molnar, 2020). While some authors interchangeably use the terms “interpretable” and “explainable,” distinctions have been made by others (Marcinkevičs and Vogt, 2023). Interpretable models, or white-box models, are inherently understandable, whereas explainable models require additional techniques to clarify their decision-making processes (Ribeiro et al., 2016). XAI methods can provide global explanations, which describe overall model behavior, and local explanations, which focus on individual predictions. These techniques range from model-specific (e.g., regression-based, tree-based, neural networks) to model-agnostic approaches that can be applied across different AI frameworks (Molnar, 2020). Beyond model interpretability, XAI techniques include data explainability (e.g., explainable feature engineering), post-hoc explainability, and the assessment of explanations, including trust and transparency (Ali et al., 2023). While these advancements have improved AI adoption in scientific research, a critical question remains: Can inherently interpretable XAI models provide predictive accuracy comparable to black-box models while offering meaningful insights into cognitive aging?

An emerging approach for addressing this challenge involves generalized additive models (GAMs) (Hastie, 1992) combined with shallow machine learning models (Lengerich et al., 2023). Among these, Explainable Boosting Machine (EBM) (Nori et al., 2019; Zhang et al., 2019), has gained attention for its ability to balance transparency and predictive accuracy. EBM employs bagging and boosting decision trees (Friedman, 2002) to model complex interactions, including nonlinear relationships between predictors. Unlike traditional additive models, such as regression-based models, which associate a single weight to each variable, EBM assigns weights to variable bins, enabling more granular and interpretable feature contributions. This is particularly relevant in cognitive aging research, where understanding individual variability in cognitive outcomes remains a major challenge (Patel et al., 2022; Rodrigues and Moreno, 2023).

While cognitive decline is commonly associated with aging (Rodrigues et al., 2022), some individuals maintain stable cognitive performance on various everyday tasks (Zhang et al., 2015; Lee et al., 2010). This variability remains poorly understood, with multiple theories proposed and no clear consensus on the underlying mechanisms (Rodrigues et al., 2024; Rodrigues and Moreno, 2023). Traditional approaches have struggled to capture the complexity of these individual differences, often relying on simplified linear assumptions that fail to reflect the nonlinear interactions between key drivers of cognitive aging. As such, interpretable approaches, such as EBM, offer a promising alternative by enabling the identification of nonlinear relationships between cognitive performance and its predictors while maintaining transparency in decision-making. Given the absence of pharmacological interventions to prevent cognitive decline, research has increasingly focused on identifying modifiable factors that promote good cognitive health. In fact, understanding how lifestyle and environmental factors influence cognitive aging has become a public health research priority (Huang et al., 2020). Emerging evidence suggests that sedentary behavior, poor diet, smoking, and excessive alcohol consumption are associated with accelerated cognitive decline in old age (Wajman et al., 2018). Conversely, engagement in healthy lifestyle choices may preserve cognitive aging (Huang et al., 2020). However, the impact of these factors varies significantly across individuals, leading to inconsistent findings and challenges in identifying robust associations (Lindenberger et al., 2011). This inconsistency may be linked to the limitations of traditional methods, which often rely on mean group comparisons or linear models that fail to fully capture heterogeneous outcomes of cognitive aging (Christie et al., 2017).

Beyond individual variability, cognitive aging research faces additional methodological challenges, including small effect sizes, lack of reproducibility, and reliance on conventional statistical models that impose linearity assumptions (Krivanek et al., 2021). Many studies also suffer from limited sample size, lack of randomized control trials, and focus on univariate analyses, not fully capturing real-life impacts of diverse environmental and lifestyle factors on measures of cognition (Machado et al., 2018; Salthouse, 2000). As an example, a systematic review of 893 papers in clinical psychology found that 92% relied on linear methods and were unclear about approaches used (Ernst and Albers, 2017). In addition, the study of cognitive health in aging has often relied on linear models to assess the effects of age on cognitive outcomes (Ghisletta et al., 2020; Krakovska et al., 2019). While these models are interpretable, they assume a constant rate of cognitive decline across the lifespan, which does not reflect the complex and nonlinear nature of cognitive aging (Chen et al., 2016). Addressing this limitation requires alternative approaches capable of capturing nonlinear interactions while maintaining transparency. Some studies have begun leveraging machine learning techniques, such as Support Vector Machines (SVM) (e.g., Dai et al., 2017; Wu et al., 2020, 2021) and Random Forests (RF) (e.g, Dai et al., 2017; Wu et al., 2021), to model cognitive aging. However, these approaches often function as black box models which are suboptimal for health data where replicability and understanding the model’s decisions is critical (Das and Do, 2023). As a solution, XAI techniques have been explored in pathological research, demonstrating how explainable models can improve transparency in clinical decision-making. For instance, XGB has been successfully applied in the early detection of Parkinson’s disease by integrating statistical analysis with recurrent neural networks to differentiate neurodegenerative conditions, achieving high accuracy while enhancing model interpretability (Cincovic et al., 2024), however, its applicability in healthy aging remains unclear.

To address this gap, we apply EBM to investigate how demographic, environmental and modifiable lifestyle factors are associated with cognitive outcomes in a large cohort of healthy older adults. In this paper we analyze data from 3,482 healthy older adults from the Health and Retirement Study (HRS) (Ann Arbor, 2020; Sonnega and Weir, 2014), a large-scale longitudinal database. We examine the relationship between age, education, daily lifestyle activities and socioeconomic status in cognitive health, leveraging EBM as an interpretable machine learning approach. Further, we compare EBM to Logistic Regression (LR), Support Vector Machines (SVM), Random Forests (RF), Multilayer Perceptron (MLP), and Extreme Gradient Boosting (XGB), to assess whether an interpretable model can achieve predictive accuracy comparable to black-box models while providing greater transparency. To further investigate individual variability in cognitive aging, we stratify participants into cognitive subgroups based on cognitive performance measures. This allows us to examine whether the influence of the selected factors on cognitive performance differs across cognitive groups. To enhance reproducibility, we provided the open-source code and supplementary results in a GitHub repository.^1^

Our hypothesis are fourfold: H_1_ - The EBM model will achieve predictive performance comparable to the state-of-the-art machine learning models (RF XGB, and MLP) while maintaining interpretability; H_2_ - The impact of engaging versus not engaging in specific lifestyle activities will differ significantly across cognitive groups, with measurable variations in magnitude; H_3_ - Engagement in negative health behaviors (e.g., smoking, excessive alcohol consumption, housing instability) will have a stronger detrimental impact on cognitive performance than the protective effects of avoiding these behaviors; H_4_ – Subgroups within the population will exhibit varying responses to environmental and lifestyle factors, with some individuals showing strong cognitive outcomes (both positive and negative) in response to exposures, while others remain resistant, exhibiting minimal cognitive variation across different environmental exposures. By bridging the gap between advanced AI capabilities and the need for interpretability in cognitive aging research, this study investigates how transparent machine learning models can lead to meaningful insights into healthy cognitive aging.

## Methodology

### Dataset

The HRS (Ann Arbor, 2020; Sonnega and Weir, 2014) dataset is a longitudinal public survey dataset collected every two years of participants over 50 years old. The collected data includes different components such as income and wealth, health and use of health services, employment, psycho-social, and lifestyle activities. In the present work, we select participants aged over 60 years old from the 2012 (collected from April 2012 to April 2013) and 2016 (collected from April 2016 to April 2018) (Health and Retirement Study, 2019, 2020, p. 1) waves to assess the relationship between cognitive health, background factors, and lifestyle activities. The included waves were selected given that part of the included lifestyle factors were part of a “leave-behind questionnaire,” which is collected every four years. These questions are left with the participants after the core interview and mailed back at a later date. Given this process, attrition rates for this portion of the data are particularly high.

We examined missing data patterns across all variables, with particular attention to items from the leave-behind questionnaire. We observed that participants missing one lifestyle variable were typically missing most or all such items, suggesting a non-random pattern of missingness. Given the central role of these lifestyle variables in our analysis and the extent of missingness (often exceeding 65%), we judged the data to be likely Missing Not at Random (MNAR). Therefore, we opted for a complete-case analysis to ensure the robustness of our findings. The proportion of missing data for each variable is provided in Supplementary Table 7, and the distribution of missingness across individuals is shown in Supplementary Figure 24.

### Participants

In this study, we employed a cross-sectional approach. The initial sample consisted of 31,646 observations. Participants aged 60 years or older were selected, leading to a subsample of 9,165 observations. Given the structure of the leave-behind questionnaire, where missingness in one lifestyle variable was strongly associated with missingness in others, individuals with missing values in any lifestyle variable were excluded, reducing the sample to 3,832 observations. To ensure consistency across waves, we further restricted the sample to individuals present in both the 2012 and 2016 waves, resulting in a final sample of 3,482 observations (mean age = 71.5 years, SD = 7.9; mean years of education = 12.9, SD = 2.8). The final sample was balanced with respect to the proportion of male (49.4%) and female (50.6%) participants.

### Cognition

The dependent variable was identified based on available literature. Memory has been widely recognized as a cognitive function that typically declines with age, making it a sensitive indicator of cognitive change over time (Schroeder and Marian, 2012). To maximize the intrinsic variability in the data while minimizing ceiling and floor effects, we selected a composite word recall test (immediate + delayed) as a dependent variable (outcome measure). Cognitive performance scores ranged from 0 to 20, with higher scores indicating better recall ability. The distribution was centered around the middle of the scale (mean cognition score = 9.5, SC = 3.2). Cognitive performance was assessed at each wave.

We stratified and categorized the composite scores into 3 (Cogn_3_), 5 (Cogn_5_), and 9 (Cogn_9_) homogenous cognitive categories with low values (respectively high values) of cognitive categories representing individuals with low (respectively high) cognitive performance. We used Cogn_3_ as the dependent variable in our main experiments and the remaining stratified variables in the verification analyses. As independent variables, we selected two continuous factors: participants’ age (Age) and their number of years of education (Education) from the two waves and 34 daily lifestyle activities and socioeconomic status. A description of each variable is provided in Supplementary Table 1. For simplicity of modeling and interpretations of results, we binarized each lifestyle activity.

### Model selection

A wide range of machine learning techniques for cognitive aging research have been developed and implemented in the literature (Graham et al., 2020). In supervised learning popular techniques include Naïve Bayes, Support Vector Machines (SVM), Logistic Regression (LR), Decision Trees, Random Forests (RF), Ensemble Models, Gradient Boosting, Multilayer Perceptron (MLP), Convolutional Neural Networks, and Long Short-Term Memory (Graham et al., 2020). Similar to Wang et al. (2022), we selected five models - LR, SVM, RF, MLP, Extreme Gradient Boosting (XGB) (Chen and Guestrin, 2016) - to compare against the Explainable Boosting Machine (EBM) in the study of cognitive performance and modifiable lifestyle factors (Nori et al., 2019; Zhang et al., 2019). We selected these models due to their popularity and different levels of performance and interpretability. The EBM model is highlighted in this study given that it addresses the gap between interpretability and performance, common to many modern machine learning methods. White-box models, such as regression-based models, are the first category of models intelligible to human-beings, however their performance is often lower than those of black-box models, such as deep neural networks. The EBM model (Nori et al., 2019; Zhang et al., 2019) was introduced to mitigate this issue. By combining shallow machine learning models, such as bagging and boosting machines (Friedman, 2002), with GAMs (Hastie, 1992), the EBM model can achieve accuracy similar to the state-of-the-art machine learning models while preserving intelligibility (Zschech et al., 2022). In specific, the EBM model associates a weight to each independent variable category in contrast to traditional interpretable models such as the LR which associates a single weight to the entire variable (Bogdanovic et al., 2022). These weights can be used both for global and local interpretations of the model. This granular interpretability could allow more precise profiling of data subjects, as discussed in the following section. Furthermore, this combination allows for the detection of complex patterns such as non-linear associations between the dependent and independent variables (Konstantinov and Utkin, 2021).

Although we did not explicitly model time as a covariate, the EBM model captures temporal dependencies through feature interactions. Specifically, the model learns how features from the 2012 wave interact with features from the 2016 wave. The EBM model partitions the feature space based on these interactions, such that the predictions for the 2016 outcome depend on the features from that wave and on how they relate to the features from the 2012 wave. This ability to model non-linear relationships and interactions between features across different time points allows the EBM to implicitly account for temporal dependencies even without treating time as an explicit covariate. This characteristic is especially valuable when working with repeated-measures data, where the assumption of independence between observations may not hold. Studies such as Ntekouli et al. (2022) and Kobayashi and Alam (2024) have demonstrated that generalized models and tree-based models like EBM can effectively model complex temporal relationships without requiring explicit temporal variables.

Formally, the EBM model is defined by:


$$g(y)=\beta_{0}+\sum_{j}f_{j}(x_{j})+\sum_{i\neq j}f_{\mathrm{ij}}(x_{i},x_{j}),$$

where $x_{j}$ is the $j_{\mathrm{th}}$ independent variable, g a link function and $f_{j}$ a feature mapping function (shape function) associated to the $x_{j}$ variable, $f_{\mathrm{ij}}$ are interaction terms associated to the $i_{\mathrm{th}}$ and $j_{\mathrm{th}}$ variables, y refers to the dependent variable, and $\beta_{0}$ the intercept. Here, we used the logit and boosted trees, respectively, as link and shape functions. The link function, in this case, the logit function, is used to transform the dependent variable y into a space where linear relationships can be assumed between the transformed outcome and the independent variables. The logit link function is particularly used in EBM models for binary outcomes, transforming the probability of the outcomes into log odds. This facilitates the application of linear combinations of predictors and interaction terms. The shape function is represented by boosted trees for each independent variable $x_{j}$ . This approach allows the model to capture non-linear relationships between each independent variable and the dependent variable by aggregating the outcomes of shallow boosted trees. Interaction terms in the EBM model refer to the combined effects of pairs of independent variables $\left(x_{i},x_{j}\right)$ on the dependent variable. These terms allow the model to account for situations where the effect of one variable on the outcome depends on the presence of another variable. These settings correspond to the default Python implementation (InterpretML version 0.2.7) of the model used in this work. This implementation also supports interaction terms only for binary outcomes, therefore we ignored these terms.

At the training phase, independent variables are randomly selected to fit the model with the features contributing the best to the entire model selected each time. The random selection in combination with a small learning rate allows the model to discard feature orders. This approach ensures that the contribution of each variable is considered in the context of the entire model, rather than in isolation. At each iteration, a set of shallow boosted trees is trained on one variable at a time where the outcome of the $j_{\mathrm{th}}$ variable corresponds to the residual of the trained model on the ${\left(j-1\right)}_{\mathrm{th}}$ variable. Each tree is designed to explain different aspects of the variable’s relationship with the response. When aggregated, these trees correspond to a shape function that represents the variable’s contribution to the model’s predictions. This ensemble of shallow trees allows the EBM to capture complex, non-linear relationships and interactions between the variables and the outcome. Through the local scoring procedure (Hastie, 1992), the model associates a weight to each variable bin (yes or no), in contrast to regression-based models where the weights are attributed to the entire variables. The obtained weights are then used to estimate the likelihood of the class membership. In addition, these weights serve as a basis to the model interpretability such as feature importance, local and global explanations.

Overall, the EBM’s strengths are threefold: Firstly, as a glass-box model, it offers competitive accuracy compared to black-box models while providing interpretability (Körner et al., 2024; Whig et al., 2023). This is crucial for ensuring that the model’s predictions are reliable and trustworthy. Secondly, it provides granular explanations by assigning weights to each variable bin, enabling a better understanding of the model’s decision-making process (Konstantinov and Utkin, 2021; Lou et al., 2013). Thirdly, it can model complex interactions and non-linear relationships between features without sacrificing interpretability (Lou et al., 2013). It does so by learning a separate shape function for each feature and, optionally, for pairs of features, allowing it to capture both global and local structures in the data.

### Model performance and interpretability comparison

In contrast to traditional interpretable machine learning models such as the LR which associates a weight to each independent variable, the EBM associates a weight to each independent variable category through a local scoring procedure (Hastie, 1992). This level of interpretability is critical for precision cognitive health interventions as it allows more granular profiling of data subjects. Suppose the weight associated with the covariate Often Use Computer by the LR model is +1.3 for the cognitive category 3. This weight would be interpreted as a unit increase in Often Use Computer will lead to an increase of +1.3 in the likelihood of being in the cognitive category 3. If the reference category of this binary variable is NO, it implies that going from not using a computer to using it, increases the likelihood of being in the highest cognitive category. Implicitly, regression-based models associate the same weight to covariate categories (category 1: performing the activity, category 0: not performing the activity). However, in practice, the magnitude of positive and negative impacts of certain independent variable categories can differ from the state of cognitive performance of data subjects. For instance, smoking or drinking can have a more detrimental impact on individuals with low cognitive health than those with high cognitive health.

In addition to the EBM and LR models which are inherently interpretable, other selected models have some level of interpretability. SVMs provide decision boundaries that can be used to interpret the model. Tree-based models, RF and XGB, provide feature importance for each independent variable, in addition to the visualization of the trees to support the decision-making processes. However, black-box models such as the MLP require *post hoc* explainability to explain the model (Konstantinov and Utkin, 2021; Mahya and Fürnkranz, 2023). To homogenize and facilitate the interpretability of the selected models, two model-agnostic interpretable techniques were used: LIME (Ribeiro et al., 2016) and the Shapley additive explanations (SHAP) (Lundberg and Lee, 2017). We reported the global explanations of the EBM model in the main manuscript to illustrate the effectiveness of the model for precision interventions and compared local explanations of the EBM to the LIME and SHAP explanations of other models in the Supplementary material (see Supplementary Figure 16 for EBM results, Supplementary Figures 7–11 for LIME results and Supplementary Figures 12–15 for SHAP results).

Beyond interpretability comparison, the performance of each model was also compared to test the hypothesis that EBM can achieve similar performance to state-of-the-art shallow machine learning models such as the RF and XGB, and deep neural networks models such as the MLP, while being interpretable. The performance of the models was measured through a five-fold cross-validation and area under the ROC curve (ROC AUC) for each dependent variable (Cogn3, Cogn5, and Cogn9). The performance comparison was supported with statistical tests and post hoc analysis with Kruskal-Wallis analysis of variance and Wilcoxon signed-rank tests. We selected these non-parametric tests as the homoscedasticity criteria to run parametric tests such as ANOVA was not met.

As the EBM model used in this study does not provide statistical significance for either entire variables nor individual bins, significance was estimated at the 5% level of each bin and within each class using Z-tests. The Z-scores were computed from the weights and standard deviations obtained from the EBM model.

## Results

### Lifestyle factors and cognitive health

**Figure 1:**
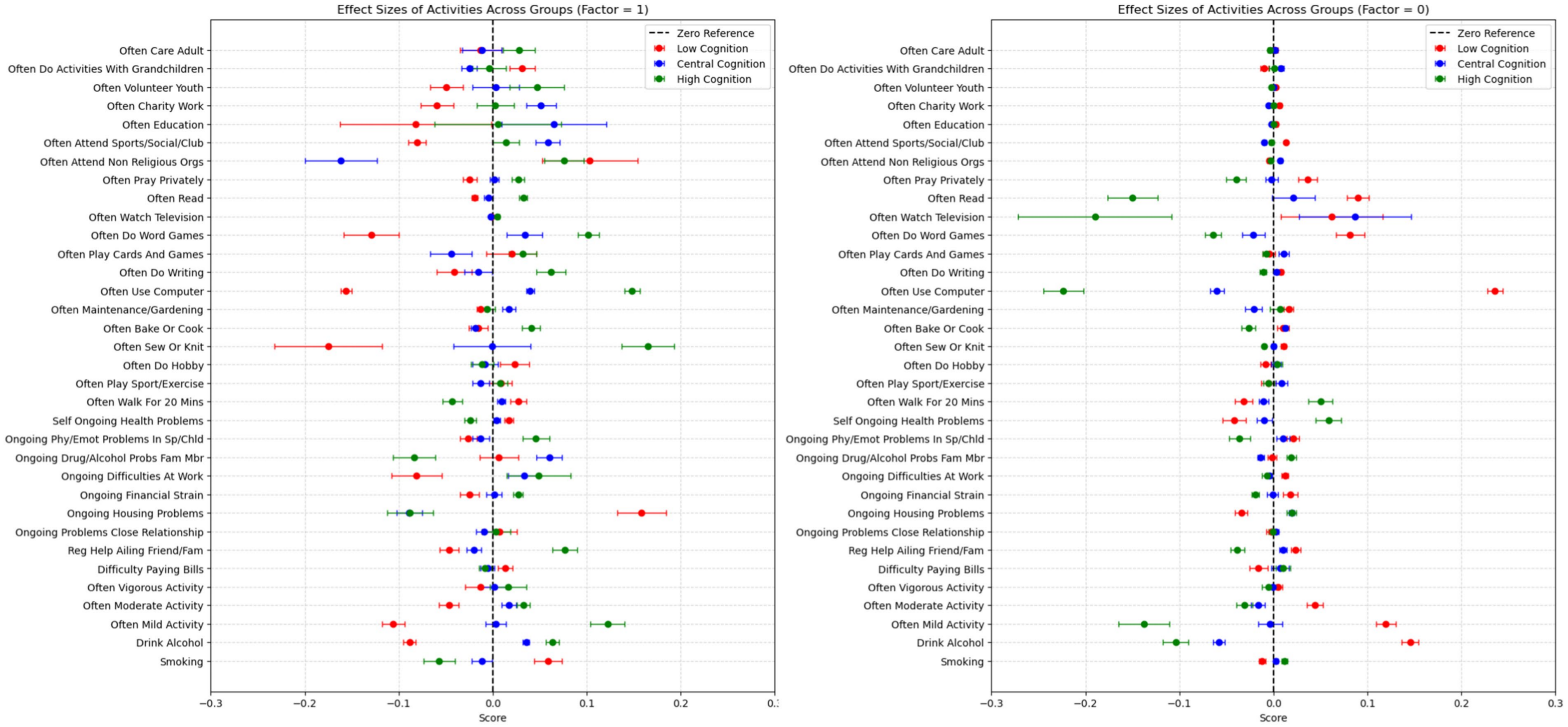
Weights associated to each variable when bin is 1 (Left) or 0 (Right). X scores between −1 and 0 correspond to a negative contribution to that group. X scores between 0 and 1 correspond to a positive contribution to that group. The legend refers to the cognitive groups – low cognition, central cognition and high cognition. The features are plotted by order of importance as reported in Supplementary Figure 2.

In this work, we investigated the associations between environmental factors and cognitive performance using EBM and compared its performance and interpretability with LR, RF, SVM, XGB and MLP models on a sample of 3,482 healthy older adults from the HRS. We adopted a cross-sectional approach, where both cognitive performance and covariates were measured at the same wave. Here, we report the global explanations obtained from the EBM model for all 36 variables. As EBM associates a weight with each variable bin, we plotted the obtained weights in Figure 1. In this figure, we observe the magnitude of impact of individual features on each cognitive category. The y-axis reflects the magnitude of impact, which corresponds to the likelihood of class membership, while the x-axis represents features values (for continuous variables such as Age and Education). For binary variables, values on the x-axis between −1 and 0 correspond to the weight for category 0 (NO), while values between 0 and 1 correspond to category 1 (YES). Each line represents a distinct cognitive trajectory. Green scores correspond to the high cognition group, the blue scores correspond to the central cognition group and the red scores correspond to the low cognition group. Each feature is plotted by order of importance, as reported in Supplementary Figure 1. Lastly, horizontal lines represent error bars.

The results presented in Figure 1 suggest that most variables present distinct magnitudes of effects when comparing the presence and absence of specific activities. Further, this difference is evident across the three cognitive groups, with the most pronounced differences observed in the lowest (Cogn 1) and highest (Cogn 3) cognitive categories. This pattern is evident for *Often Use Computer* and *Drinking Alcohol*. Often using a computer (x > 0) is associated with an increased likelihood of being in the highest cognitive category (green scores in Figure 1) while not often using a computer (x < 0) is associated with an increased likelihood of belonging to the lowest cognitive category (red scores in Figure 1). In contrast, drinking alcohol (x > 0) is associated with an increased likelihood of being in the lowest cognitive category whereas not drinking alcohol (x < 0) increases the likelihood of being in the highest cognitive category. Further, our results suggest that increasing age is associated with a higher likelihood of belonging to the lowest performing cognitive group (Cogn 1), whereas lower age is associated with an increased likelihood of belonging the highest cognitive performing group (Cogn 3). For education, we obtained the opposite pattern. For binary variables, often using a computer is associated with a lower likelihood of belonging to the lowest cognitive group and increased likelihood of belonging to the highest cognitive group. The opposite pattern was observed for those who did not use a computer. We obtained similar patterns in our verification analysis with five and nine cognitive groups, as detailed in the Supplementary material (See Supplementary Figures 5, 9).

### Binned covariate analysis

Supplementary Table 2 and Supplementary Figure 4 include the results of the statistical analysis evaluated at 5% for each variable bin and within each cognitive group using Z-tests. For clarity we selected representative bins for “*Age*” (60, 65, 70, 80, and 85) and “*Education*” (10, 12, and 14) and reported the results when the activities are performed (bin 1). For most of the variable categories, the Z-tests were not significant for the central cognitive category (Cogn 2). For some variables such as Often Use Compute, Ongoing Housing Problems, Drink Alcohol, and Often Moderate Activity, Regularly Help Ailing Friend/Family, all variable categories were significant across all cognitive groups, whereas for other variables the categories were significant only for certain cognitive groups. For instance, Often Volunteer with Youth was significant only for the lowest cognitive category and Often Watch Television only for the highest cognitive group. These statistical results also show differences in the impact of doing and not doing an activity. We reported additional statistical results for the remainder of variables and dependent variables in the provided GitHub repository.

### Performance comparisons

The performance of EBM was compared against LR, SVM, RF, XGB and MLP. A 5-fold cross-validation was used and the average area under the ROC curve was reported (ROC AUC; with higher values reflecting a higher performance of model) when the 3 (Cogn3), 5 (Cogn5) and 9 (Cogn9) cognitive groups are considered as dependent variables in Table 1. The obtained results suggest that most of the models achieved similar performances. The Kruskal-Wallis analysis of variance was used to statistically compare the mean scores for each dependent variable. Non-parametric tests were used as the cross-validation standard deviations of the models differ and violate the homoscedasticity criteria to perform a traditional analysis of variance. For all dependent variables, we obtained a *p*-value < 0.05 and rejected the null hypothesis, i.e., equal mean scores for all models.

**Table 1:** Mean and standard deviations of ROC AUC scores obtained over a 5-fold cross-validation and with Cogn_3_, Cogn_5_, and Cogn_9_ as dependent variables.

Models	Cogn_3_	Cogn_5_	Cogn_9_
EBM	**0.669 ± 0.07**	0.653 ± 0.016	0.626 ± 0.008
LR	0.666 ± 0.006	**0.654 ± 0.016**	0.627 ± 0.006
MLP	0.665 ± 0.004	0.649 ± 0.015	0.622 ± 0.011
XGB	0.634 ± 0.015	0.618 ± 0.013	0.595 ± 0.008
SVM	0.665 ± 009	0.647 ± 0.016	**0.63 ± 0.013**
RF	0.657 ± 0.011	0.63 ± 0.019	0.599 ± 0.008

The highest scores per output variable are highlighted in bold. When the mean of two models is equal, we considered the upper bound score (Mean + STD).

Following these results, *post hoc* analyses were performed through Wilcoxon signed-rank tests for pairwise comparisons of the models. The results of the dependent variables Cogn3, Cogn5, and Cogn9 are reported, respectively, in Supplementary Tables 2–4. As shown in Table 2, the Wilcoxon signed-rank tests indicate that no model significantly outperformed another in terms of AUC, suggesting comparable predictive performance across models. Specifically, for the dependent variable Cogn3, EBM, LR, XBG, and RF exhibit similar *p*-values, with higher *p*-values observed for comparisons involving SVC and MLP. A similar trend can be observed in Supplementary Table 3 (Cogn5), where EBM, XGB, MLP, and RF form one group, while LR and SVM form another. In Supplementary Table 4 (Cogn 9), all models (EBM, LR, XGB, RF, MLP, and SVM) show similar *p*-values, reinforcing the lack of significant differences in predictive performance. Table 2 is presented below. Supplementary Tables 3, 4 are presented in the Supplementary material of this manuscript. Given these findings, model selection should consider both predictive performance and interpretability. EBM provides a transparent and explainable approach, making it valuable for understanding cognitive aging. To further support this, we compared EBM’s local explanations with those of LIME and SHAP, which are presented in the Supplementary material.

**Table 2:** *p*-values of Wilcoxon signed rank tests results obtained for the dependent variable Cogn3.

	EBM	XGB	LR	SVM	MLP	RF
EBM	1	0.062	0.062	0.125	0.188	0.062
XGB	0.062	1	0.062	0.062	0.062	0.062
LR	0.062	0.062	1	0.812	0.438	0.062
SVM	0.125	0.062	0.812	1	1	0.062
MLP	0.188	0.062	0.438	1	1	0.125
RF	0.062	0.062	0.062	0.062	0.125	1

### Verification analyses

To verify the patterns obtained in Figure 1 several verification analyses were performed. First, we increased the number of cognitive groups and re-ran the analyses with the EBM model. The results are, respectively, described in Supplementary Figures 5, 6 for Cogn_5_ and Supplementary Figures 9, 10 for Cogn_9_ as dependent variables. We have also included in the Supplementary material the Z-test scores obtained for each independent variable category both for Cogn_5_ (See Supplementary Table 4) and Cogn_9_ (See Supplementary Table 6). For Age and Education, the categories were determined by the EBM model.

Lastly, we calculated the *p*-values for the Wilcoxon signed-rank tests results obtained for the dependent variable Cogn_5_ (See Supplementary Table 3) and for the dependent variable Cogn_9_ (See Supplementary Table 5).

## Discussion and conclusion

This work evaluates the influence of lifestyle factors on maintaining or promoting good cognitive health and investigates their significance in the context of cognitive aging and Explainable AI (XAI). To the best of our knowledge, this is the first application of EBM in this context. The representative sample (reflected in the large sample size), paired with the flexibility of the model, including its ability to model non-linearity, interpretability, and accuracy, enabled a detailed assessment of the magnitude of impact of each factor on cognitive performance within the older adult population.

Through this work, we were able to conduct a comparative analysis to evaluate the trade-offs between predictive performance and interpretability across different models. Our results indicate that EBM performs comparably to both performance of traditional black-box models and interpretable models, while enhanced interpretability. This balance makes EBM a valuable tool for and generating insights into the factors influencing cognitive health in aging. Further, the associations identified in our analysis align with existing literature, reinforcing the robustness of our findings. In specific, findings suggest that engagement in a given lifestyle factor can largely influence the likelihood of being in a specific cognitive category, particularly the lowest and highest cognitive groups (Figure 1). As an example, individuals who do not engage in mild activities (x < 0) have a significantly higher likelihood of being in the lowest cognitive category than in the highest cognitive category. Conversely, engaging in mild activities (x > 0) increases the likelihood of being in the highest cognitive category while reducing the likelihood of being in the lowest cognitive category. A second critical finding lies with the stability of the second cognitive category. Here, results suggest that regardless of whether an individual engages or not in mild activities, the likelihood of increasing or decreasing in cognitive performance never changes. This pattern is consistent across most lifestyle factors, suggesting that while certain behaviors strongly influence cognitive extremes (lowest and highest cognitive groups), their impact on the central cognitive group remains minimal.

Our results indicate that the observed patterns for continuous variables align with what has previously been reported. Specifically, increasing “*Age*” is associated with lower categories of cognitive performance, while increasing levels of “*Education*” appear to be more strongly associated with higher categories of cognitive performance. Among binary variables, those that contribute to the highest cognitive category appear to be mostly associated with concentration-related activities (e.g., “*Often Use Computer*,” “*Often do Word Games*,” and “*Reading*”), exercise-related activities (e.g., “*Mild Activities*” and “*Moderate Activities*”), and social-related activities (e.g., “*Drinking*” and “*Regularly Help Ailing Friends/Family*”). *In contrast,* variables associated to the lowest cognitive category appear to be mostly associated with socioeconomic status such as “*Ongoing Housing Problems*.”

Our results indicate that for certain factors, the detrimental effects on cognitive health are more pronounced when these activities are neglected compared to the beneficial effects observed when they are actively pursued. For example, the magnitude of not using a computer in the lowest and highest cognitive groups is greater than the magnitude of using a computer in these groups. Conversely, for other factors, the reverse pattern is observed, where the adverse effects outweigh the benefits. This is evident in variables such as *“Ongoing Housing Problems,” “Smoking,” “Ongoing Health Problems,”* and *“Difficulty Paying Bills”* (see Supplementary Figure 4; Supplementary Table 2), where the negative impact of these experiences is greater than the positive contribution of not experiencing them. The stability observed in the central cognitive category across most factors suggests that these influences remain constant, regardless of whether they are being performed or not. The increased susceptibility observed in the lowest and highest categories, compared to the resistance observed in the central category, may be indicative of subtypes of the population previously described in studies of aging (Rodrigues et al., 2022). These differences in response may represent subtypes of individuals with distinct susceptibilities to environmental exposures (such as lifestyle factors). In previous studies these subtypes have been described as environment-sensitive (lowest and highest cognitive groups), that experience greater cognitive gains but also greater cognitive losses depending on external conditions, and environment-resistant (central cognitive group), whose cognitive performance remains stable regardless of whether external conditions are optimal or detrimental (but are never as good or as bad as environment-sensitive individuals) (Rodrigues et al., 2022).

These findings indicate that the effects of environmental factors on cognitive health are not uniform, highlighting differential impacts across individuals. Identifying these variations can inform the development of targeted interventions, allowing for more personalized strategies to promote cognitive well-being among older adults. The comparative analysis of predictive models indicates that EBM effectively captures non-linear interactions while maintaining interpretability. While its predictive performance was comparable to other models, its ability to provide granular feature attributions and interpretable decision-making offers valuable insights into the determinants of cognitive aging.

The success of EBM model in our study underscores the significance of advancing Explainable AI in cognitive health research. Unlike black-box models such as gradient boosting decision trees and deep neural networks, EBM provides interpretability alongside competitive predictive accuracy, addressing the need for transparency in healthcare decision-making. This aligns with the broader trajectory of Explainable AI, toward developing models that are transparent and interpretable in real-world applications. Additionally, our findings highlight that a one-size-fits-all approach to promoting cognitive health may be insufficient. The personalized insights derived from this study emphasize the importance of tailoring interventions based on individual profiles and specific environmental factors. This shift toward precision medicine in cognitive aging emphasizes the need for personalized healthcare strategies to improve cognitive outcomes in aging populations.

In conclusion, this study examines the relationship between environmental factors and cognitive performance while demonstrating the application of Explainable AI techniques, particularly the EBM model, in cognitive aging research. By using an interpretable machine learning approach, we provide a framework for assessing nonlinear interactions between cognitive aging predictors while maintaining model transparency. The findings highlight the advantages of using XAI models in aging research, where interpretability is essential for both scientific validation and practical applications in healthcare and policy.

Some limitations have been identified in the present work. Theoretical limitations include the fact that GAMs, including EBM, can produce different interpretations depending on the feature mapping function used (e.g., splines and gradient boosting machines) (Chang et al., 2021). Consequently, our results might not be reproducible with different mapping functions. Additionally, like many machine learning models, EBM is sensitive to sample size, resulting in high standard deviations of the scores (logits) in variable ranges with low data as observed for “*Age”* and “*Education*” (see Supplementary Figures 2, 3, respectively). Practical limitations include the use of default hyperparameters when comparing EBM to other models (LR, MLP, SVM, RF, and XGB), which could affect both their performance and training time. In addition, the study focuses on a population of healthy older adults that participate in large longitudinal studies. These individuals tend to be healthier, more educated and of higher socioeconomic status than average, limiting generalizability of results.

An additional limitation includes the predictive performance of all models which was modest. This likely reflects the inherent complexity of cognitive health outcomes in aging. The fact that EBM achieved AUC values comparable to these models suggests that it can provide useful insights while maintaining interpretability. However, we acknowledge that further improvements in predictive performance may require incorporating additional features or alternative modeling approaches.

The findings of this study have implications for cognitive health policy and decision-making. This study demonstrates how interpretable machine learning models can be applied in cognitive aging research to provide transparent and reproducible insights. The ability of interpretable AI models to provide transparent results can support targeted interventions for cognitive maintenance and consequently independent living. By leveraging interpretable machine learning, future work focused on healthcare systems can develop data-driven prevention strategies, improve screening protocols, and optimize resource allocation. Additionally, the integration of XAI into public health decision-making can improve trust and accountability in AI-driven healthcare applications, ensuring compliance with regulatory and ethical standards. The continued development of XAI in aging research may enhance both scientific understanding and its practical applications in healthcare and policy-making.

## Data Availability

The original contributions presented in the study are included in the article/Supplementary material, further inquiries can be directed to the corresponding author.

## Footnotes

^1^ https://github.com/abdjiber/Interpretable-Machine-Learning-for-Precision-Aging
