# Supplementary material for: Interpretable machine learning for precision cognitive aging
Source: Front Comput Neurosci. 2025 May 16;19:1560064. doi: 10.3389/fncom.2025.1560064 (PMC12122752; doi:10.3389/fncom.2025.1560064)
Supplement: Supplementary file 1 [file Supplementary_file_1.docx]

**Interpretable Machine Learning for Precision Cognitive Aging**

**Supplementary Material**

Abdoul Jalil Djiberou Mahamadou*^1^, Emma Antunes Rodrigues*^2^, Vasily Vakorin^2^,

Violaine Antoine^3^, Sylvain Moreno^2,4,†^

Appendix

*Independent Variable Selection*

We selected 34 lifestyle activities, background factors and socio-economic status from the HRS database in addition to ”*Age*” and “*Years of Education*”. The selection of each variable is based on previous studies and details can be found in (20).

Supplementary Table 1 - List of covariates

| **INDEPENDENT VARIABLE** | | |
| --- | --- | --- |
| **Ongoing health problems** | **Often read** | **Age** |
| **Ongoing physical/emotional problem in spouse/child** | **Often watch television** | **Years of Education** |
| **Ongoing drug/alcohol problem with family member** | **Often do word games** | **Smokes** |
| **Ongoing difficulties at work** | **Often play cards and games** | **Drinks** |
| **Ongoing financial strain** | **Often do writing** | **Often do hobby** |
| **Ongoing housing problem** | **Often use computer** | **Often Care Adult** |
| **Ongoing problems in close relationship** | **Often do maintenance/gardening** | **Ongoing Difficulty Paying Bills** |
| **Often do activities with grandchildren** | **Often bake/cook** | **Often Attend Sports/Socials/Clubs** |
| **Often volunteer with youth** | **Often sew/knit** | **Often Play Sports/Exercise** |
| **Often do charity work** | **Often walk for 20 min** | **Regularly Help Ailing Friends/Family** |
| **Often do education courses** | **Often do Vigorous Activities** |  |
| **Often attend non-religious organizations** | **Often do Moderate Activities** |  |
| **Often pray privately** | **Often do Mild Activities** |  |

*Feature Importance*

Supplementary Figure 2 describes the feature importance obtained from the EBM model where the importance corresponds to a weighted mean over all cognitive groups of the scores (logits) associated to each variable bin. A limitation of such importance is that bins with low (resp. high) frequencies in the dataset may be result in a low (resp. high) feature importance. We used the importance in the main manuscript only for a display purpose the variables.


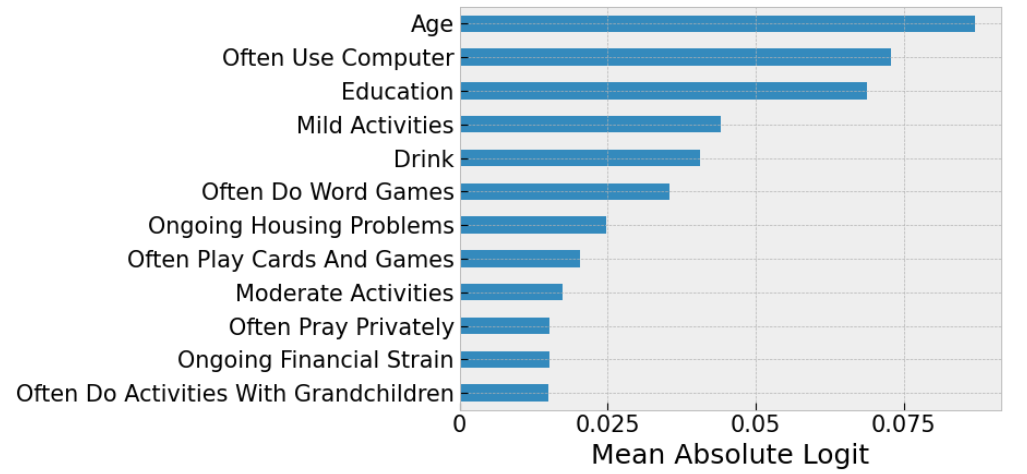


Supplementary Figure 1: Obtained feature importance from the EBM model. The x-axis corresponds to weighted mean absolute logit associated to variable bins.


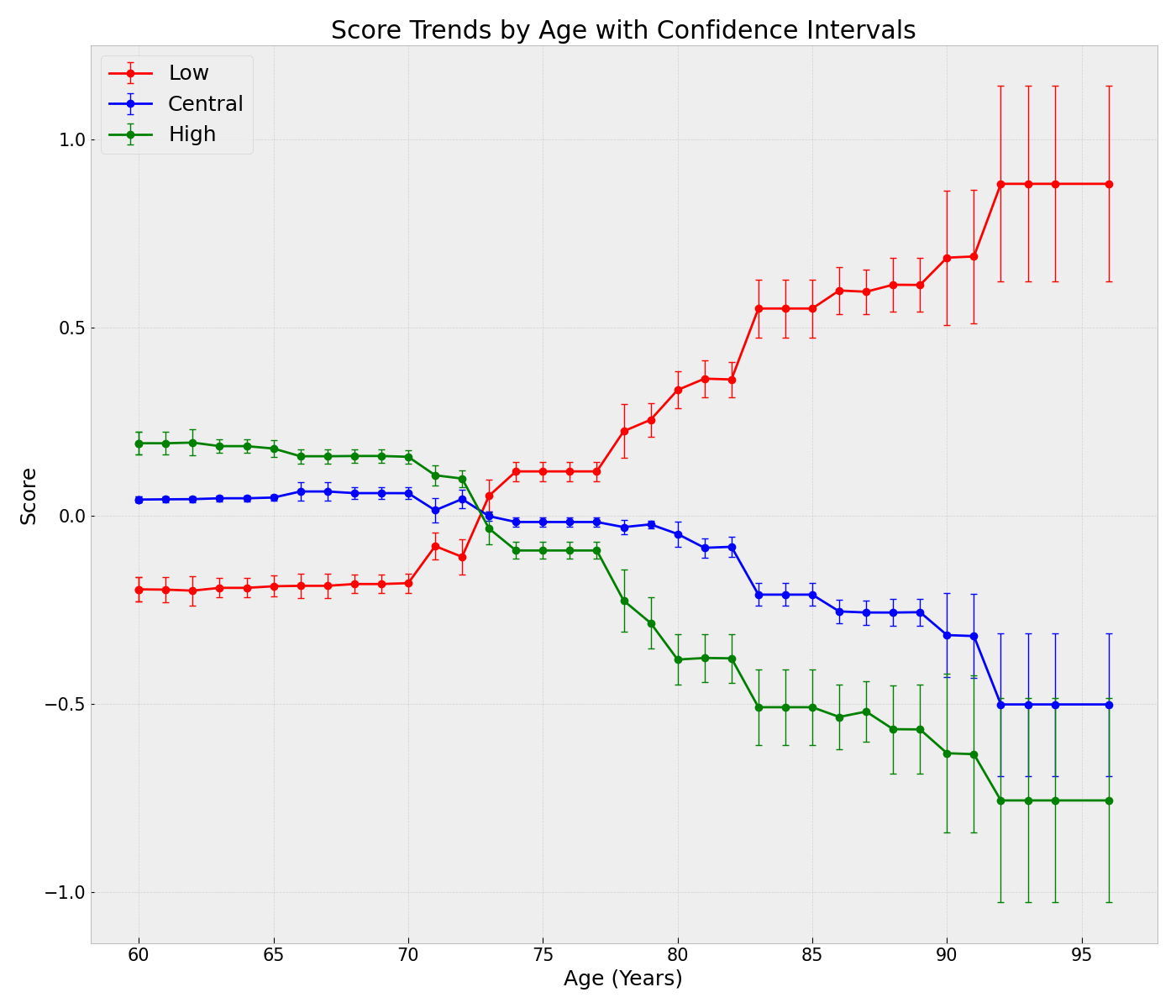


Supplementary Figure 2: Score trends by Age with confidence intervals. The Low, Central, and High correspond to individuals in cognitive categories 1, 2, and 3.


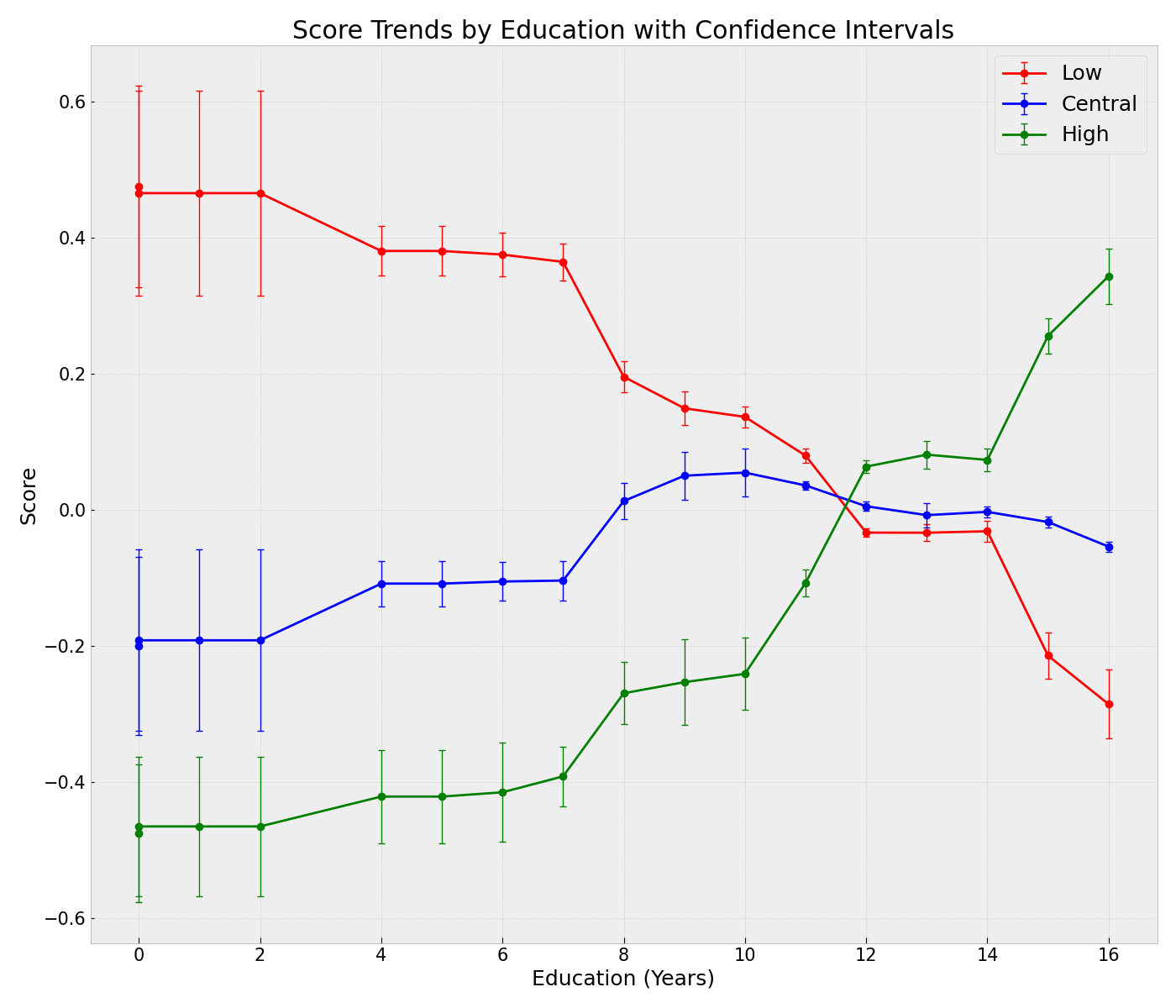


Supplementary Figure 3: Score trends by Education with confidence intervals. The Low, Central, and High correspond to individuals in cognitive categories 1, 2, and 3.


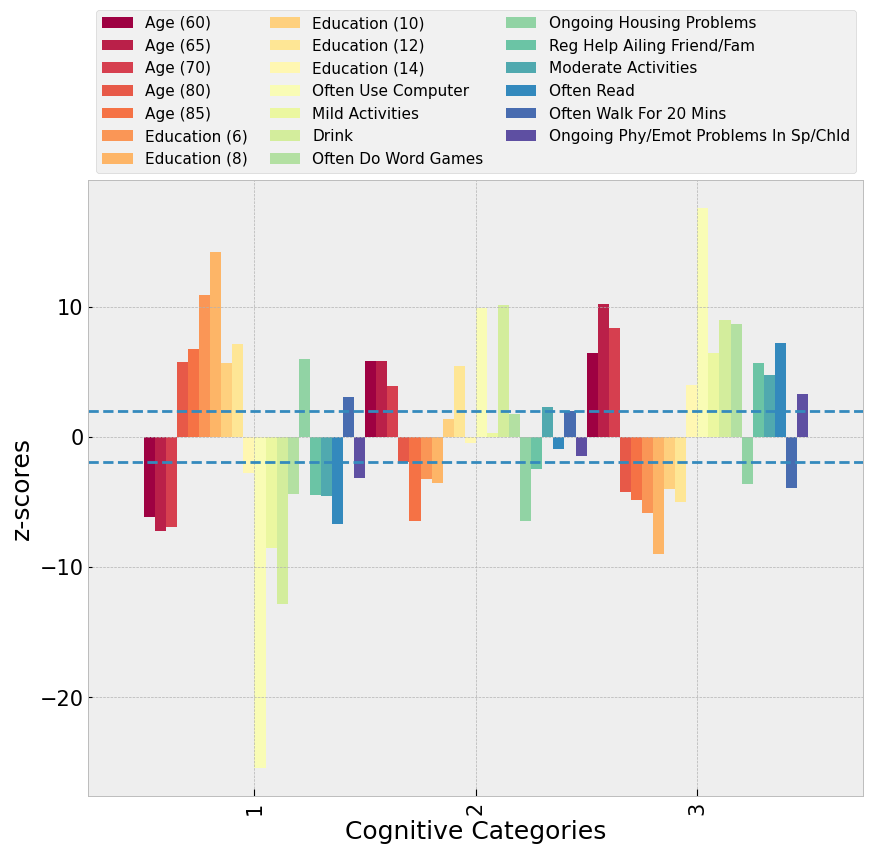


Supplementary Figure 4: Statistical analysis results obtained for the 12th most important variables when performing an activity. The horizontal dotted lines correspond to the significance thresholds of the Z-scores at 5% level (1.96 and -1.96). Variables within these thresholds of the Z-scores at 5% level (1.96 and -1.96). Variables within these thresholds are non-significant.

Supplementary Table 2: Z-test scores obtained for each independent variable category. For Age and Education, the categories were automatedly determined by the EBM model.

|  | **Cogn 1** | | | | **Cogn 2** | | | | **Cogn 3** | | | |
| --- | --- | --- | --- | --- | --- | --- | --- | --- | --- | --- | --- | --- |
|  | **Coeff.** | **Std. Err.** | **z** | **P>\|z\|** | **Coeff.** | **Std. Err.** | **z** | **P>\|z\|** | **Coeff.** | **Std. Err.** | **z** | **P>\|z\|** |
| **Often Care Adult (0.0)** | 0.002 | 0.003 | 0.591 | 0.555 | 0.001 | 0.003 | 0.558 | 0.577 | -0.004 | 0.002 | -1.493 | 0.135 |
| **Often Care Adult (1.0)** | -0.013 | 0.022 | -0.588 | 0.557 | -0.012 | 0.021 | -0.566 | 0.571 | 0.028 | 0.017 | 1.63 | 0.103 |
| **Often Do Activities With Grandchildren (0.0)** | -0.01 | 0.004 | -2.255 | 0.024 | 0.008 | 0.003 | 2.999 | 0.003 | 0.001 | 0.006 | 0.199 | 0.842 |
| **Often Do Activities With Grandchildren (1.0)** | 0.031 | 0.014 | 2.287 | 0.022 | -0.025 | 0.008 | -3.001 | 0.003 | -0.004 | 0.018 | -0.207 | 0.836 |
| **Often Volunteer Youth (0.0)** | 0.002 | 0.001 | 2.858 | 0.004 | -0.0 | 0.001 | -0.127 | 0.899 | -0.002 | 0.002 | -1.403 | 0.161 |
| **Often Volunteer Youth (1.0)** | -0.049 | 0.018 | -2.797 | 0.005 | 0.003 | 0.025 | 0.131 | 0.896 | 0.047 | 0.029 | 1.624 | 0.104 |
| **Often Charity Work (0.0)** | 0.006 | 0.002 | 3.757 | 0.0 | -0.006 | 0.002 | -3.239 | 0.001 | -0.0 | 0.002 | -0.123 | 0.902 |
| **Often Charity Work (1.0)** | -0.059 | 0.018 | -3.373 | 0.001 | 0.051 | 0.016 | 3.229 | 0.001 | 0.003 | 0.02 | 0.135 | 0.893 |
| **Often Education (0.0)** | 0.002 | 0.002 | 1.147 | 0.251 | -0.002 | 0.002 | -1.145 | 0.252 | -0.0 | 0.002 | -0.078 | 0.938 |
| **Often Education (1.0)** | -0.082 | 0.081 | -1.014 | 0.311 | 0.065 | 0.056 | 1.161 | 0.246 | 0.005 | 0.068 | 0.08 | 0.936 |
| **Often Attend Sports/Social/Club (0.0)** | 0.013 | 0.001 | 10.612 | 0.0 | -0.01 | 0.002 | -4.487 | 0.0 | -0.002 | 0.003 | -0.918 | 0.359 |
| **Often Attend Sports/Social/Club (1.0)** | -0.08 | 0.009 | -8.853 | 0.0 | 0.059 | 0.013 | 4.484 | 0.0 | 0.014 | 0.014 | 1.023 | 0.306 |
| **Often Attend Non Religious Orgs (0.0)** | -0.005 | 0.002 | -1.988 | 0.047 | 0.007 | 0.002 | 4.341 | 0.0 | -0.003 | 0.001 | -3.254 | 0.001 |
| **Often Attend Non Religious Orgs (1.0)** | 0.103 | 0.051 | 2.029 | 0.042 | -0.162 | 0.038 | -4.219 | 0.0 | 0.076 | 0.021 | 3.607 | 0.0 |
| **Often Pray Privately (0.0)** | 0.036 | 0.01 | 3.539 | 0.0 | -0.002 | 0.007 | -0.288 | 0.773 | -0.04 | 0.01 | -3.867 | 0.0 |
| **Often Pray Privately (1.0)** | -0.024 | 0.007 | -3.345 | 0.001 | 0.001 | 0.005 | 0.284 | 0.776 | 0.027 | 0.007 | 4.077 | 0.0 |
| **Often Read (0.0)** | 0.09 | 0.012 | 7.656 | 0.0 | 0.021 | 0.023 | 0.931 | 0.352 | -0.15 | 0.027 | -5.578 | 0.0 |
| **Often Read (1.0)** | -0.019 | 0.003 | -6.805 | 0.0 | -0.005 | 0.005 | -0.954 | 0.34 | 0.032 | 0.004 | 7.283 | 0.0 |
| **Often Watch Television (0.0)** | 0.062 | 0.054 | 1.147 | 0.251 | 0.087 | 0.059 | 1.461 | 0.144 | -0.19 | 0.082 | -2.331 | 0.02 |
| **Often Watch Television (1.0)** | -0.002 | 0.001 | -1.11 | 0.267 | -0.002 | 0.001 | -1.533 | 0.125 | 0.005 | 0.002 | 2.662 | 0.008 |
| **Often Do Word Games (0.0)** | 0.082 | 0.015 | 5.315 | 0.0 | -0.021 | 0.012 | -1.764 | 0.078 | -0.064 | 0.008 | -7.829 | 0.0 |
| **Often Do Word Games (1.0)** | -0.129 | 0.029 | -4.43 | 0.0 | 0.034 | 0.019 | 1.762 | 0.078 | 0.102 | 0.011 | 9.178 | 0.0 |
| **Often Play Cards And Games (0.0)** | -0.005 | 0.007 | -0.763 | 0.445 | 0.011 | 0.005 | 2.016 | 0.044 | -0.008 | 0.004 | -1.961 | 0.05 |
| **Often Play Cards And Games (1.0)** | 0.02 | 0.027 | 0.75 | 0.453 | -0.044 | 0.022 | -1.995 | 0.046 | 0.032 | 0.015 | 2.148 | 0.032 |
| **Often Do Writing (0.0)** | 0.007 | 0.003 | 2.506 | 0.012 | 0.003 | 0.003 | 1.088 | 0.277 | -0.011 | 0.003 | -3.504 | 0.0 |
| **Often Do Writing (1.0)** | -0.041 | 0.019 | -2.219 | 0.026 | -0.015 | 0.015 | -1.036 | 0.3 | 0.062 | 0.016 | 3.941 | 0.0 |
| **Often Use Computer (0.0)** | 0.236 | 0.008 | 28.183 | 0.0 | -0.06 | 0.007 | -8.435 | 0.0 | -0.223 | 0.021 | -10.444 | 0.0 |
| **Often Use Computer (1.0)** | -0.156 | 0.006 | -26.694 | 0.0 | 0.04 | 0.004 | 9.484 | 0.0 | 0.148 | 0.008 | 18.219 | 0.0 |
| **Often Maintenance/Gardening (0.0)** | 0.016 | 0.005 | 3.179 | 0.001 | -0.021 | 0.009 | -2.344 | 0.019 | 0.007 | 0.011 | 0.642 | 0.521 |
| **Often Maintenance/Gardening (1.0)** | -0.013 | 0.004 | -2.956 | 0.003 | 0.017 | 0.007 | 2.421 | 0.015 | -0.006 | 0.008 | -0.726 | 0.468 |
| **Often Bake Or Cook (0.0)** | 0.01 | 0.006 | 1.591 | 0.112 | 0.012 | 0.003 | 4.477 | 0.0 | -0.027 | 0.007 | -3.589 | 0.0 |
| **Often Bake Or Cook (1.0)** | -0.015 | 0.01 | -1.504 | 0.133 | -0.019 | 0.004 | -4.627 | 0.0 | 0.041 | 0.01 | 4.06 | 0.0 |
| **Often Sew Or Knit (0.0)** | 0.011 | 0.003 | 3.992 | 0.0 | 0.0 | 0.002 | 0.014 | 0.989 | -0.01 | 0.002 | -5.407 | 0.0 |
| **Often Sew Or Knit (1.0)** | -0.175 | 0.057 | -3.061 | 0.002 | -0.001 | 0.041 | -0.013 | 0.99 | 0.165 | 0.028 | 5.984 | 0.0 |
| **Often Do Hobby (0.0)** | -0.009 | 0.005 | -1.659 | 0.097 | 0.003 | 0.005 | 0.603 | 0.547 | 0.004 | 0.005 | 0.797 | 0.425 |
| **Often Do Hobby (1.0)** | 0.023 | 0.015 | 1.504 | 0.133 | -0.008 | 0.013 | -0.606 | 0.545 | -0.011 | 0.012 | -0.912 | 0.362 |
| **Often Play Sport/Exercise (0.0)** | -0.006 | 0.008 | -0.729 | 0.466 | 0.009 | 0.006 | 1.41 | 0.159 | -0.005 | 0.006 | -0.88 | 0.379 |
| **Often Play Sport/Exercise (1.0)** | 0.008 | 0.012 | 0.674 | 0.5 | -0.013 | 0.009 | -1.44 | 0.15 | 0.008 | 0.008 | 1.0 | 0.317 |
| **Often Walk For 20 Mins (0.0)** | -0.032 | 0.009 | -3.439 | 0.001 | -0.01 | 0.005 | -1.999 | 0.046 | 0.05 | 0.013 | 3.924 | 0.0 |
| **Often Walk For 20 Mins (1.0)** | 0.027 | 0.008 | 3.238 | 0.001 | 0.009 | 0.004 | 1.992 | 0.046 | -0.043 | 0.01 | -4.136 | 0.0 |
| **Self Ongoing Health Problems (0.0)** | -0.042 | 0.012 | -3.39 | 0.001 | -0.01 | 0.008 | -1.169 | 0.242 | 0.059 | 0.014 | 4.32 | 0.0 |
| **Self Ongoing Health Problems (1.0)** | 0.017 | 0.005 | 3.596 | 0.0 | 0.004 | 0.004 | 1.124 | 0.261 | -0.024 | 0.006 | -3.716 | 0.0 |
| **Ongoing Phy/Emot Problems In Sp/Chld (0.0)** | 0.021 | 0.007 | 2.965 | 0.003 | 0.01 | 0.007 | 1.441 | 0.15 | -0.036 | 0.011 | -3.19 | 0.001 |
| **Ongoing Phy/Emot Problems In Sp/Chld (1.0)** | -0.026 | 0.009 | -2.99 | 0.003 | -0.013 | 0.009 | -1.458 | 0.145 | 0.046 | 0.014 | 3.224 | 0.001 |
| **Ongoing Drug/Alcohol Probs Fam Mbr (0.0)** | -0.001 | 0.005 | -0.303 | 0.762 | -0.014 | 0.003 | -4.26 | 0.0 | 0.019 | 0.005 | 3.857 | 0.0 |
| **Ongoing Drug/Alcohol Probs Fam Mbr (1.0)** | 0.006 | 0.021 | 0.31 | 0.757 | 0.06 | 0.013 | 4.483 | 0.0 | -0.084 | 0.022 | -3.736 | 0.0 |
| **Ongoing Difficulties At Work (0.0)** | 0.012 | 0.004 | 3.243 | 0.001 | -0.005 | 0.003 | -1.95 | 0.051 | -0.007 | 0.005 | -1.344 | 0.179 |
| **Ongoing Difficulties At Work (1.0)** | -0.081 | 0.027 | -3.033 | 0.002 | 0.033 | 0.017 | 1.979 | 0.048 | 0.049 | 0.034 | 1.442 | 0.149 |
| **Ongoing Financial Strain (0.0)** | 0.018 | 0.008 | 2.377 | 0.017 | -0.001 | 0.006 | -0.16 | 0.873 | -0.02 | 0.004 | -5.252 | 0.0 |
| **Ongoing Financial Strain (1.0)** | -0.025 | 0.01 | -2.476 | 0.013 | 0.001 | 0.008 | 0.16 | 0.873 | 0.027 | 0.005 | 5.1 | 0.0 |
| **Ongoing Housing Problems (0.0)** | -0.034 | 0.007 | -5.127 | 0.0 | 0.019 | 0.003 | 6.78 | 0.0 | 0.019 | 0.005 | 3.918 | 0.0 |
| **Ongoing Housing Problems (1.0)** | 0.158 | 0.026 | 6.165 | 0.0 | -0.089 | 0.013 | -6.656 | 0.0 | -0.088 | 0.024 | -3.655 | 0.0 |
| **Ongoing Problems Close Relationship (0.0)** | -0.002 | 0.006 | -0.378 | 0.705 | 0.003 | 0.003 | 1.045 | 0.296 | -0.001 | 0.005 | -0.21 | 0.834 |
| **Ongoing Problems Close Relationship (1.0)** | 0.007 | 0.019 | 0.386 | 0.699 | -0.009 | 0.009 | -1.039 | 0.299 | 0.003 | 0.015 | 0.218 | 0.827 |
| **Reg Help Ailing Friend/Fam (0.0)** | 0.024 | 0.005 | 4.706 | 0.0 | 0.01 | 0.004 | 2.511 | 0.012 | -0.039 | 0.007 | -5.224 | 0.0 |
| **Reg Help Ailing Friend/Fam (1.0)** | -0.047 | 0.01 | -4.58 | 0.0 | -0.02 | 0.008 | -2.482 | 0.013 | 0.077 | 0.013 | 5.901 | 0.0 |
| **Difficulty Paying Bills (0.0)** | -0.016 | 0.01 | -1.657 | 0.098 | 0.007 | 0.009 | 0.777 | 0.437 | 0.01 | 0.008 | 1.329 | 0.184 |
| **Difficulty Paying Bills (1.0)** | 0.013 | 0.008 | 1.767 | 0.077 | -0.006 | 0.007 | -0.772 | 0.44 | -0.008 | 0.007 | -1.241 | 0.215 |
| **Often Vigorous Activity (0.0)** | 0.004 | 0.005 | 0.951 | 0.342 | -0.0 | 0.005 | -0.087 | 0.931 | -0.005 | 0.007 | -0.723 | 0.47 |
| **Often Vigorous Activity (1.0)** | -0.013 | 0.016 | -0.854 | 0.393 | 0.001 | 0.014 | 0.088 | 0.93 | 0.016 | 0.019 | 0.835 | 0.404 |
| **Often Moderate Activity (0.0)** | 0.044 | 0.009 | 5.095 | 0.0 | -0.016 | 0.007 | -2.222 | 0.026 | -0.031 | 0.008 | -3.778 | 0.0 |
| **Often Moderate Activity (1.0)** | -0.047 | 0.01 | -4.515 | 0.0 | 0.017 | 0.008 | 2.276 | 0.023 | 0.033 | 0.007 | 4.755 | 0.0 |
| **Often Mild Activity (0.0)** | 0.12 | 0.011 | 11.19 | 0.0 | -0.004 | 0.013 | -0.282 | 0.778 | -0.138 | 0.027 | -5.031 | 0.0 |
| **Often Mild Activity (1.0)** | -0.106 | 0.012 | -8.844 | 0.0 | 0.003 | 0.011 | 0.296 | 0.767 | 0.122 | 0.018 | 6.685 | 0.0 |
| **Drink Alcohol (0.0)** | 0.145 | 0.009 | 16.216 | 0.0 | -0.058 | 0.006 | -9.666 | 0.0 | -0.104 | 0.013 | -7.752 | 0.0 |
| **Drink Alcohol (1.0)** | -0.089 | 0.007 | -13.2 | 0.0 | 0.035 | 0.004 | 9.84 | 0.0 | 0.063 | 0.007 | 9.321 | 0.0 |
| **Education (0.0)** | 0.475 | 0.148 | 3.212 | 0.001 | -0.2 | 0.131 | -1.524 | 0.128 | -0.476 | 0.102 | -4.675 | 0.0 |
| **Education (0.5)** | 0.466 | 0.15 | 3.098 | 0.002 | -0.192 | 0.133 | -1.438 | 0.15 | -0.466 | 0.103 | -4.539 | 0.0 |
| **Education (1.5)** | 0.466 | 0.15 | 3.098 | 0.002 | -0.192 | 0.133 | -1.438 | 0.15 | -0.466 | 0.103 | -4.539 | 0.0 |
| **Education (2.5)** | 0.466 | 0.15 | 3.098 | 0.002 | -0.192 | 0.133 | -1.438 | 0.15 | -0.466 | 0.103 | -4.539 | 0.0 |
| **Education (4.5)** | 0.381 | 0.036 | 10.51 | 0.0 | -0.109 | 0.033 | -3.269 | 0.001 | -0.422 | 0.069 | -6.131 | 0.0 |
| **Education (5.5)** | 0.381 | 0.036 | 10.51 | 0.0 | -0.109 | 0.033 | -3.269 | 0.001 | -0.422 | 0.069 | -6.131 | 0.0 |
| **Education (6.5)** | 0.375 | 0.032 | 11.549 | 0.0 | -0.105 | 0.028 | -3.746 | 0.0 | -0.415 | 0.073 | -5.727 | 0.0 |
| **Education (7.5)** | 0.365 | 0.027 | 13.273 | 0.0 | -0.104 | 0.029 | -3.59 | 0.0 | -0.392 | 0.044 | -8.986 | 0.0 |
| **Education (8.5)** | 0.196 | 0.022 | 8.709 | 0.0 | 0.013 | 0.026 | 0.49 | 0.624 | -0.27 | 0.046 | -5.875 | 0.0 |
| **Education (9.5)** | 0.149 | 0.025 | 5.943 | 0.0 | 0.05 | 0.035 | 1.413 | 0.158 | -0.254 | 0.063 | -4.031 | 0.0 |
| **Education (10.5)** | 0.137 | 0.016 | 8.731 | 0.0 | 0.055 | 0.035 | 1.572 | 0.116 | -0.241 | 0.053 | -4.542 | 0.0 |
| **Education (11.5)** | 0.08 | 0.01 | 7.655 | 0.0 | 0.036 | 0.007 | 5.513 | 0.0 | -0.108 | 0.02 | -5.318 | 0.0 |
| **Education (12.5)** | -0.034 | 0.007 | -5.068 | 0.0 | 0.005 | 0.007 | 0.747 | 0.455 | 0.063 | 0.009 | 6.871 | 0.0 |
| **Education (13.5)** | -0.034 | 0.012 | -2.782 | 0.005 | -0.008 | 0.018 | -0.438 | 0.661 | 0.081 | 0.02 | 3.96 | 0.0 |
| **Education (14.5)** | -0.032 | 0.016 | -2.012 | 0.044 | -0.003 | 0.008 | -0.394 | 0.694 | 0.073 | 0.017 | 4.303 | 0.0 |
| **Education (15.5)** | -0.214 | 0.034 | -6.309 | 0.0 | -0.018 | 0.008 | -2.171 | 0.03 | 0.256 | 0.026 | 9.875 | 0.0 |
| **Education (16.5)** | -0.286 | 0.05 | -5.663 | 0.0 | -0.054 | 0.008 | -7.215 | 0.0 | 0.344 | 0.041 | 8.462 | 0.0 |
| **Education (17.0)** | -0.286 | 0.05 | -5.663 | 0.0 | -0.054 | 0.008 | -7.215 | 0.0 | 0.344 | 0.041 | 8.462 | 0.0 |
| **Smoking (0.0)** | -0.012 | 0.003 | -3.505 | 0.0 | 0.002 | 0.002 | 1.057 | 0.291 | 0.012 | 0.003 | 3.725 | 0.0 |
| **Smoking (1.0)** | 0.059 | 0.015 | 3.959 | 0.0 | -0.012 | 0.011 | -1.079 | 0.281 | -0.057 | 0.017 | -3.41 | 0.001 |
| **Age (60.0)** | -0.196 | 0.032 | -6.111 | 0.0 | 0.043 | 0.008 | 5.498 | 0.0 | 0.193 | 0.03 | 6.463 | 0.0 |
| **Age (60.5)** | -0.196 | 0.032 | -6.111 | 0.0 | 0.043 | 0.008 | 5.498 | 0.0 | 0.193 | 0.03 | 6.463 | 0.0 |
| **Age (61.5)** | -0.197 | 0.033 | -5.936 | 0.0 | 0.043 | 0.008 | 5.388 | 0.0 | 0.193 | 0.03 | 6.345 | 0.0 |
| **Age (62.5)** | -0.199 | 0.039 | -5.076 | 0.0 | 0.044 | 0.008 | 5.553 | 0.0 | 0.194 | 0.035 | 5.579 | 0.0 |
| **Age (63.5)** | -0.192 | 0.026 | -7.43 | 0.0 | 0.046 | 0.008 | 5.706 | 0.0 | 0.185 | 0.018 | 10.554 | 0.0 |
| **Age (64.5)** | -0.192 | 0.026 | -7.43 | 0.0 | 0.046 | 0.008 | 5.706 | 0.0 | 0.185 | 0.018 | 10.554 | 0.0 |
| **Age (65.5)** | -0.188 | 0.028 | -6.695 | 0.0 | 0.048 | 0.008 | 5.995 | 0.0 | 0.178 | 0.022 | 8.234 | 0.0 |
| **Age (66.5)** | -0.186 | 0.033 | -5.703 | 0.0 | 0.064 | 0.025 | 2.597 | 0.009 | 0.158 | 0.019 | 8.461 | 0.0 |
| **Age (67.5)** | -0.186 | 0.033 | -5.703 | 0.0 | 0.064 | 0.025 | 2.597 | 0.009 | 0.158 | 0.019 | 8.461 | 0.0 |
| **Age (68.5)** | -0.182 | 0.025 | -7.362 | 0.0 | 0.06 | 0.015 | 3.982 | 0.0 | 0.159 | 0.018 | 8.615 | 0.0 |
| **Age (69.5)** | -0.182 | 0.025 | -7.362 | 0.0 | 0.06 | 0.015 | 3.982 | 0.0 | 0.159 | 0.018 | 8.615 | 0.0 |
| **Age (70.5)** | -0.179 | 0.025 | -7.051 | 0.0 | 0.06 | 0.015 | 3.942 | 0.0 | 0.157 | 0.017 | 9.057 | 0.0 |
| **Age (71.5)** | -0.081 | 0.036 | -2.232 | 0.026 | 0.014 | 0.031 | 0.457 | 0.648 | 0.107 | 0.027 | 4.002 | 0.0 |
| **Age (72.5)** | -0.109 | 0.047 | -2.321 | 0.02 | 0.044 | 0.024 | 1.835 | 0.067 | 0.099 | 0.023 | 4.354 | 0.0 |
| **Age (73.5)** | 0.053 | 0.043 | 1.23 | 0.219 | -0.001 | 0.013 | -0.1 | 0.92 | -0.034 | 0.042 | -0.804 | 0.421 |
| **Age (74.5)** | 0.118 | 0.026 | 4.53 | 0.0 | -0.017 | 0.013 | -1.276 | 0.202 | -0.092 | 0.022 | -4.132 | 0.0 |
| **Age (75.5)** | 0.118 | 0.026 | 4.53 | 0.0 | -0.017 | 0.013 | -1.276 | 0.202 | -0.092 | 0.022 | -4.132 | 0.0 |
| **Age (76.5)** | 0.118 | 0.026 | 4.53 | 0.0 | -0.017 | 0.013 | -1.276 | 0.202 | -0.092 | 0.022 | -4.132 | 0.0 |
| **Age (77.5)** | 0.118 | 0.026 | 4.53 | 0.0 | -0.017 | 0.013 | -1.276 | 0.202 | -0.092 | 0.022 | -4.132 | 0.0 |
| **Age (78.5)** | 0.225 | 0.072 | 3.127 | 0.002 | -0.03 | 0.02 | -1.528 | 0.127 | -0.226 | 0.083 | -2.724 | 0.006 |
| **Age (79.5)** | 0.255 | 0.045 | 5.697 | 0.0 | -0.023 | 0.011 | -2.183 | 0.029 | -0.285 | 0.068 | -4.217 | 0.0 |
| **Age (80.5)** | 0.334 | 0.049 | 6.832 | 0.0 | -0.049 | 0.033 | -1.454 | 0.146 | -0.382 | 0.067 | -5.678 | 0.0 |
| **Age (81.5)** | 0.364 | 0.049 | 7.388 | 0.0 | -0.085 | 0.026 | -3.314 | 0.001 | -0.378 | 0.063 | -5.992 | 0.0 |
| **Age (82.5)** | 0.362 | 0.047 | 7.782 | 0.0 | -0.083 | 0.026 | -3.199 | 0.001 | -0.379 | 0.065 | -5.874 | 0.0 |
| **Age (83.5)** | 0.551 | 0.077 | 7.164 | 0.0 | -0.21 | 0.03 | -6.913 | 0.0 | -0.509 | 0.1 | -5.076 | 0.0 |
| **Age (84.5)** | 0.551 | 0.077 | 7.164 | 0.0 | -0.21 | 0.03 | -6.913 | 0.0 | -0.509 | 0.1 | -5.076 | 0.0 |
| **Age (85.5)** | 0.551 | 0.077 | 7.164 | 0.0 | -0.21 | 0.03 | -6.913 | 0.0 | -0.509 | 0.1 | -5.076 | 0.0 |
| **Age (86.5)** | 0.599 | 0.062 | 9.671 | 0.0 | -0.254 | 0.032 | -7.992 | 0.0 | -0.535 | 0.086 | -6.225 | 0.0 |
| **Age (87.5)** | 0.595 | 0.06 | 9.982 | 0.0 | -0.257 | 0.032 | -7.982 | 0.0 | -0.521 | 0.08 | -6.524 | 0.0 |
| **Age (88.5)** | 0.614 | 0.072 | 8.475 | 0.0 | -0.257 | 0.035 | -7.32 | 0.0 | -0.568 | 0.117 | -4.844 | 0.0 |
| **Age (89.5)** | 0.613 | 0.072 | 8.543 | 0.0 | -0.257 | 0.036 | -7.155 | 0.0 | -0.568 | 0.118 | -4.809 | 0.0 |
| **Age (90.5)** | 0.686 | 0.179 | 3.835 | 0.0 | -0.317 | 0.111 | -2.848 | 0.004 | -0.631 | 0.211 | -2.997 | 0.003 |
| **Age (91.5)** | 0.689 | 0.177 | 3.891 | 0.0 | -0.32 | 0.111 | -2.882 | 0.004 | -0.634 | 0.208 | -3.041 | 0.002 |
| **Age (92.5)** | 0.882 | 0.26 | 3.393 | 0.001 | -0.502 | 0.19 | -2.647 | 0.008 | -0.757 | 0.271 | -2.788 | 0.005 |
| **Age (93.5)** | 0.882 | 0.26 | 3.393 | 0.001 | -0.502 | 0.19 | -2.647 | 0.008 | -0.757 | 0.271 | -2.788 | 0.005 |
| **Age (94.5)** | 0.882 | 0.26 | 3.393 | 0.001 | -0.502 | 0.19 | -2.647 | 0.008 | -0.757 | 0.271 | -2.788 | 0.005 |
| **Age (96.0)** | 0.882 | 0.26 | 3.393 | 0.001 | -0.502 | 0.19 | -2.647 | 0.008 | -0.757 | 0.271 | -2.788 | 0.005 |
| **Age (99.0)** | 0.882 | 0.26 | 3.393 | 0.001 | -0.502 | 0.19 | -2.647 | 0.008 | -0.757 | 0.271 | -2.788 | 0.005 |

*Verification Analyses*

To verify the patterns obtained in Figure 1 of the main manuscript we performed several verification analyses. First, we increased the number of cognitive groups and rerun the analysis with the EBM model. The results are respectively described in Figure 5 and Figure 6.

- 5 Cognitive Groups


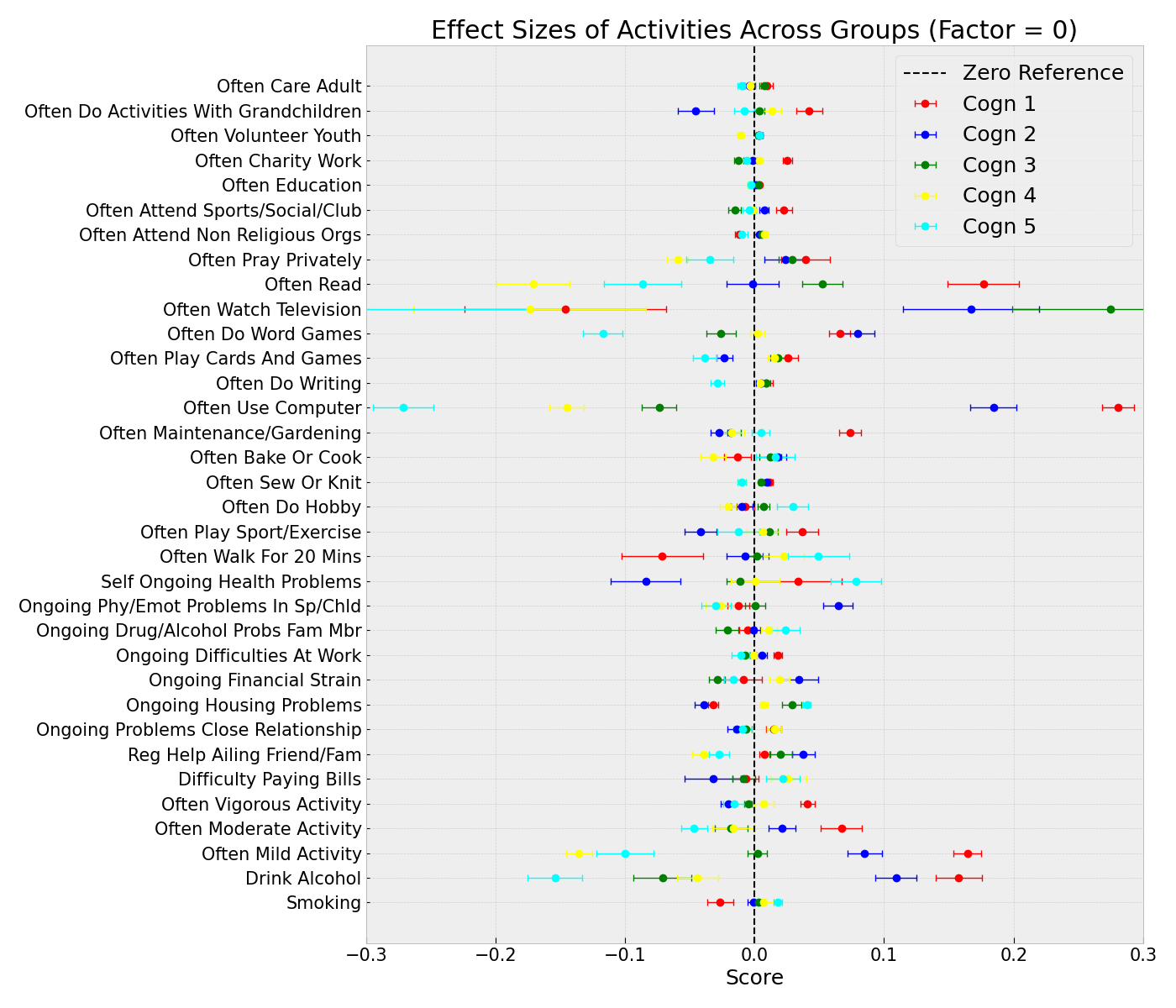


Supplementary Figure 5: Patterns obtained for all variables with the EBM model with factor 0 (not doing an activity) and 5 cognitive categories as dependent variable. The order of the variables is arbitrary.


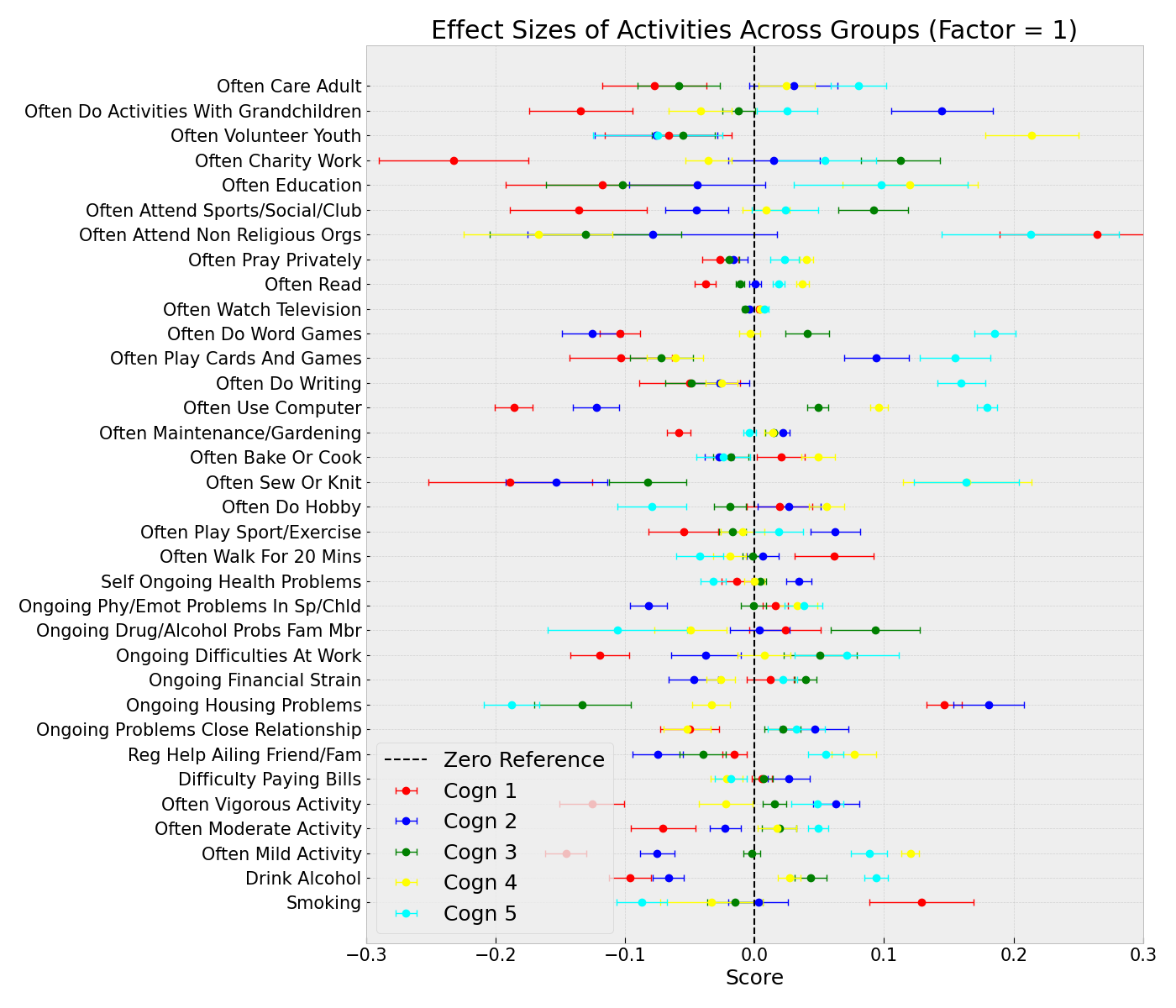


Supplementary Figure 6: Patterns obtained for all variables with the EBM model with factor 1 (doing an activity) and 5 cognitive categories as dependent variable. The order of the variables is arbitrary.


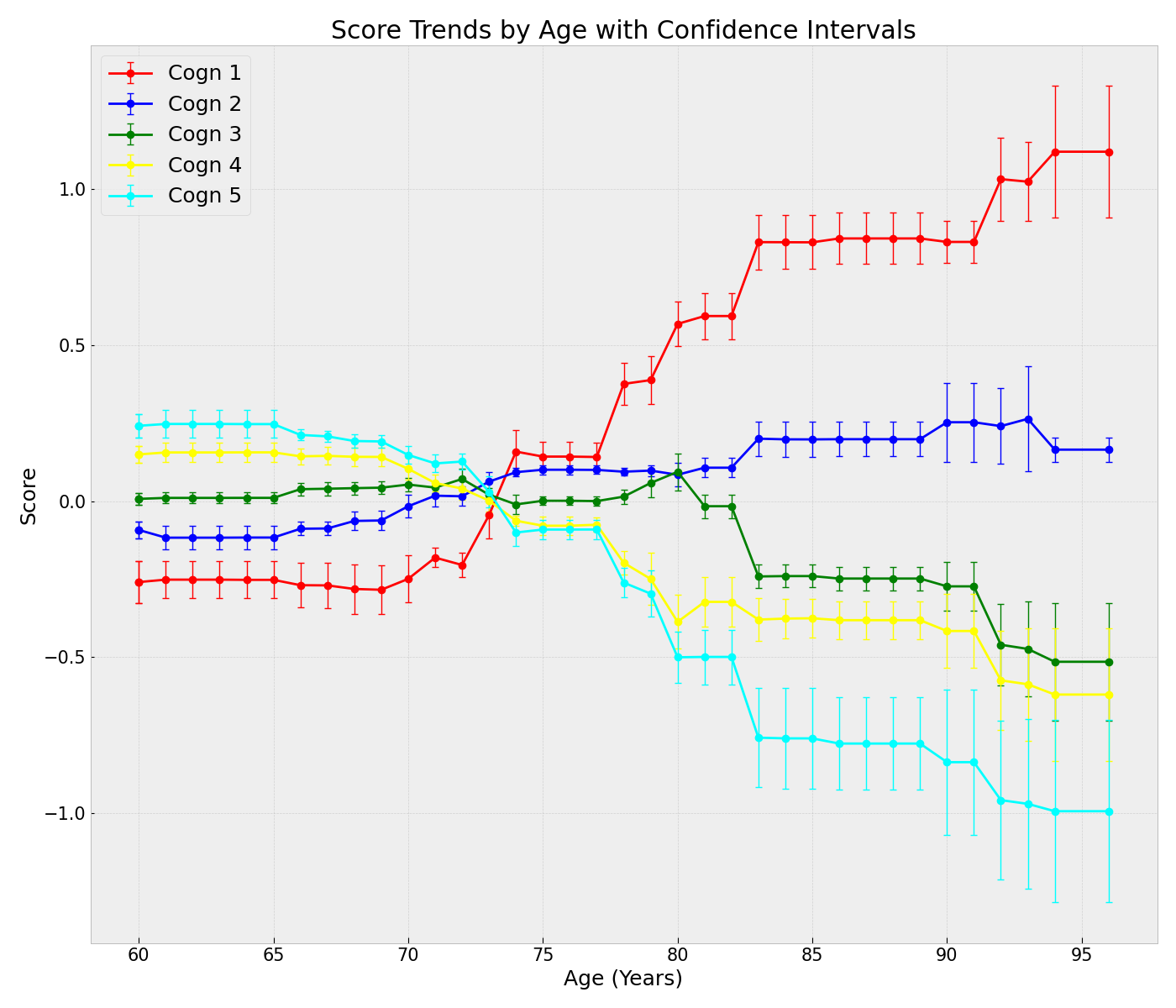


Supplementary Figure 7: Score trends by Age with confidence intervals of five cognitive categories.


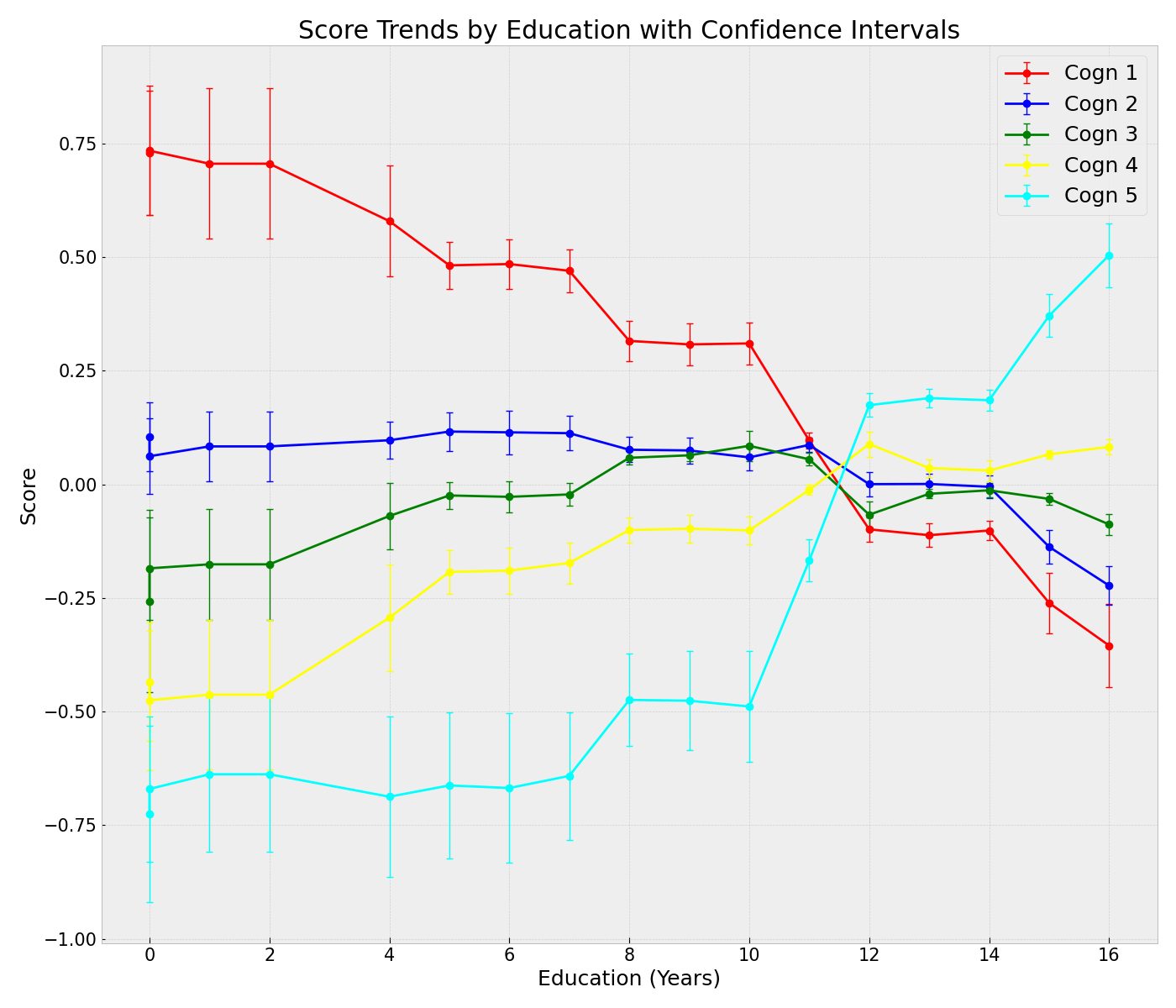


Supplementary Figure 8: Score trends by Education with confidence intervals of five cognitive categories.

Supplementary Table 3: P-values of Wilcoxon signed rank tests results obtained for the dependent variable Cogn5

|  | EBM | XGB | LR | SVC | MLP | RF |
| --- | --- | --- | --- | --- | --- | --- |
| EBM | 1 | 0.062 | 0.438 | 0.188 | 0.062 | 0.062 |
| XGB | 0.062 | 1 | 0.062 | 0.062 | 0.062 | 0.062 |
| LR | 0.438 | 0.062 | 1 | 0.125 | 0.062 | 0.062 |
| SVC | 0.188 | 0.062 | 0.125 | 1 | 1 | 0.062 |
| MLP | 0.062 | 0.062 | 0.062 | 1 | 1 | 0.062 |
| RF | 0.062 | 0.062 | 0.062 | 0.062 | 0.062 | 1 |

Supplementary Table 4: Z-test scores obtained for each independent variable category. For Age and Education, the categories were automatedly determined by the EBM model.

|  | **Cogn 1** | | | | **Cogn 2** | | | | **Cogn 3** | | | | **Cogn 4** | | | | **Cogn 5** | | | |
| --- | --- | --- | --- | --- | --- | --- | --- | --- | --- | --- | --- | --- | --- | --- | --- | --- | --- | --- | --- | --- |
|  | **Coeff.** | **Std. Err.** | **z** | **P>\|z\|** | **Coeff.** | **Std. Err.** | **z** | **P>\|z\|** | **Coeff.** | **Std. Err.** | **z** | **P>\|z\|** | **Coeff.** | **Std. Err.** | **z** | **P>\|z\|** | **Coeff.** | **Std. Err.** | **z** | **P>\|z\|** |
| **Often Care Adult (0.0)** | 0.01 | 0.004 | 2.183 | 0.029 | -0.004 | 0.004 | -0.914 | 0.361 | 0.007 | 0.004 | 1.949 | 0.051 | -0.003 | 0.003 | -1.042 | 0.297 | -0.01 | 0.003 | -3.34 | 0.001 |
| **Often Care Adult (1.0)** | -0.077 | 0.04 | -1.935 | 0.053 | 0.03 | 0.034 | 0.879 | 0.379 | -0.059 | 0.032 | -1.831 | 0.067 | 0.025 | 0.022 | 1.144 | 0.253 | 0.08 | 0.022 | 3.721 | 0.0 |
| **Often Do Activities With Grandchildren (0.0)** | 0.042 | 0.01 | 4.145 | 0.0 | -0.045 | 0.014 | -3.274 | 0.001 | 0.004 | 0.004 | 0.988 | 0.323 | 0.013 | 0.008 | 1.717 | 0.086 | -0.008 | 0.008 | -0.998 | 0.318 |
| **Often Do Activities With Grandchildren (1.0)** | -0.134 | 0.04 | -3.37 | 0.001 | 0.145 | 0.039 | 3.698 | 0.0 | -0.012 | 0.013 | -0.96 | 0.337 | -0.042 | 0.024 | -1.721 | 0.085 | 0.025 | 0.023 | 1.081 | 0.28 |
| **Often Volunteer Youth (0.0)** | 0.003 | 0.002 | 1.741 | 0.082 | 0.004 | 0.002 | 1.713 | 0.087 | 0.003 | 0.001 | 2.371 | 0.018 | -0.011 | 0.002 | -4.726 | 0.0 | 0.004 | 0.003 | 1.341 | 0.18 |
| **Often Volunteer Youth (1.0)** | -0.067 | 0.049 | -1.355 | 0.175 | -0.076 | 0.047 | -1.607 | 0.108 | -0.055 | 0.024 | -2.247 | 0.025 | 0.214 | 0.036 | 5.945 | 0.0 | -0.075 | 0.05 | -1.496 | 0.135 |
| **Often Charity Work (0.0)** | 0.025 | 0.004 | 6.864 | 0.0 | -0.002 | 0.004 | -0.461 | 0.645 | -0.012 | 0.004 | -3.39 | 0.001 | 0.004 | 0.002 | 1.84 | 0.066 | -0.006 | 0.005 | -1.146 | 0.252 |
| **Often Charity Work (1.0)** | -0.232 | 0.058 | -4.035 | 0.0 | 0.015 | 0.035 | 0.424 | 0.672 | 0.113 | 0.031 | 3.677 | 0.0 | -0.036 | 0.018 | -2.011 | 0.044 | 0.054 | 0.039 | 1.392 | 0.164 |
| **Often Education (0.0)** | 0.003 | 0.002 | 2.157 | 0.031 | 0.001 | 0.001 | 0.973 | 0.331 | 0.003 | 0.002 | 1.807 | 0.071 | -0.004 | 0.002 | -2.14 | 0.032 | -0.003 | 0.003 | -1.127 | 0.26 |
| **Often Education (1.0)** | -0.118 | 0.074 | -1.588 | 0.112 | -0.044 | 0.052 | -0.842 | 0.4 | -0.102 | 0.059 | -1.725 | 0.085 | 0.12 | 0.052 | 2.289 | 0.022 | 0.097 | 0.067 | 1.447 | 0.148 |
| **Often Attend Sports/Social/Club (0.0)** | 0.023 | 0.006 | 3.725 | 0.0 | 0.007 | 0.004 | 2.086 | 0.037 | -0.015 | 0.005 | -3.266 | 0.001 | -0.001 | 0.003 | -0.451 | 0.652 | -0.004 | 0.005 | -0.748 | 0.454 |
| **Often Attend Sports/Social/Club (1.0)** | -0.136 | 0.053 | -2.57 | 0.01 | -0.045 | 0.024 | -1.847 | 0.065 | 0.092 | 0.027 | 3.422 | 0.001 | 0.009 | 0.018 | 0.483 | 0.629 | 0.024 | 0.025 | 0.925 | 0.355 |
| **Often Attend Non Religious Orgs (0.0)** | -0.012 | 0.003 | -3.68 | 0.0 | 0.004 | 0.004 | 0.901 | 0.368 | 0.006 | 0.003 | 1.986 | 0.047 | 0.008 | 0.003 | 2.85 | 0.004 | -0.01 | 0.004 | -2.424 | 0.015 |
| **Often Attend Non Religious Orgs (1.0)** | 0.264 | 0.075 | 3.524 | 0.0 | -0.079 | 0.096 | -0.819 | 0.413 | -0.13 | 0.074 | -1.764 | 0.078 | -0.167 | 0.058 | -2.901 | 0.004 | 0.213 | 0.068 | 3.114 | 0.002 |
| **Often Pray Privately (0.0)** | 0.039 | 0.019 | 2.068 | 0.039 | 0.024 | 0.016 | 1.477 | 0.14 | 0.029 | 0.011 | 2.756 | 0.006 | -0.059 | 0.008 | -7.246 | 0.0 | -0.034 | 0.018 | -1.905 | 0.057 |
| **Often Pray Privately (1.0)** | -0.027 | 0.014 | -1.906 | 0.057 | -0.016 | 0.011 | -1.466 | 0.143 | -0.02 | 0.008 | -2.595 | 0.009 | 0.04 | 0.005 | 8.039 | 0.0 | 0.023 | 0.011 | 2.057 | 0.04 |
| **Often Read (0.0)** | 0.177 | 0.028 | 6.392 | 0.0 | -0.001 | 0.02 | -0.063 | 0.95 | 0.052 | 0.015 | 3.413 | 0.001 | -0.171 | 0.028 | -6.101 | 0.0 | -0.086 | 0.03 | -2.912 | 0.004 |
| **Often Read (1.0)** | -0.038 | 0.008 | -4.713 | 0.0 | 0.0 | 0.005 | 0.059 | 0.953 | -0.011 | 0.003 | -3.331 | 0.001 | 0.037 | 0.005 | 7.656 | 0.0 | 0.019 | 0.005 | 3.949 | 0.0 |
| **Often Watch Television (0.0)** | -0.146 | 0.078 | -1.875 | 0.061 | 0.167 | 0.052 | 3.193 | 0.001 | 0.274 | 0.076 | 3.629 | 0.0 | -0.174 | 0.09 | -1.938 | 0.053 | -0.305 | 0.13 | -2.341 | 0.019 |
| **Often Watch Television (1.0)** | 0.004 | 0.002 | 2.14 | 0.032 | -0.004 | 0.002 | -2.841 | 0.004 | -0.007 | 0.002 | -3.068 | 0.002 | 0.005 | 0.002 | 2.338 | 0.019 | 0.008 | 0.003 | 2.857 | 0.004 |
| **Often Do Word Games (0.0)** | 0.066 | 0.008 | 8.349 | 0.0 | 0.079 | 0.013 | 5.918 | 0.0 | -0.026 | 0.011 | -2.3 | 0.021 | 0.002 | 0.005 | 0.423 | 0.672 | -0.117 | 0.015 | -7.644 | 0.0 |
| **Often Do Word Games (1.0)** | -0.104 | 0.015 | -6.725 | 0.0 | -0.125 | 0.023 | -5.35 | 0.0 | 0.041 | 0.017 | 2.427 | 0.015 | -0.004 | 0.008 | -0.453 | 0.651 | 0.186 | 0.016 | 11.868 | 0.0 |
| **Often Play Cards And Games (0.0)** | 0.026 | 0.008 | 3.22 | 0.001 | -0.023 | 0.006 | -3.741 | 0.0 | 0.018 | 0.006 | 3.047 | 0.002 | 0.015 | 0.005 | 2.995 | 0.003 | -0.038 | 0.009 | -4.306 | 0.0 |
| **Often Play Cards And Games (1.0)** | -0.103 | 0.039 | -2.617 | 0.009 | 0.094 | 0.025 | 3.795 | 0.0 | -0.072 | 0.024 | -2.972 | 0.003 | -0.061 | 0.022 | -2.819 | 0.005 | 0.155 | 0.027 | 5.683 | 0.0 |
| **Often Do Writing (0.0)** | 0.009 | 0.005 | 1.66 | 0.097 | 0.005 | 0.004 | 1.288 | 0.198 | 0.009 | 0.003 | 2.541 | 0.011 | 0.005 | 0.002 | 1.884 | 0.06 | -0.029 | 0.005 | -5.81 | 0.0 |
| **Often Do Writing (1.0)** | -0.05 | 0.039 | -1.28 | 0.201 | -0.027 | 0.023 | -1.166 | 0.244 | -0.049 | 0.02 | -2.456 | 0.014 | -0.025 | 0.012 | -2.041 | 0.041 | 0.16 | 0.018 | 8.671 | 0.0 |
| **Often Use Computer (0.0)** | 0.281 | 0.012 | 22.636 | 0.0 | 0.185 | 0.018 | 10.314 | 0.0 | -0.074 | 0.013 | -5.519 | 0.0 | -0.145 | 0.014 | -10.731 | 0.0 | -0.271 | 0.023 | -11.574 | 0.0 |
| **Often Use Computer (1.0)** | -0.186 | 0.015 | -12.592 | 0.0 | -0.122 | 0.018 | -6.821 | 0.0 | 0.049 | 0.008 | 6.065 | 0.0 | 0.096 | 0.007 | 13.789 | 0.0 | 0.179 | 0.008 | 23.006 | 0.0 |
| **Often Maintenance/Gardening (0.0)** | 0.074 | 0.008 | 8.831 | 0.0 | -0.027 | 0.006 | -4.345 | 0.0 | -0.019 | 0.008 | -2.325 | 0.02 | -0.018 | 0.01 | -1.806 | 0.071 | 0.005 | 0.007 | 0.685 | 0.493 |
| **Often Maintenance/Gardening (1.0)** | -0.059 | 0.009 | -6.474 | 0.0 | 0.022 | 0.005 | 4.052 | 0.0 | 0.015 | 0.006 | 2.28 | 0.023 | 0.014 | 0.007 | 2.059 | 0.039 | -0.004 | 0.005 | -0.774 | 0.439 |
| **Often Bake Or Cook (0.0)** | -0.013 | 0.01 | -1.271 | 0.204 | 0.018 | 0.007 | 2.648 | 0.008 | 0.012 | 0.009 | 1.398 | 0.162 | -0.032 | 0.01 | -3.276 | 0.001 | 0.016 | 0.015 | 1.054 | 0.292 |
| **Often Bake Or Cook (1.0)** | 0.02 | 0.019 | 1.091 | 0.275 | -0.027 | 0.011 | -2.485 | 0.013 | -0.018 | 0.013 | -1.375 | 0.169 | 0.049 | 0.013 | 3.748 | 0.0 | -0.024 | 0.021 | -1.169 | 0.242 |
| **Often Sew Or Knit (0.0)** | 0.011 | 0.003 | 4.338 | 0.0 | 0.009 | 0.002 | 4.896 | 0.0 | 0.005 | 0.002 | 3.082 | 0.002 | -0.01 | 0.003 | -2.962 | 0.003 | -0.01 | 0.003 | -3.101 | 0.002 |
| **Often Sew Or Knit (1.0)** | -0.189 | 0.063 | -2.986 | 0.003 | -0.153 | 0.039 | -3.92 | 0.0 | -0.082 | 0.03 | -2.774 | 0.006 | 0.164 | 0.05 | 3.314 | 0.001 | 0.163 | 0.041 | 4.027 | 0.0 |
| **Often Do Hobby (0.0)** | -0.007 | 0.007 | -1.033 | 0.302 | -0.01 | 0.008 | -1.173 | 0.241 | 0.007 | 0.005 | 1.537 | 0.124 | -0.02 | 0.006 | -3.329 | 0.001 | 0.029 | 0.012 | 2.459 | 0.014 |
| **Often Do Hobby (1.0)** | 0.019 | 0.025 | 0.769 | 0.442 | 0.027 | 0.024 | 1.099 | 0.272 | -0.019 | 0.012 | -1.539 | 0.124 | 0.055 | 0.014 | 4.033 | 0.0 | -0.079 | 0.027 | -2.998 | 0.003 |
| **Often Play Sport/Exercise (0.0)** | 0.037 | 0.012 | 2.96 | 0.003 | -0.042 | 0.012 | -3.395 | 0.001 | 0.011 | 0.007 | 1.614 | 0.107 | 0.006 | 0.012 | 0.515 | 0.607 | -0.012 | 0.016 | -0.754 | 0.451 |
| **Often Play Sport/Exercise (1.0)** | -0.055 | 0.027 | -2.048 | 0.041 | 0.062 | 0.019 | 3.247 | 0.001 | -0.017 | 0.01 | -1.616 | 0.106 | -0.009 | 0.017 | -0.565 | 0.572 | 0.018 | 0.019 | 0.957 | 0.339 |
| **Often Walk For 20 Mins (0.0)** | -0.071 | 0.031 | -2.265 | 0.024 | -0.007 | 0.014 | -0.525 | 0.6 | 0.002 | 0.009 | 0.216 | 0.829 | 0.022 | 0.016 | 1.445 | 0.148 | 0.049 | 0.024 | 2.092 | 0.036 |
| **Often Walk For 20 Mins (1.0)** | 0.061 | 0.031 | 2.006 | 0.045 | 0.006 | 0.013 | 0.498 | 0.618 | -0.002 | 0.008 | -0.217 | 0.828 | -0.019 | 0.012 | -1.553 | 0.12 | -0.042 | 0.018 | -2.354 | 0.019 |
| **Self Ongoing Health Problems (0.0)** | 0.034 | 0.033 | 1.009 | 0.313 | -0.084 | 0.027 | -3.132 | 0.002 | -0.011 | 0.011 | -1.051 | 0.293 | 0.0 | 0.019 | 0.021 | 0.983 | 0.078 | 0.02 | 4.0 | 0.0 |
| **Self Ongoing Health Problems (1.0)** | -0.014 | 0.012 | -1.165 | 0.244 | 0.034 | 0.01 | 3.544 | 0.0 | 0.005 | 0.004 | 1.045 | 0.296 | -0.0 | 0.008 | -0.02 | 0.984 | -0.032 | 0.01 | -3.355 | 0.001 |
| **Ongoing Phy/Emot Problems In Sp/Chld (0.0)** | -0.013 | 0.008 | -1.518 | 0.129 | 0.065 | 0.011 | 5.652 | 0.0 | 0.001 | 0.008 | 0.089 | 0.929 | -0.026 | 0.012 | -2.184 | 0.029 | -0.03 | 0.011 | -2.61 | 0.009 |
| **Ongoing Phy/Emot Problems In Sp/Chld (1.0)** | 0.016 | 0.01 | 1.626 | 0.104 | -0.082 | 0.014 | -5.743 | 0.0 | -0.001 | 0.01 | -0.089 | 0.929 | 0.033 | 0.015 | 2.206 | 0.027 | 0.038 | 0.014 | 2.633 | 0.008 |
| **Ongoing Drug/Alcohol Probs Fam Mbr (0.0)** | -0.005 | 0.006 | -0.83 | 0.407 | -0.001 | 0.005 | -0.172 | 0.863 | -0.021 | 0.009 | -2.416 | 0.016 | 0.011 | 0.006 | 1.798 | 0.072 | 0.024 | 0.011 | 2.201 | 0.028 |
| **Ongoing Drug/Alcohol Probs Fam Mbr (1.0)** | 0.024 | 0.027 | 0.856 | 0.392 | 0.004 | 0.023 | 0.171 | 0.864 | 0.093 | 0.035 | 2.697 | 0.007 | -0.049 | 0.028 | -1.76 | 0.078 | -0.106 | 0.054 | -1.962 | 0.05 |
| **Ongoing Difficulties At Work (0.0)** | 0.018 | 0.003 | 5.937 | 0.0 | 0.006 | 0.004 | 1.54 | 0.124 | -0.008 | 0.004 | -1.805 | 0.071 | -0.001 | 0.003 | -0.359 | 0.72 | -0.011 | 0.007 | -1.564 | 0.118 |
| **Ongoing Difficulties At Work (1.0)** | -0.12 | 0.023 | -5.231 | 0.0 | -0.038 | 0.027 | -1.395 | 0.163 | 0.051 | 0.028 | 1.803 | 0.071 | 0.008 | 0.02 | 0.369 | 0.712 | 0.071 | 0.04 | 1.775 | 0.076 |
| **Ongoing Financial Strain (0.0)** | -0.009 | 0.014 | -0.624 | 0.533 | 0.035 | 0.014 | 2.409 | 0.016 | -0.029 | 0.006 | -4.725 | 0.0 | 0.019 | 0.008 | 2.51 | 0.012 | -0.016 | 0.007 | -2.23 | 0.026 |
| **Ongoing Financial Strain (1.0)** | 0.012 | 0.018 | 0.673 | 0.501 | -0.047 | 0.019 | -2.452 | 0.014 | 0.039 | 0.009 | 4.642 | 0.0 | -0.026 | 0.011 | -2.403 | 0.016 | 0.022 | 0.01 | 2.108 | 0.035 |
| **Ongoing Housing Problems (0.0)** | -0.032 | 0.004 | -8.111 | 0.0 | -0.039 | 0.007 | -5.644 | 0.0 | 0.029 | 0.007 | 3.84 | 0.0 | 0.007 | 0.003 | 2.476 | 0.013 | 0.041 | 0.003 | 13.712 | 0.0 |
| **Ongoing Housing Problems (1.0)** | 0.147 | 0.014 | 10.783 | 0.0 | 0.18 | 0.027 | 6.621 | 0.0 | -0.133 | 0.037 | -3.574 | 0.0 | -0.033 | 0.015 | -2.282 | 0.022 | -0.188 | 0.021 | -8.877 | 0.0 |
| **Ongoing Problems Close Relationship (0.0)** | 0.015 | 0.006 | 2.446 | 0.014 | -0.014 | 0.008 | -1.788 | 0.074 | -0.006 | 0.004 | -1.466 | 0.143 | 0.015 | 0.006 | 2.777 | 0.005 | -0.009 | 0.007 | -1.347 | 0.178 |
| **Ongoing Problems Close Relationship (1.0)** | -0.05 | 0.023 | -2.213 | 0.027 | 0.046 | 0.026 | 1.76 | 0.078 | 0.022 | 0.014 | 1.55 | 0.121 | -0.052 | 0.018 | -2.84 | 0.005 | 0.032 | 0.022 | 1.446 | 0.148 |
| **Reg Help Ailing Friend/Fam (0.0)** | 0.008 | 0.004 | 1.813 | 0.07 | 0.038 | 0.009 | 4.257 | 0.0 | 0.02 | 0.009 | 2.336 | 0.019 | -0.039 | 0.01 | -4.0 | 0.0 | -0.028 | 0.008 | -3.537 | 0.0 |
| **Reg Help Ailing Friend/Fam (1.0)** | -0.016 | 0.009 | -1.649 | 0.099 | -0.075 | 0.019 | -3.827 | 0.0 | -0.04 | 0.018 | -2.256 | 0.024 | 0.077 | 0.017 | 4.491 | 0.0 | 0.055 | 0.014 | 3.992 | 0.0 |
| **Difficulty Paying Bills (0.0)** | -0.007 | 0.01 | -0.675 | 0.5 | -0.032 | 0.022 | -1.477 | 0.14 | -0.008 | 0.009 | -0.932 | 0.351 | 0.026 | 0.014 | 1.851 | 0.064 | 0.022 | 0.013 | 1.663 | 0.096 |
| **Difficulty Paying Bills (1.0)** | 0.006 | 0.008 | 0.739 | 0.46 | 0.027 | 0.016 | 1.646 | 0.1 | 0.007 | 0.007 | 0.938 | 0.348 | -0.022 | 0.012 | -1.738 | 0.082 | -0.018 | 0.012 | -1.46 | 0.144 |
| **Often Vigorous Activity (0.0)** | 0.041 | 0.006 | 7.309 | 0.0 | -0.02 | 0.005 | -3.814 | 0.0 | -0.005 | 0.003 | -1.662 | 0.097 | 0.007 | 0.007 | 0.993 | 0.321 | -0.016 | 0.008 | -1.9 | 0.057 |
| **Often Vigorous Activity (1.0)** | -0.126 | 0.025 | -4.994 | 0.0 | 0.063 | 0.018 | 3.513 | 0.0 | 0.015 | 0.009 | 1.662 | 0.097 | -0.022 | 0.021 | -1.074 | 0.283 | 0.048 | 0.02 | 2.423 | 0.015 |
| **Often Moderate Activity (0.0)** | 0.067 | 0.016 | 4.183 | 0.0 | 0.021 | 0.01 | 2.067 | 0.039 | -0.018 | 0.013 | -1.4 | 0.162 | -0.016 | 0.016 | -0.997 | 0.319 | -0.047 | 0.01 | -4.766 | 0.0 |
| **Often Moderate Activity (1.0)** | -0.071 | 0.025 | -2.839 | 0.005 | -0.023 | 0.012 | -1.869 | 0.062 | 0.019 | 0.013 | 1.446 | 0.148 | 0.017 | 0.015 | 1.148 | 0.251 | 0.049 | 0.008 | 6.356 | 0.0 |
| **Often Mild Activity (0.0)** | 0.164 | 0.011 | 15.341 | 0.0 | 0.085 | 0.013 | 6.444 | 0.0 | 0.002 | 0.007 | 0.286 | 0.775 | -0.136 | 0.01 | -13.317 | 0.0 | -0.1 | 0.022 | -4.541 | 0.0 |
| **Often Mild Activity (1.0)** | -0.146 | 0.016 | -9.14 | 0.0 | -0.075 | 0.013 | -5.688 | 0.0 | -0.002 | 0.006 | -0.294 | 0.769 | 0.12 | 0.007 | 18.054 | 0.0 | 0.089 | 0.014 | 6.386 | 0.0 |
| **Drink Alcohol (0.0)** | 0.158 | 0.018 | 8.773 | 0.0 | 0.109 | 0.016 | 6.904 | 0.0 | -0.071 | 0.023 | -3.151 | 0.002 | -0.044 | 0.016 | -2.808 | 0.005 | -0.154 | 0.021 | -7.289 | 0.0 |
| **Drink Alcohol (1.0)** | -0.096 | 0.016 | -5.995 | 0.0 | -0.067 | 0.012 | -5.655 | 0.0 | 0.043 | 0.013 | 3.459 | 0.001 | 0.027 | 0.009 | 3.081 | 0.002 | 0.094 | 0.009 | 10.693 | 0.0 |
| **Education (0.0)** | 0.729 | 0.137 | 5.337 | 0.0 | 0.105 | 0.075 | 1.393 | 0.164 | -0.257 | 0.2 | -1.282 | 0.2 | -0.435 | 0.13 | -3.338 | 0.001 | -0.725 | 0.195 | -3.722 | 0.0 |
| **Education (0.5)** | 0.734 | 0.142 | 5.161 | 0.0 | 0.062 | 0.083 | 0.746 | 0.456 | -0.185 | 0.113 | -1.637 | 0.102 | -0.475 | 0.154 | -3.095 | 0.002 | -0.67 | 0.16 | -4.194 | 0.0 |
| **Education (1.5)** | 0.706 | 0.165 | 4.264 | 0.0 | 0.084 | 0.076 | 1.097 | 0.273 | -0.176 | 0.121 | -1.449 | 0.147 | -0.463 | 0.164 | -2.822 | 0.005 | -0.638 | 0.17 | -3.742 | 0.0 |
| **Education (2.5)** | 0.706 | 0.165 | 4.264 | 0.0 | 0.084 | 0.076 | 1.097 | 0.273 | -0.176 | 0.121 | -1.449 | 0.147 | -0.463 | 0.164 | -2.822 | 0.005 | -0.638 | 0.17 | -3.742 | 0.0 |
| **Education (4.5)** | 0.579 | 0.122 | 4.762 | 0.0 | 0.097 | 0.041 | 2.363 | 0.018 | -0.069 | 0.073 | -0.948 | 0.343 | -0.293 | 0.117 | -2.512 | 0.012 | -0.687 | 0.176 | -3.898 | 0.0 |
| **Education (5.5)** | 0.482 | 0.051 | 9.382 | 0.0 | 0.116 | 0.043 | 2.717 | 0.007 | -0.024 | 0.03 | -0.811 | 0.417 | -0.193 | 0.048 | -3.984 | 0.0 | -0.662 | 0.16 | -4.138 | 0.0 |
| **Education (6.5)** | 0.485 | 0.055 | 8.887 | 0.0 | 0.114 | 0.048 | 2.397 | 0.017 | -0.027 | 0.033 | -0.82 | 0.412 | -0.19 | 0.051 | -3.7 | 0.0 | -0.668 | 0.165 | -4.053 | 0.0 |
| **Education (7.5)** | 0.47 | 0.047 | 9.896 | 0.0 | 0.113 | 0.038 | 2.999 | 0.003 | -0.022 | 0.024 | -0.905 | 0.365 | -0.173 | 0.045 | -3.808 | 0.0 | -0.641 | 0.14 | -4.571 | 0.0 |
| **Education (8.5)** | 0.316 | 0.045 | 7.05 | 0.0 | 0.076 | 0.028 | 2.765 | 0.006 | 0.059 | 0.015 | 3.844 | 0.0 | -0.1 | 0.028 | -3.593 | 0.0 | -0.474 | 0.101 | -4.681 | 0.0 |
| **Education (9.5)** | 0.308 | 0.046 | 6.741 | 0.0 | 0.075 | 0.028 | 2.625 | 0.009 | 0.064 | 0.013 | 4.787 | 0.0 | -0.098 | 0.03 | -3.252 | 0.001 | -0.476 | 0.109 | -4.371 | 0.0 |
| **Education (10.5)** | 0.31 | 0.046 | 6.683 | 0.0 | 0.059 | 0.029 | 2.073 | 0.038 | 0.085 | 0.033 | 2.534 | 0.011 | -0.101 | 0.03 | -3.374 | 0.001 | -0.489 | 0.122 | -3.996 | 0.0 |
| **Education (11.5)** | 0.097 | 0.017 | 5.708 | 0.0 | 0.087 | 0.015 | 5.65 | 0.0 | 0.055 | 0.014 | 4.071 | 0.0 | -0.012 | 0.011 | -1.134 | 0.257 | -0.168 | 0.046 | -3.648 | 0.0 |
| **Education (12.5)** | -0.099 | 0.027 | -3.673 | 0.0 | 0.001 | 0.027 | 0.019 | 0.985 | -0.067 | 0.028 | -2.367 | 0.018 | 0.089 | 0.028 | 3.2 | 0.001 | 0.174 | 0.026 | 6.681 | 0.0 |
| **Education (13.5)** | -0.112 | 0.026 | -4.262 | 0.0 | 0.001 | 0.023 | 0.038 | 0.97 | -0.021 | 0.01 | -2.081 | 0.037 | 0.036 | 0.02 | 1.815 | 0.07 | 0.19 | 0.021 | 9.026 | 0.0 |
| **Education (14.5)** | -0.102 | 0.021 | -4.949 | 0.0 | -0.005 | 0.024 | -0.218 | 0.827 | -0.013 | 0.016 | -0.83 | 0.407 | 0.03 | 0.023 | 1.317 | 0.188 | 0.185 | 0.023 | 8.118 | 0.0 |
| **Education (15.5)** | -0.261 | 0.067 | -3.892 | 0.0 | -0.137 | 0.037 | -3.738 | 0.0 | -0.032 | 0.013 | -2.546 | 0.011 | 0.066 | 0.01 | 6.653 | 0.0 | 0.371 | 0.047 | 7.834 | 0.0 |
| **Education (16.5)** | -0.355 | 0.092 | -3.876 | 0.0 | -0.223 | 0.042 | -5.286 | 0.0 | -0.088 | 0.023 | -3.837 | 0.0 | 0.083 | 0.017 | 4.97 | 0.0 | 0.504 | 0.071 | 7.152 | 0.0 |
| **Education (17.0)** | -0.355 | 0.092 | -3.876 | 0.0 | -0.223 | 0.042 | -5.286 | 0.0 | -0.088 | 0.023 | -3.837 | 0.0 | 0.083 | 0.017 | 4.97 | 0.0 | 0.504 | 0.071 | 7.152 | 0.0 |
| **Smoking (0.0)** | -0.027 | 0.01 | -2.623 | 0.009 | -0.001 | 0.005 | -0.122 | 0.903 | 0.003 | 0.004 | 0.718 | 0.473 | 0.007 | 0.008 | 0.878 | 0.38 | 0.018 | 0.003 | 5.568 | 0.0 |
| **Smoking (1.0)** | 0.129 | 0.04 | 3.193 | 0.001 | 0.003 | 0.023 | 0.126 | 0.9 | -0.015 | 0.022 | -0.696 | 0.486 | -0.033 | 0.039 | -0.839 | 0.401 | -0.087 | 0.019 | -4.467 | 0.0 |
| **Age (60.0)** | -0.26 | 0.068 | -3.82 | 0.0 | -0.093 | 0.028 | -3.354 | 0.001 | 0.007 | 0.02 | 0.36 | 0.719 | 0.15 | 0.027 | 5.609 | 0.0 | 0.241 | 0.037 | 6.463 | 0.0 |
| **Age (60.5)** | -0.26 | 0.068 | -3.82 | 0.0 | -0.093 | 0.028 | -3.354 | 0.001 | 0.007 | 0.02 | 0.36 | 0.719 | 0.15 | 0.027 | 5.609 | 0.0 | 0.241 | 0.037 | 6.463 | 0.0 |
| **Age (61.5)** | -0.252 | 0.059 | -4.297 | 0.0 | -0.117 | 0.038 | -3.06 | 0.002 | 0.01 | 0.017 | 0.586 | 0.558 | 0.156 | 0.032 | 4.891 | 0.0 | 0.247 | 0.045 | 5.512 | 0.0 |
| **Age (62.5)** | -0.252 | 0.059 | -4.297 | 0.0 | -0.117 | 0.038 | -3.06 | 0.002 | 0.01 | 0.017 | 0.586 | 0.558 | 0.156 | 0.032 | 4.891 | 0.0 | 0.247 | 0.045 | 5.512 | 0.0 |
| **Age (63.5)** | -0.252 | 0.059 | -4.297 | 0.0 | -0.117 | 0.038 | -3.06 | 0.002 | 0.01 | 0.017 | 0.586 | 0.558 | 0.156 | 0.032 | 4.891 | 0.0 | 0.247 | 0.045 | 5.512 | 0.0 |
| **Age (64.5)** | -0.253 | 0.059 | -4.256 | 0.0 | -0.117 | 0.038 | -3.088 | 0.002 | 0.01 | 0.017 | 0.6 | 0.549 | 0.156 | 0.032 | 4.842 | 0.0 | 0.247 | 0.044 | 5.578 | 0.0 |
| **Age (65.5)** | -0.253 | 0.059 | -4.256 | 0.0 | -0.117 | 0.038 | -3.088 | 0.002 | 0.01 | 0.017 | 0.6 | 0.549 | 0.156 | 0.032 | 4.842 | 0.0 | 0.247 | 0.044 | 5.578 | 0.0 |
| **Age (66.5)** | -0.27 | 0.072 | -3.742 | 0.0 | -0.089 | 0.022 | -4.118 | 0.0 | 0.038 | 0.02 | 1.907 | 0.057 | 0.143 | 0.025 | 5.716 | 0.0 | 0.212 | 0.018 | 11.951 | 0.0 |
| **Age (67.5)** | -0.27 | 0.073 | -3.706 | 0.0 | -0.088 | 0.021 | -4.256 | 0.0 | 0.04 | 0.022 | 1.834 | 0.067 | 0.145 | 0.028 | 5.156 | 0.0 | 0.207 | 0.018 | 11.279 | 0.0 |
| **Age (68.5)** | -0.282 | 0.079 | -3.545 | 0.0 | -0.064 | 0.03 | -2.122 | 0.034 | 0.041 | 0.02 | 2.04 | 0.041 | 0.142 | 0.029 | 4.864 | 0.0 | 0.192 | 0.021 | 9.207 | 0.0 |
| **Age (69.5)** | -0.284 | 0.078 | -3.63 | 0.0 | -0.062 | 0.03 | -2.079 | 0.038 | 0.043 | 0.02 | 2.181 | 0.029 | 0.142 | 0.03 | 4.755 | 0.0 | 0.191 | 0.021 | 9.194 | 0.0 |
| **Age (70.5)** | -0.249 | 0.075 | -3.321 | 0.001 | -0.017 | 0.037 | -0.447 | 0.655 | 0.053 | 0.022 | 2.445 | 0.014 | 0.103 | 0.035 | 2.993 | 0.003 | 0.148 | 0.028 | 5.234 | 0.0 |
| **Age (71.5)** | -0.181 | 0.031 | -5.94 | 0.0 | 0.017 | 0.034 | 0.505 | 0.614 | 0.043 | 0.018 | 2.454 | 0.014 | 0.058 | 0.027 | 2.111 | 0.035 | 0.121 | 0.029 | 4.188 | 0.0 |
| **Age (72.5)** | -0.205 | 0.039 | -5.319 | 0.0 | 0.015 | 0.031 | 0.498 | 0.618 | 0.071 | 0.034 | 2.083 | 0.037 | 0.04 | 0.037 | 1.093 | 0.274 | 0.127 | 0.026 | 4.83 | 0.0 |
| **Age (73.5)** | -0.045 | 0.076 | -0.598 | 0.55 | 0.062 | 0.03 | 2.065 | 0.039 | 0.02 | 0.02 | 1.017 | 0.309 | 0.001 | 0.036 | 0.041 | 0.967 | 0.03 | 0.05 | 0.592 | 0.554 |
| **Age (74.5)** | 0.159 | 0.069 | 2.301 | 0.021 | 0.093 | 0.014 | 6.643 | 0.0 | -0.011 | 0.031 | -0.342 | 0.732 | -0.063 | 0.016 | -4.004 | 0.0 | -0.101 | 0.042 | -2.396 | 0.017 |
| **Age (75.5)** | 0.143 | 0.047 | 3.022 | 0.003 | 0.1 | 0.014 | 7.039 | 0.0 | 0.001 | 0.013 | 0.075 | 0.94 | -0.079 | 0.03 | -2.635 | 0.008 | -0.091 | 0.03 | -2.998 | 0.003 |
| **Age (76.5)** | 0.143 | 0.047 | 3.022 | 0.003 | 0.1 | 0.014 | 7.039 | 0.0 | 0.001 | 0.013 | 0.075 | 0.94 | -0.079 | 0.03 | -2.635 | 0.008 | -0.091 | 0.03 | -2.998 | 0.003 |
| **Age (77.5)** | 0.141 | 0.046 | 3.093 | 0.002 | 0.1 | 0.013 | 7.559 | 0.0 | -0.0 | 0.015 | -0.007 | 0.994 | -0.076 | 0.024 | -3.219 | 0.001 | -0.091 | 0.03 | -3.0 | 0.003 |
| **Age (78.5)** | 0.376 | 0.066 | 5.654 | 0.0 | 0.094 | 0.011 | 8.193 | 0.0 | 0.014 | 0.023 | 0.61 | 0.542 | -0.198 | 0.038 | -5.145 | 0.0 | -0.261 | 0.046 | -5.652 | 0.0 |
| **Age (79.5)** | 0.388 | 0.076 | 5.07 | 0.0 | 0.098 | 0.018 | 5.414 | 0.0 | 0.058 | 0.046 | 1.265 | 0.206 | -0.25 | 0.084 | -2.979 | 0.003 | -0.297 | 0.075 | -3.982 | 0.0 |
| **Age (80.5)** | 0.568 | 0.072 | 7.865 | 0.0 | 0.084 | 0.038 | 2.21 | 0.027 | 0.094 | 0.059 | 1.591 | 0.112 | -0.387 | 0.086 | -4.5 | 0.0 | -0.501 | 0.082 | -6.137 | 0.0 |
| **Age (81.5)** | 0.593 | 0.074 | 8.043 | 0.0 | 0.107 | 0.031 | 3.476 | 0.001 | -0.017 | 0.038 | -0.434 | 0.664 | -0.323 | 0.081 | -4.009 | 0.0 | -0.5 | 0.088 | -5.704 | 0.0 |
| **Age (82.5)** | 0.593 | 0.074 | 8.043 | 0.0 | 0.107 | 0.031 | 3.476 | 0.001 | -0.017 | 0.038 | -0.434 | 0.664 | -0.323 | 0.081 | -4.009 | 0.0 | -0.5 | 0.088 | -5.704 | 0.0 |
| **Age (83.5)** | 0.83 | 0.087 | 9.532 | 0.0 | 0.2 | 0.055 | 3.613 | 0.0 | -0.242 | 0.037 | -6.448 | 0.0 | -0.38 | 0.069 | -5.474 | 0.0 | -0.758 | 0.159 | -4.776 | 0.0 |
| **Age (84.5)** | 0.83 | 0.086 | 9.603 | 0.0 | 0.198 | 0.056 | 3.537 | 0.0 | -0.24 | 0.036 | -6.621 | 0.0 | -0.377 | 0.064 | -5.865 | 0.0 | -0.761 | 0.161 | -4.732 | 0.0 |
| **Age (85.5)** | 0.829 | 0.086 | 9.62 | 0.0 | 0.198 | 0.056 | 3.558 | 0.0 | -0.24 | 0.036 | -6.62 | 0.0 | -0.376 | 0.063 | -5.96 | 0.0 | -0.761 | 0.161 | -4.727 | 0.0 |
| **Age (86.5)** | 0.842 | 0.082 | 10.237 | 0.0 | 0.198 | 0.055 | 3.589 | 0.0 | -0.248 | 0.037 | -6.677 | 0.0 | -0.382 | 0.061 | -6.236 | 0.0 | -0.777 | 0.148 | -5.249 | 0.0 |
| **Age (87.5)** | 0.842 | 0.082 | 10.237 | 0.0 | 0.198 | 0.055 | 3.589 | 0.0 | -0.248 | 0.037 | -6.677 | 0.0 | -0.382 | 0.061 | -6.236 | 0.0 | -0.777 | 0.148 | -5.249 | 0.0 |
| **Age (88.5)** | 0.842 | 0.082 | 10.237 | 0.0 | 0.198 | 0.055 | 3.589 | 0.0 | -0.248 | 0.037 | -6.677 | 0.0 | -0.382 | 0.061 | -6.236 | 0.0 | -0.777 | 0.148 | -5.249 | 0.0 |
| **Age (89.5)** | 0.842 | 0.082 | 10.237 | 0.0 | 0.198 | 0.055 | 3.589 | 0.0 | -0.248 | 0.037 | -6.677 | 0.0 | -0.382 | 0.061 | -6.236 | 0.0 | -0.777 | 0.148 | -5.249 | 0.0 |
| **Age (90.5)** | 0.831 | 0.067 | 12.318 | 0.0 | 0.253 | 0.127 | 1.994 | 0.046 | -0.273 | 0.078 | -3.487 | 0.0 | -0.417 | 0.118 | -3.52 | 0.0 | -0.837 | 0.233 | -3.596 | 0.0 |
| **Age (91.5)** | 0.831 | 0.067 | 12.318 | 0.0 | 0.253 | 0.127 | 1.994 | 0.046 | -0.273 | 0.078 | -3.487 | 0.0 | -0.417 | 0.118 | -3.52 | 0.0 | -0.837 | 0.233 | -3.596 | 0.0 |
| **Age (92.5)** | 1.032 | 0.134 | 7.724 | 0.0 | 0.24 | 0.121 | 1.99 | 0.047 | -0.461 | 0.13 | -3.544 | 0.0 | -0.575 | 0.158 | -3.647 | 0.0 | -0.959 | 0.254 | -3.773 | 0.0 |
| **Age (93.5)** | 1.024 | 0.127 | 8.068 | 0.0 | 0.264 | 0.168 | 1.57 | 0.116 | -0.474 | 0.153 | -3.093 | 0.002 | -0.587 | 0.18 | -3.259 | 0.001 | -0.971 | 0.272 | -3.563 | 0.0 |
| **Age (94.5)** | 1.12 | 0.21 | 5.321 | 0.0 | 0.165 | 0.039 | 4.234 | 0.0 | -0.515 | 0.188 | -2.735 | 0.006 | -0.62 | 0.212 | -2.923 | 0.003 | -0.994 | 0.293 | -3.395 | 0.001 |
| **Age (96.0)** | 1.12 | 0.21 | 5.321 | 0.0 | 0.165 | 0.039 | 4.234 | 0.0 | -0.515 | 0.188 | -2.735 | 0.006 | -0.62 | 0.212 | -2.923 | 0.003 | -0.994 | 0.293 | -3.395 | 0.001 |
| **Age (99.0)** | 1.12 | 0.21 | 5.321 | 0.0 | 0.165 | 0.039 | 4.234 | 0.0 | -0.515 | 0.188 | -2.735 | 0.006 | -0.62 | 0.212 | -2.923 | 0.003 | -0.994 | 0.293 | -3.395 | 0.001 |

- 9 Cognitive Groups


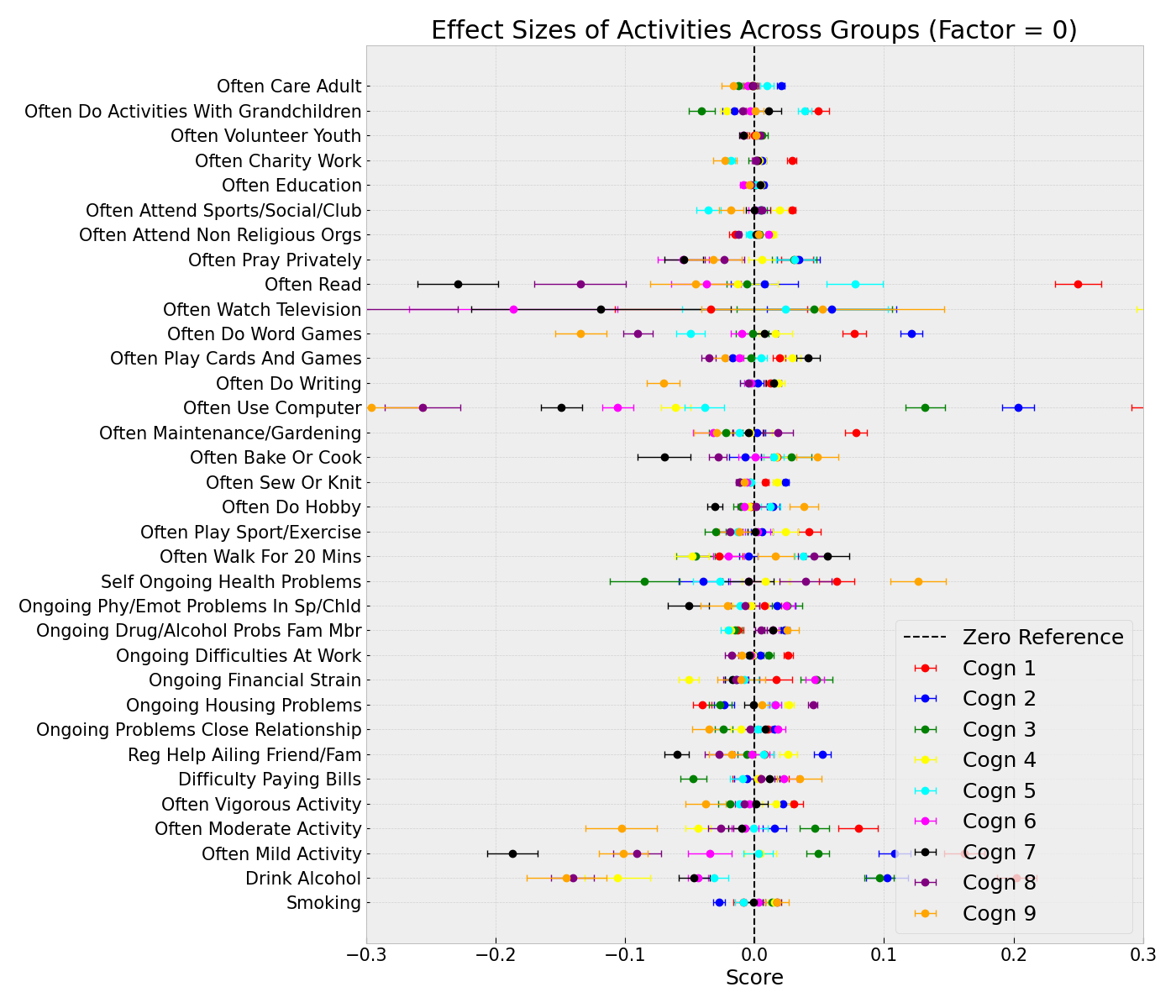


Supplementary Figure 9: Patterns obtained for all variables with the EBM model with factor 0 (not doing an activity) and 9 cognitive categories as dependent variable. The order of the variables is arbitrary.


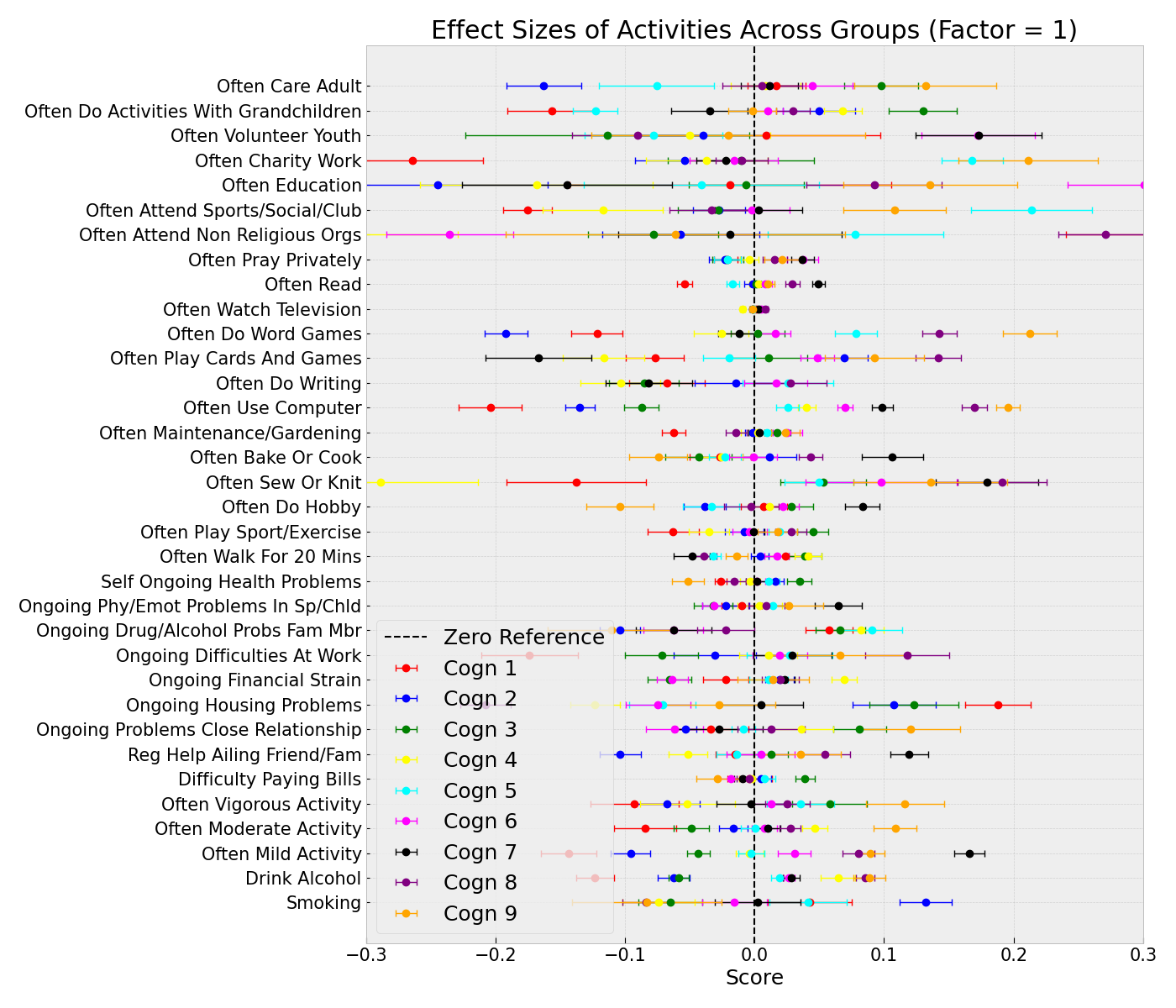


Supplementary Figure 10: Patterns obtained for all variables with the EBM model with factor 1 (doing an activity) and 9 cognitive categories as dependent variable. The order of the variables is arbitrary.


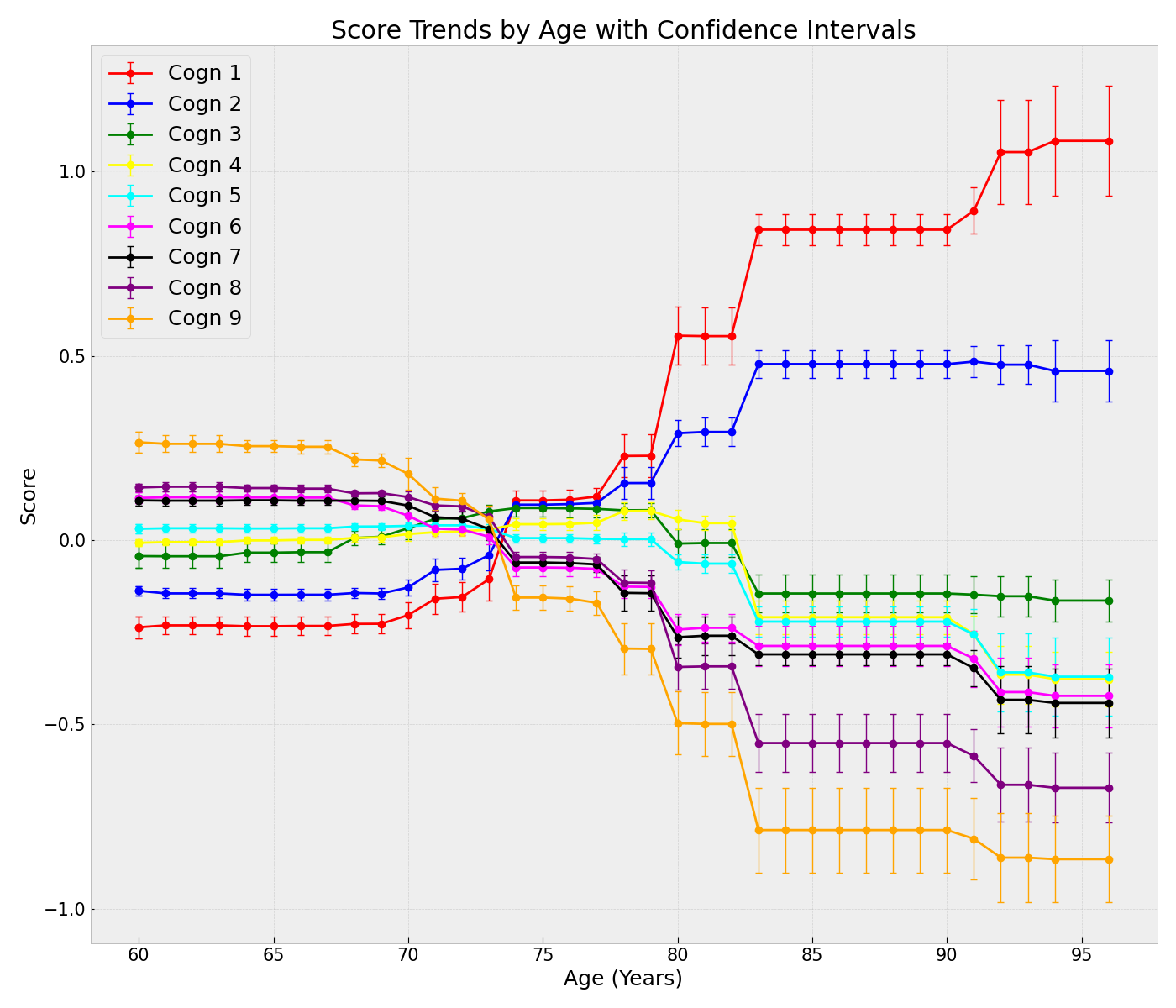


Supplementary Figure 11: Score trends by Age with confidence intervals of nine cognitive categories.


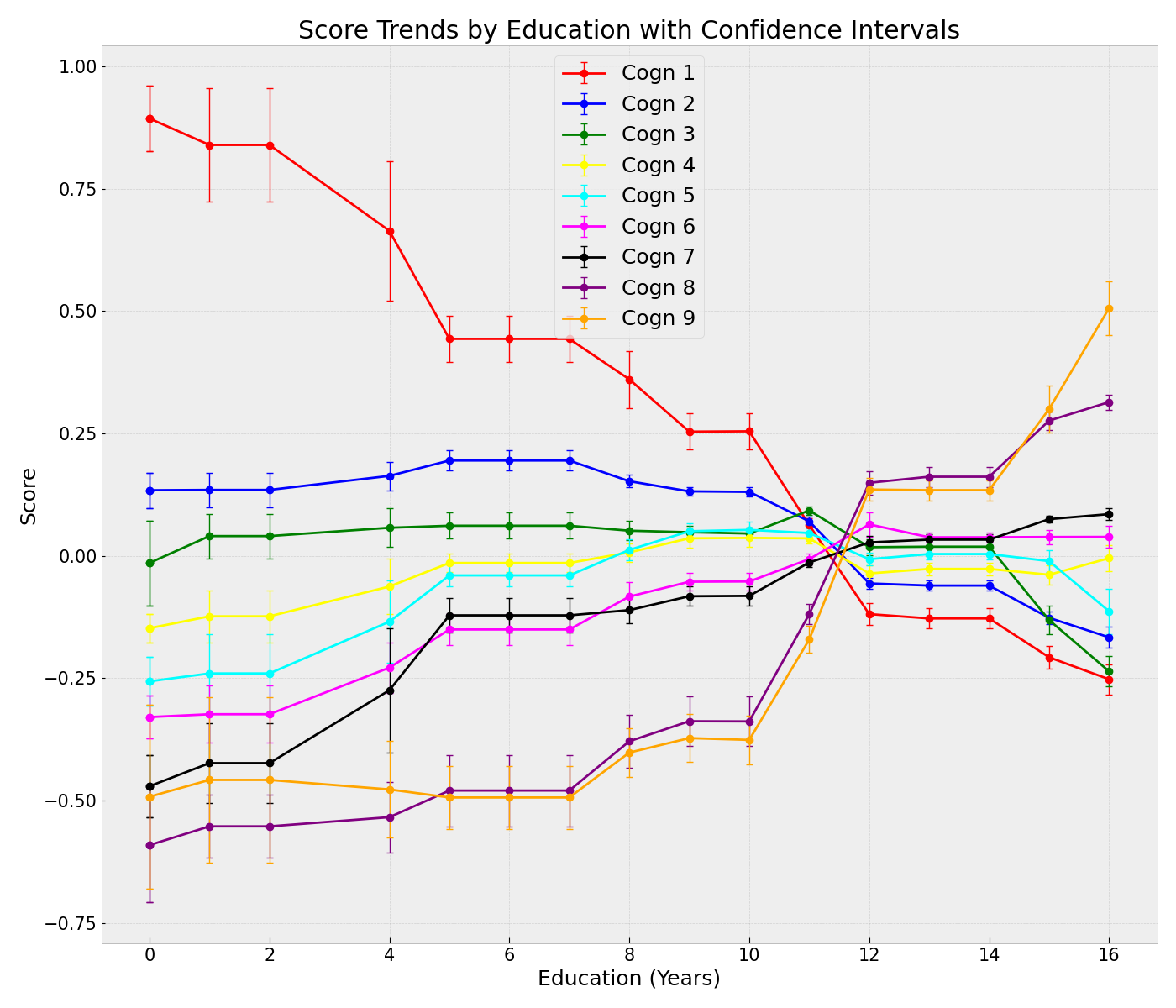


Supplementary Figure 12: Score trends by Age with confidence intervals of nine cognitive categories.

Supplementary Table 5: P-values of Wilcoxon signed rank tests results obtained for the dependent variable Cogn9

|  | EBM | XGB | LR | SVC | MLP | RF |
| --- | --- | --- | --- | --- | --- | --- |
| EBM | 1 | 0.062 | 0.625 | 1 | 0.062 | 0.062 |
| XGB | 0.062 | 1 | 0.062 | 0.062 | 0.062 | 0.062 |
| LR | 0.625 | 0.062 | 1 | 0.438 | 0.312 | 0.062 |
| SVC | 1 | 0.062 | 0.438 | 1 | 0.438 | 0.062 |
| MLP | 0.062 | 0.062 | 0.312 | 0.438 | 1 | 0.125 |
| RF | 0.062 | 0.062 | 0.062 | 0.062 | 0.125 | 1 |

Supplementary Table 6: Z-test scores obtained for each independent variable category. For Age and Education, the categories were automatedly determined by the EBM model.

|  | **Cogn 1** | | | | **Cogn 2** | | | | | **Cogn 3** | | | | | **Cogn 4** | | | | | **Cogn 5** | | | | | **Cogn 6** | | | | | **Cogn 7** | | | | | **Cogn 8** | | | | | **Cogn 9** | | | |
| --- | --- | --- | --- | --- | --- | --- | --- | --- | --- | --- | --- | --- | --- | --- | --- | --- | --- | --- | --- | --- | --- | --- | --- | --- | --- | --- | --- | --- | --- | --- | --- | --- | --- | --- | --- | --- | --- | --- | --- | --- | --- | --- | --- |
|  | **Coeff.** | **Std. Err.** | **z** | **P>\|z\|** | **Coeff.** | **Std. Err.** | **z** | **P>\|z\|** | **Coeff.** | | **Std. Err.** | **z** | **P>\|z\|** | **Coeff.** | | **Std. Err.** | **z** | **P>\|z\|** | **Coeff.** | | **Std. Err.** | **z** | **P>\|z\|** | **Coeff.** | | **Std. Err.** | **z** | **P>\|z\|** | **Coeff.** | | **Std. Err.** | **z** | **P>\|z\|** | **Coeff.** | | **Std. Err.** | **z** | **P>\|z\|** | **Coeff.** | | **Std. Err.** | **z** | **P>\|z\|** |
| **Often Care Adult (0.0)** | -0.002 | 0.003 | -0.81 | 0.418 | 0.02 | 0.003 | 6.847 | 0.0 | -0.012 | | 0.004 | -3.189 | 0.001 | -0.001 | | 0.003 | -0.32 | 0.749 | 0.01 | | 0.005 | 1.831 | 0.067 | -0.006 | | 0.004 | -1.379 | 0.168 | -0.001 | | 0.003 | -0.48 | 0.631 | -0.001 | | 0.004 | -0.182 | 0.856 | -0.017 | | 0.009 | -1.909 | 0.056 |
| **Often Care Adult (1.0)** | 0.017 | 0.022 | 0.751 | 0.453 | -0.163 | 0.029 | -5.64 | 0.0 | 0.098 | | 0.028 | 3.452 | 0.001 | 0.008 | | 0.027 | 0.314 | 0.754 | -0.076 | | 0.044 | -1.71 | 0.087 | 0.045 | | 0.031 | 1.442 | 0.149 | 0.012 | | 0.022 | 0.526 | 0.599 | 0.006 | | 0.031 | 0.191 | 0.849 | 0.132 | | 0.055 | 2.418 | 0.016 |
| **Often Do Activities With Grandchildren (0.0)** | 0.049 | 0.008 | 5.906 | 0.0 | -0.016 | 0.009 | -1.783 | 0.075 | -0.041 | | 0.01 | -4.088 | 0.0 | -0.021 | | 0.005 | -4.423 | 0.0 | 0.039 | | 0.005 | 7.399 | 0.0 | -0.003 | | 0.004 | -0.834 | 0.404 | 0.011 | | 0.009 | 1.153 | 0.249 | -0.009 | | 0.004 | -2.137 | 0.033 | 0.001 | | 0.006 | 0.086 | 0.931 |
| **Often Do Activities With Grandchildren (1.0)** | -0.157 | 0.034 | -4.543 | 0.0 | 0.05 | 0.028 | 1.789 | 0.074 | 0.13 | | 0.026 | 4.939 | 0.0 | 0.068 | | 0.015 | 4.702 | 0.0 | -0.123 | | 0.017 | -7.136 | 0.0 | 0.01 | | 0.012 | 0.885 | 0.376 | -0.035 | | 0.029 | -1.183 | 0.237 | 0.03 | | 0.013 | 2.331 | 0.02 | -0.002 | | 0.019 | -0.092 | 0.927 |
| **Often Volunteer Youth (0.0)** | -0.0 | 0.003 | -0.132 | 0.895 | 0.002 | 0.002 | 0.916 | 0.36 | 0.006 | | 0.005 | 1.257 | 0.209 | 0.003 | | 0.003 | 0.886 | 0.376 | 0.004 | | 0.002 | 1.571 | 0.116 | -0.009 | | 0.002 | -3.622 | 0.0 | -0.009 | | 0.003 | -2.83 | 0.005 | 0.005 | | 0.003 | 1.795 | 0.073 | 0.001 | | 0.006 | 0.157 | 0.875 |
| **Often Volunteer Youth (1.0)** | 0.009 | 0.088 | 0.102 | 0.919 | -0.04 | 0.05 | -0.797 | 0.425 | -0.114 | | 0.11 | -1.035 | 0.301 | -0.05 | | 0.062 | -0.802 | 0.423 | -0.078 | | 0.053 | -1.472 | 0.141 | 0.173 | | 0.044 | 3.931 | 0.0 | 0.173 | | 0.048 | 3.578 | 0.0 | -0.09 | | 0.051 | -1.777 | 0.076 | -0.02 | | 0.106 | -0.192 | 0.848 |
| **Often Charity Work (0.0)** | 0.029 | 0.003 | 8.341 | 0.0 | 0.006 | 0.003 | 1.867 | 0.062 | 0.001 | | 0.006 | 0.211 | 0.833 | 0.004 | | 0.005 | 0.854 | 0.393 | -0.018 | | 0.003 | -6.227 | 0.0 | 0.002 | | 0.004 | 0.449 | 0.653 | 0.002 | | 0.003 | 0.92 | 0.358 | 0.001 | | 0.002 | 0.438 | 0.661 | -0.023 | | 0.009 | -2.522 | 0.012 |
| **Often Charity Work (1.0)** | -0.264 | 0.054 | -4.852 | 0.0 | -0.054 | 0.038 | -1.423 | 0.155 | -0.011 | | 0.056 | -0.191 | 0.849 | -0.037 | | 0.047 | -0.785 | 0.432 | 0.168 | | 0.023 | 7.163 | 0.0 | -0.016 | | 0.034 | -0.467 | 0.64 | -0.022 | | 0.023 | -0.994 | 0.32 | -0.01 | | 0.02 | -0.496 | 0.62 | 0.211 | | 0.054 | 3.93 | 0.0 |
| **Often Education (0.0)** | 0.001 | 0.003 | 0.217 | 0.828 | 0.007 | 0.002 | 4.465 | 0.0 | 0.0 | | 0.001 | 0.153 | 0.878 | 0.005 | | 0.002 | 2.263 | 0.024 | 0.001 | | 0.003 | 0.481 | 0.631 | -0.009 | | 0.003 | -3.545 | 0.0 | 0.004 | | 0.002 | 1.865 | 0.062 | -0.003 | | 0.002 | -1.446 | 0.148 | -0.004 | | 0.003 | -1.369 | 0.171 |
| **Often Education (1.0)** | -0.019 | 0.124 | -0.151 | 0.88 | -0.245 | 0.085 | -2.867 | 0.004 | -0.006 | | 0.044 | -0.144 | 0.886 | -0.168 | | 0.09 | -1.875 | 0.061 | -0.041 | | 0.091 | -0.452 | 0.651 | 0.301 | | 0.059 | 5.081 | 0.0 | -0.145 | | 0.081 | -1.784 | 0.074 | 0.092 | | 0.052 | 1.773 | 0.076 | 0.136 | | 0.067 | 2.015 | 0.044 |
| **Often Attend Sports/Social/Club (0.0)** | 0.029 | 0.002 | 13.612 | 0.0 | 0.005 | 0.003 | 1.637 | 0.102 | 0.005 | | 0.005 | 0.997 | 0.319 | 0.02 | | 0.006 | 3.012 | 0.003 | -0.036 | | 0.009 | -3.838 | 0.0 | 0.0 | | 0.005 | 0.071 | 0.943 | -0.0 | | 0.006 | -0.08 | 0.936 | 0.006 | | 0.006 | 0.888 | 0.375 | -0.018 | | 0.009 | -1.936 | 0.053 |
| **Often Attend Sports/Social/Club (1.0)** | -0.175 | 0.019 | -9.427 | 0.0 | -0.027 | 0.02 | -1.368 | 0.171 | -0.028 | | 0.031 | -0.924 | 0.355 | -0.117 | | 0.046 | -2.521 | 0.012 | 0.214 | | 0.047 | 4.594 | 0.0 | -0.002 | | 0.029 | -0.077 | 0.939 | 0.003 | | 0.034 | 0.084 | 0.933 | -0.034 | | 0.032 | -1.046 | 0.296 | 0.108 | | 0.039 | 2.744 | 0.006 |
| **Often Attend Non Religious Orgs (0.0)** | -0.015 | 0.004 | -3.379 | 0.001 | 0.003 | 0.002 | 1.187 | 0.235 | 0.004 | | 0.002 | 1.74 | 0.082 | 0.014 | | 0.002 | 5.96 | 0.0 | -0.004 | | 0.003 | -1.077 | 0.281 | 0.011 | | 0.002 | 5.883 | 0.0 | 0.001 | | 0.004 | 0.219 | 0.827 | -0.012 | | 0.002 | -6.104 | 0.0 | 0.003 | | 0.006 | 0.44 | 0.66 |
| **Often Attend Non Religious Orgs (1.0)** | 0.335 | 0.094 | 3.557 | 0.0 | -0.057 | 0.061 | -0.943 | 0.346 | -0.078 | | 0.05 | -1.557 | 0.119 | -0.312 | | 0.083 | -3.768 | 0.0 | 0.078 | | 0.068 | 1.148 | 0.251 | -0.235 | | 0.049 | -4.803 | 0.0 | -0.019 | | 0.086 | -0.223 | 0.824 | 0.271 | | 0.036 | 7.494 | 0.0 | -0.061 | | 0.131 | -0.468 | 0.64 |
| **Often Pray Privately (0.0)** | 0.033 | 0.012 | 2.698 | 0.007 | 0.034 | 0.017 | 2.012 | 0.044 | 0.031 | | 0.017 | 1.77 | 0.077 | 0.006 | | 0.01 | 0.569 | 0.569 | 0.031 | | 0.015 | 2.103 | 0.035 | -0.055 | | 0.02 | -2.796 | 0.005 | -0.055 | | 0.015 | -3.696 | 0.0 | -0.023 | | 0.015 | -1.547 | 0.122 | -0.032 | | 0.022 | -1.465 | 0.143 |
| **Often Pray Privately (1.0)** | -0.022 | 0.009 | -2.427 | 0.015 | -0.023 | 0.012 | -1.885 | 0.059 | -0.021 | | 0.012 | -1.749 | 0.08 | -0.004 | | 0.007 | -0.552 | 0.581 | -0.021 | | 0.01 | -2.067 | 0.039 | 0.037 | | 0.012 | 3.128 | 0.002 | 0.037 | | 0.009 | 4.201 | 0.0 | 0.016 | | 0.01 | 1.618 | 0.106 | 0.022 | | 0.014 | 1.58 | 0.114 |
| **Often Read (0.0)** | 0.25 | 0.018 | 13.884 | 0.0 | 0.008 | 0.026 | 0.289 | 0.773 | -0.006 | | 0.016 | -0.386 | 0.699 | -0.013 | | 0.032 | -0.421 | 0.674 | 0.078 | | 0.022 | 3.57 | 0.0 | -0.037 | | 0.027 | -1.381 | 0.167 | -0.229 | | 0.031 | -7.368 | 0.0 | -0.135 | | 0.036 | -3.79 | 0.0 | -0.046 | | 0.035 | -1.312 | 0.19 |
| **Often Read (1.0)** | -0.054 | 0.006 | -9.319 | 0.0 | -0.002 | 0.007 | -0.249 | 0.803 | 0.001 | | 0.003 | 0.375 | 0.708 | 0.003 | | 0.007 | 0.433 | 0.665 | -0.017 | | 0.005 | -3.371 | 0.001 | 0.008 | | 0.005 | 1.599 | 0.11 | 0.049 | | 0.005 | 10.21 | 0.0 | 0.029 | | 0.005 | 5.284 | 0.0 | 0.01 | | 0.005 | 1.801 | 0.072 |
| **Often Watch Television (0.0)** | -0.034 | 0.074 | -0.454 | 0.65 | 0.06 | 0.05 | 1.191 | 0.234 | 0.046 | | 0.06 | 0.767 | 0.443 | 0.353 | | 0.058 | 6.072 | 0.0 | 0.024 | | 0.079 | 0.297 | 0.766 | -0.186 | | 0.08 | -2.316 | 0.021 | -0.119 | | 0.1 | -1.186 | 0.236 | -0.327 | | 0.098 | -3.345 | 0.001 | 0.053 | | 0.094 | 0.563 | 0.573 |
| **Often Watch Television (1.0)** | 0.001 | 0.002 | 0.47 | 0.638 | -0.002 | 0.001 | -1.17 | 0.242 | -0.001 | | 0.002 | -0.782 | 0.434 | -0.009 | | 0.002 | -5.046 | 0.0 | -0.001 | | 0.002 | -0.283 | 0.777 | 0.005 | | 0.002 | 2.616 | 0.009 | 0.003 | | 0.002 | 1.338 | 0.181 | 0.008 | | 0.002 | 4.456 | 0.0 | -0.001 | | 0.003 | -0.541 | 0.589 |
| **Often Do Word Games (0.0)** | 0.077 | 0.009 | 8.353 | 0.0 | 0.121 | 0.008 | 14.34 | 0.0 | -0.002 | | 0.013 | -0.121 | 0.904 | 0.016 | | 0.013 | 1.279 | 0.201 | -0.049 | | 0.011 | -4.446 | 0.0 | -0.01 | | 0.008 | -1.261 | 0.207 | 0.008 | | 0.01 | 0.738 | 0.461 | -0.09 | | 0.012 | -7.802 | 0.0 | -0.134 | | 0.02 | -6.785 | 0.0 |
| **Often Do Word Games (1.0)** | -0.122 | 0.02 | -6.157 | 0.0 | -0.192 | 0.016 | -11.674 | 0.0 | 0.002 | | 0.021 | 0.118 | 0.906 | -0.026 | | 0.021 | -1.214 | 0.225 | 0.078 | | 0.016 | 4.823 | 0.0 | 0.016 | | 0.011 | 1.398 | 0.162 | -0.012 | | 0.016 | -0.757 | 0.449 | 0.143 | | 0.013 | 10.698 | 0.0 | 0.213 | | 0.021 | 10.141 | 0.0 |
| **Often Play Cards And Games (0.0)** | 0.019 | 0.005 | 4.001 | 0.0 | -0.017 | 0.005 | -3.768 | 0.0 | -0.003 | | 0.007 | -0.385 | 0.7 | 0.029 | | 0.007 | 4.318 | 0.0 | 0.005 | | 0.005 | 0.985 | 0.325 | -0.012 | | 0.004 | -3.341 | 0.001 | 0.041 | | 0.009 | 4.534 | 0.0 | -0.035 | | 0.006 | -6.319 | 0.0 | -0.023 | | 0.011 | -2.03 | 0.042 |
| **Often Play Cards And Games (1.0)** | -0.077 | 0.023 | -3.407 | 0.001 | 0.069 | 0.019 | 3.694 | 0.0 | 0.011 | | 0.029 | 0.381 | 0.703 | -0.117 | | 0.031 | -3.729 | 0.0 | -0.02 | | 0.02 | -0.972 | 0.331 | 0.049 | | 0.013 | 3.742 | 0.0 | -0.167 | | 0.041 | -4.106 | 0.0 | 0.142 | | 0.017 | 8.143 | 0.0 | 0.093 | | 0.038 | 2.42 | 0.016 |
| **Often Do Writing (0.0)** | 0.012 | 0.004 | 3.267 | 0.001 | 0.003 | 0.005 | 0.546 | 0.585 | 0.015 | | 0.004 | 3.725 | 0.0 | 0.019 | | 0.005 | 3.983 | 0.0 | -0.005 | | 0.006 | -0.723 | 0.47 | -0.003 | | 0.005 | -0.624 | 0.533 | 0.015 | | 0.006 | 2.43 | 0.015 | -0.005 | | 0.006 | -0.799 | 0.424 | -0.07 | | 0.013 | -5.61 | 0.0 |
| **Often Do Writing (1.0)** | -0.068 | 0.029 | -2.296 | 0.022 | -0.014 | 0.032 | -0.447 | 0.655 | -0.085 | | 0.027 | -3.151 | 0.002 | -0.103 | | 0.031 | -3.343 | 0.001 | 0.026 | | 0.035 | 0.73 | 0.465 | 0.017 | | 0.024 | 0.68 | 0.497 | -0.082 | | 0.033 | -2.457 | 0.014 | 0.028 | | 0.028 | 0.986 | 0.324 | 0.391 | | 0.033 | 11.691 | 0.0 |
| **Often Use Computer (0.0)** | 0.308 | 0.017 | 18.191 | 0.0 | 0.204 | 0.012 | 16.628 | 0.0 | 0.132 | | 0.015 | 8.551 | 0.0 | -0.061 | | 0.011 | -5.462 | 0.0 | -0.039 | | 0.015 | -2.534 | 0.011 | -0.106 | | 0.012 | -8.657 | 0.0 | -0.149 | | 0.016 | -9.294 | 0.0 | -0.256 | | 0.029 | -8.836 | 0.0 | -0.296 | | 0.041 | -7.189 | 0.0 |
| **Often Use Computer (1.0)** | -0.204 | 0.024 | -8.42 | 0.0 | -0.135 | 0.011 | -11.873 | 0.0 | -0.087 | | 0.013 | -6.499 | 0.0 | 0.04 | | 0.007 | 5.943 | 0.0 | 0.026 | | 0.009 | 2.895 | 0.004 | 0.07 | | 0.006 | 12.141 | 0.0 | 0.099 | | 0.008 | 12.593 | 0.0 | 0.17 | | 0.009 | 17.888 | 0.0 | 0.196 | | 0.009 | 21.728 | 0.0 |
| **Often Maintenance/Gardening (0.0)** | 0.078 | 0.008 | 9.267 | 0.0 | 0.002 | 0.007 | 0.238 | 0.812 | -0.022 | | 0.013 | -1.706 | 0.088 | -0.003 | | 0.017 | -0.194 | 0.846 | -0.012 | | 0.008 | -1.391 | 0.164 | -0.032 | | 0.016 | -2.036 | 0.042 | -0.005 | | 0.012 | -0.408 | 0.683 | 0.018 | | 0.012 | 1.553 | 0.12 | -0.03 | | 0.017 | -1.715 | 0.086 |
| **Often Maintenance/Gardening (1.0)** | -0.062 | 0.009 | -6.928 | 0.0 | -0.001 | 0.006 | -0.207 | 0.836 | 0.018 | | 0.01 | 1.761 | 0.078 | 0.003 | | 0.013 | 0.193 | 0.847 | 0.009 | | 0.007 | 1.404 | 0.16 | 0.025 | | 0.011 | 2.282 | 0.022 | 0.004 | | 0.009 | 0.461 | 0.645 | -0.014 | | 0.008 | -1.751 | 0.08 | 0.024 | | 0.011 | 2.163 | 0.031 |
| **Often Bake Or Cook (0.0)** | 0.017 | 0.012 | 1.412 | 0.158 | -0.007 | 0.012 | -0.601 | 0.548 | 0.028 | | 0.016 | 1.793 | 0.073 | 0.017 | | 0.01 | 1.672 | 0.095 | 0.015 | | 0.008 | 1.913 | 0.056 | 0.001 | | 0.013 | 0.05 | 0.96 | -0.07 | | 0.02 | -3.437 | 0.001 | -0.028 | | 0.007 | -4.094 | 0.0 | 0.049 | | 0.016 | 2.999 | 0.003 |
| **Often Bake Or Cook (1.0)** | -0.026 | 0.024 | -1.114 | 0.265 | 0.011 | 0.021 | 0.545 | 0.586 | -0.043 | | 0.026 | -1.675 | 0.094 | -0.025 | | 0.016 | -1.606 | 0.108 | -0.023 | | 0.012 | -1.855 | 0.064 | -0.001 | | 0.018 | -0.054 | 0.957 | 0.106 | | 0.024 | 4.48 | 0.0 | 0.043 | | 0.009 | 4.914 | 0.0 | -0.074 | | 0.022 | -3.311 | 0.001 |
| **Often Sew Or Knit (0.0)** | 0.008 | 0.002 | 3.611 | 0.0 | 0.024 | 0.002 | 11.57 | 0.0 | -0.003 | | 0.002 | -1.565 | 0.118 | 0.017 | | 0.003 | 5.461 | 0.0 | -0.003 | | 0.002 | -1.834 | 0.067 | -0.006 | | 0.004 | -1.444 | 0.149 | -0.011 | | 0.003 | -3.617 | 0.0 | -0.012 | | 0.003 | -4.023 | 0.0 | -0.008 | | 0.005 | -1.784 | 0.074 |
| **Often Sew Or Knit (1.0)** | -0.137 | 0.054 | -2.555 | 0.011 | -0.398 | 0.053 | -7.502 | 0.0 | 0.053 | | 0.033 | 1.602 | 0.109 | -0.289 | | 0.075 | -3.862 | 0.0 | 0.05 | | 0.027 | 1.852 | 0.064 | 0.098 | | 0.058 | 1.68 | 0.093 | 0.18 | | 0.04 | 4.54 | 0.0 | 0.191 | | 0.034 | 5.554 | 0.0 | 0.136 | | 0.059 | 2.294 | 0.022 |
| **Often Do Hobby (0.0)** | -0.003 | 0.005 | -0.545 | 0.586 | 0.014 | 0.005 | 2.906 | 0.004 | -0.01 | | 0.006 | -1.671 | 0.095 | -0.004 | | 0.006 | -0.714 | 0.475 | 0.012 | | 0.007 | 1.652 | 0.099 | -0.008 | | 0.005 | -1.602 | 0.109 | -0.031 | | 0.006 | -5.149 | 0.0 | 0.001 | | 0.009 | 0.107 | 0.915 | 0.038 | | 0.011 | 3.478 | 0.001 |
| **Often Do Hobby (1.0)** | 0.007 | 0.018 | 0.398 | 0.691 | -0.038 | 0.016 | -2.343 | 0.019 | 0.028 | | 0.017 | 1.649 | 0.099 | 0.012 | | 0.016 | 0.708 | 0.479 | -0.034 | | 0.021 | -1.561 | 0.119 | 0.022 | | 0.012 | 1.796 | 0.072 | 0.083 | | 0.013 | 6.241 | 0.0 | -0.003 | | 0.021 | -0.128 | 0.898 | -0.104 | | 0.026 | -3.99 | 0.0 |
| **Often Play Sport/Exercise (0.0)** | 0.042 | 0.009 | 4.807 | 0.0 | 0.005 | 0.008 | 0.646 | 0.518 | -0.03 | | 0.008 | -3.731 | 0.0 | 0.024 | | 0.01 | 2.457 | 0.014 | -0.013 | | 0.006 | -2.245 | 0.025 | 0.003 | | 0.01 | 0.263 | 0.793 | 0.001 | | 0.006 | 0.105 | 0.916 | -0.019 | | 0.01 | -1.958 | 0.05 | -0.012 | | 0.016 | -0.771 | 0.441 |
| **Often Play Sport/Exercise (1.0)** | -0.063 | 0.02 | -3.172 | 0.002 | -0.008 | 0.015 | -0.543 | 0.587 | 0.045 | | 0.012 | 3.797 | 0.0 | -0.035 | | 0.015 | -2.281 | 0.023 | 0.02 | | 0.008 | 2.32 | 0.02 | -0.004 | | 0.013 | -0.286 | 0.775 | -0.001 | | 0.008 | -0.114 | 0.909 | 0.028 | | 0.012 | 2.368 | 0.018 | 0.018 | | 0.015 | 1.171 | 0.242 |
| **Often Walk For 20 Mins (0.0)** | -0.027 | 0.018 | -1.504 | 0.133 | -0.005 | 0.007 | -0.667 | 0.505 | -0.045 | | 0.015 | -3.021 | 0.003 | -0.048 | | 0.013 | -3.741 | 0.0 | 0.038 | | 0.006 | 5.856 | 0.0 | -0.02 | | 0.012 | -1.638 | 0.101 | 0.056 | | 0.017 | 3.368 | 0.001 | 0.046 | | 0.012 | 3.805 | 0.0 | 0.016 | | 0.014 | 1.16 | 0.246 |
| **Often Walk For 20 Mins (1.0)** | 0.024 | 0.019 | 1.207 | 0.227 | 0.004 | 0.007 | 0.597 | 0.551 | 0.039 | | 0.012 | 3.139 | 0.002 | 0.041 | | 0.01 | 3.938 | 0.0 | -0.032 | | 0.006 | -5.445 | 0.0 | 0.017 | | 0.01 | 1.795 | 0.073 | -0.048 | | 0.014 | -3.46 | 0.001 | -0.039 | | 0.01 | -4.017 | 0.0 | -0.014 | | 0.008 | -1.653 | 0.098 |
| **Self Ongoing Health Problems (0.0)** | 0.063 | 0.014 | 4.649 | 0.0 | -0.039 | 0.019 | -2.067 | 0.039 | -0.085 | | 0.027 | -3.151 | 0.002 | 0.009 | | 0.019 | 0.463 | 0.643 | -0.027 | | 0.021 | -1.255 | 0.209 | -0.005 | | 0.014 | -0.338 | 0.735 | -0.005 | | 0.02 | -0.255 | 0.799 | 0.039 | | 0.02 | 1.941 | 0.052 | 0.126 | | 0.022 | 5.864 | 0.0 |
| **Self Ongoing Health Problems (1.0)** | -0.026 | 0.005 | -5.656 | 0.0 | 0.016 | 0.007 | 2.412 | 0.016 | 0.035 | | 0.009 | 3.682 | 0.0 | -0.004 | | 0.008 | -0.46 | 0.646 | 0.011 | | 0.008 | 1.294 | 0.196 | 0.002 | | 0.006 | 0.32 | 0.749 | 0.002 | | 0.008 | 0.244 | 0.807 | -0.016 | | 0.009 | -1.728 | 0.084 | -0.052 | | 0.012 | -4.184 | 0.0 |
| **Ongoing Phy/Emot Problems In Sp/Chld (0.0)** | 0.008 | 0.013 | 0.624 | 0.533 | 0.018 | 0.014 | 1.277 | 0.202 | 0.025 | | 0.012 | 2.178 | 0.029 | -0.003 | | 0.014 | -0.22 | 0.826 | -0.011 | | 0.01 | -1.18 | 0.238 | 0.025 | | 0.007 | 3.779 | 0.0 | -0.051 | | 0.016 | -3.188 | 0.001 | -0.007 | | 0.011 | -0.662 | 0.508 | -0.021 | | 0.021 | -1.006 | 0.314 |
| **Ongoing Phy/Emot Problems In Sp/Chld (1.0)** | -0.01 | 0.015 | -0.648 | 0.517 | -0.022 | 0.018 | -1.245 | 0.213 | -0.032 | | 0.015 | -2.18 | 0.029 | 0.004 | | 0.017 | 0.223 | 0.824 | 0.014 | | 0.012 | 1.194 | 0.232 | -0.031 | | 0.009 | -3.523 | 0.0 | 0.065 | | 0.018 | 3.54 | 0.0 | 0.009 | | 0.014 | 0.648 | 0.517 | 0.026 | | 0.027 | 0.991 | 0.322 |
| **Ongoing Drug/Alcohol Probs Fam Mbr (0.0)** | -0.013 | 0.004 | -2.995 | 0.003 | 0.023 | 0.003 | 7.007 | 0.0 | -0.015 | | 0.005 | -3.151 | 0.002 | -0.019 | | 0.004 | -4.155 | 0.0 | -0.02 | | 0.006 | -3.435 | 0.001 | 0.014 | | 0.005 | 3.037 | 0.002 | 0.014 | | 0.006 | 2.217 | 0.027 | 0.005 | | 0.005 | 1.049 | 0.294 | 0.025 | | 0.009 | 2.672 | 0.008 |
| **Ongoing Drug/Alcohol Probs Fam Mbr (1.0)** | 0.058 | 0.018 | 3.197 | 0.001 | -0.104 | 0.016 | -6.663 | 0.0 | 0.066 | | 0.019 | 3.494 | 0.0 | 0.082 | | 0.017 | 4.749 | 0.0 | 0.09 | | 0.024 | 3.839 | 0.0 | -0.063 | | 0.023 | -2.762 | 0.006 | -0.063 | | 0.029 | -2.13 | 0.033 | -0.022 | | 0.022 | -1.019 | 0.308 | -0.111 | | 0.049 | -2.248 | 0.025 |
| **Ongoing Difficulties At Work (0.0)** | 0.026 | 0.004 | 6.872 | 0.0 | 0.005 | 0.004 | 1.032 | 0.302 | 0.011 | | 0.004 | 2.75 | 0.006 | -0.002 | | 0.003 | -0.471 | 0.638 | -0.004 | | 0.005 | -0.802 | 0.423 | -0.003 | | 0.003 | -0.923 | 0.356 | -0.004 | | 0.005 | -0.915 | 0.36 | -0.018 | | 0.005 | -3.347 | 0.001 | -0.01 | | 0.009 | -1.07 | 0.285 |
| **Ongoing Difficulties At Work (1.0)** | -0.174 | 0.037 | -4.641 | 0.0 | -0.031 | 0.032 | -0.956 | 0.339 | -0.072 | | 0.028 | -2.521 | 0.012 | 0.011 | | 0.022 | 0.478 | 0.633 | 0.027 | | 0.033 | 0.825 | 0.409 | 0.019 | | 0.021 | 0.921 | 0.357 | 0.029 | | 0.03 | 0.963 | 0.336 | 0.118 | | 0.032 | 3.651 | 0.0 | 0.066 | | 0.054 | 1.225 | 0.221 |
| **Ongoing Financial Strain (0.0)** | 0.016 | 0.013 | 1.274 | 0.203 | -0.014 | 0.009 | -1.609 | 0.108 | 0.048 | | 0.012 | 3.865 | 0.0 | -0.051 | | 0.008 | -6.428 | 0.0 | -0.008 | | 0.011 | -0.725 | 0.468 | 0.046 | | 0.007 | 6.459 | 0.0 | -0.017 | | 0.006 | -2.948 | 0.003 | -0.014 | | 0.01 | -1.47 | 0.142 | -0.011 | | 0.018 | -0.57 | 0.569 |
| **Ongoing Financial Strain (1.0)** | -0.022 | 0.017 | -1.29 | 0.197 | 0.019 | 0.011 | 1.703 | 0.089 | -0.066 | | 0.017 | -3.951 | 0.0 | 0.069 | | 0.01 | 7.077 | 0.0 | 0.011 | | 0.015 | 0.735 | 0.462 | -0.064 | | 0.012 | -5.298 | 0.0 | 0.023 | | 0.007 | 3.096 | 0.002 | 0.02 | | 0.014 | 1.387 | 0.165 | 0.014 | | 0.028 | 0.52 | 0.603 |
| **Ongoing Housing Problems (0.0)** | -0.041 | 0.007 | -5.638 | 0.0 | -0.023 | 0.008 | -2.969 | 0.003 | -0.027 | | 0.009 | -3.059 | 0.002 | 0.027 | | 0.004 | 7.061 | 0.0 | 0.015 | | 0.005 | 2.944 | 0.003 | 0.016 | | 0.005 | 3.513 | 0.0 | -0.001 | | 0.007 | -0.154 | 0.878 | 0.045 | | 0.003 | 13.593 | 0.0 | 0.006 | | 0.008 | 0.762 | 0.446 |
| **Ongoing Housing Problems (1.0)** | 0.188 | 0.025 | 7.44 | 0.0 | 0.108 | 0.032 | 3.354 | 0.001 | 0.123 | | 0.034 | 3.588 | 0.0 | -0.123 | | 0.019 | -6.441 | 0.0 | -0.071 | | 0.026 | -2.761 | 0.006 | -0.075 | | 0.025 | -2.976 | 0.003 | 0.005 | | 0.032 | 0.15 | 0.881 | -0.208 | | 0.02 | -10.588 | 0.0 | -0.027 | | 0.044 | -0.631 | 0.528 |
| **Ongoing Problems Close Relationship (0.0)** | 0.01 | 0.008 | 1.245 | 0.213 | 0.016 | 0.002 | 6.698 | 0.0 | -0.024 | | 0.007 | -3.608 | 0.0 | -0.011 | | 0.007 | -1.432 | 0.152 | 0.003 | | 0.003 | 0.97 | 0.332 | 0.018 | | 0.006 | 3.276 | 0.001 | 0.008 | | 0.01 | 0.799 | 0.424 | -0.004 | | 0.008 | -0.478 | 0.633 | -0.035 | | 0.013 | -2.748 | 0.006 |
| **Ongoing Problems Close Relationship (1.0)** | -0.034 | 0.029 | -1.169 | 0.242 | -0.053 | 0.008 | -6.426 | 0.0 | 0.081 | | 0.021 | 3.948 | 0.0 | 0.036 | | 0.024 | 1.474 | 0.14 | -0.009 | | 0.009 | -0.958 | 0.338 | -0.062 | | 0.022 | -2.824 | 0.005 | -0.027 | | 0.033 | -0.817 | 0.414 | 0.013 | | 0.026 | 0.483 | 0.629 | 0.121 | | 0.038 | 3.159 | 0.002 |
| **Reg Help Ailing Friend/Fam (0.0)** | 0.008 | 0.007 | 1.102 | 0.27 | 0.052 | 0.007 | 8.022 | 0.0 | -0.006 | | 0.007 | -0.908 | 0.364 | 0.026 | | 0.007 | 3.764 | 0.0 | 0.007 | | 0.008 | 0.88 | 0.379 | -0.002 | | 0.014 | -0.178 | 0.859 | -0.06 | | 0.009 | -6.6 | 0.0 | -0.027 | | 0.011 | -2.403 | 0.016 | -0.018 | | 0.017 | -1.04 | 0.298 |
| **Reg Help Ailing Friend/Fam (1.0)** | -0.015 | 0.015 | -1.016 | 0.31 | -0.104 | 0.016 | -6.464 | 0.0 | 0.013 | | 0.013 | 0.939 | 0.348 | -0.051 | | 0.015 | -3.501 | 0.0 | -0.014 | | 0.016 | -0.86 | 0.39 | 0.005 | | 0.026 | 0.183 | 0.855 | 0.119 | | 0.015 | 8.115 | 0.0 | 0.054 | | 0.02 | 2.693 | 0.007 | 0.035 | | 0.031 | 1.132 | 0.258 |
| **Difficulty Paying Bills (0.0)** | 0.005 | 0.022 | 0.209 | 0.834 | -0.006 | 0.01 | -0.557 | 0.578 | -0.047 | | 0.01 | -4.646 | 0.0 | 0.003 | | 0.009 | 0.283 | 0.777 | -0.009 | | 0.01 | -0.877 | 0.38 | 0.022 | | 0.011 | 2.085 | 0.037 | 0.011 | | 0.008 | 1.458 | 0.145 | 0.005 | | 0.01 | 0.512 | 0.609 | 0.035 | | 0.017 | 2.022 | 0.043 |
| **Difficulty Paying Bills (1.0)** | -0.004 | 0.017 | -0.218 | 0.827 | 0.005 | 0.008 | 0.59 | 0.555 | 0.039 | | 0.008 | 5.05 | 0.0 | -0.002 | | 0.007 | -0.287 | 0.774 | 0.007 | | 0.009 | 0.866 | 0.386 | -0.018 | | 0.01 | -1.89 | 0.059 | -0.009 | | 0.007 | -1.435 | 0.151 | -0.004 | | 0.009 | -0.468 | 0.64 | -0.029 | | 0.017 | -1.73 | 0.084 |
| **Often Vigorous Activity (0.0)** | 0.03 | 0.007 | 4.269 | 0.0 | 0.022 | 0.006 | 3.431 | 0.001 | -0.019 | | 0.009 | -2.072 | 0.038 | 0.017 | | 0.011 | 1.531 | 0.126 | -0.012 | | 0.009 | -1.293 | 0.196 | -0.004 | | 0.005 | -0.832 | 0.405 | 0.001 | | 0.01 | 0.094 | 0.925 | -0.008 | | 0.007 | -1.172 | 0.241 | -0.038 | | 0.016 | -2.394 | 0.017 |
| **Often Vigorous Activity (1.0)** | -0.093 | 0.034 | -2.73 | 0.006 | -0.067 | 0.025 | -2.661 | 0.008 | 0.058 | | 0.029 | 2.02 | 0.043 | -0.052 | | 0.037 | -1.419 | 0.156 | 0.035 | | 0.026 | 1.372 | 0.17 | 0.013 | | 0.014 | 0.913 | 0.361 | -0.003 | | 0.027 | -0.102 | 0.919 | 0.025 | | 0.017 | 1.467 | 0.142 | 0.116 | | 0.03 | 3.836 | 0.0 |
| **Often Moderate Activity (0.0)** | 0.08 | 0.015 | 5.291 | 0.0 | 0.016 | 0.009 | 1.717 | 0.086 | 0.046 | | 0.011 | 4.067 | 0.0 | -0.044 | | 0.01 | -4.493 | 0.0 | -0.001 | | 0.011 | -0.046 | 0.963 | -0.007 | | 0.011 | -0.696 | 0.486 | -0.01 | | 0.011 | -0.937 | 0.349 | -0.026 | | 0.01 | -2.641 | 0.008 | -0.103 | | 0.028 | -3.701 | 0.0 |
| **Often Moderate Activity (1.0)** | -0.084 | 0.024 | -3.515 | 0.0 | -0.016 | 0.011 | -1.445 | 0.148 | -0.049 | | 0.014 | -3.596 | 0.0 | 0.046 | | 0.01 | 4.813 | 0.0 | 0.001 | | 0.011 | 0.047 | 0.963 | 0.008 | | 0.01 | 0.786 | 0.432 | 0.01 | | 0.01 | 1.066 | 0.286 | 0.028 | | 0.008 | 3.412 | 0.001 | 0.109 | | 0.017 | 6.559 | 0.0 |
| **Often Mild Activity (0.0)** | 0.162 | 0.016 | 10.408 | 0.0 | 0.108 | 0.012 | 8.767 | 0.0 | 0.049 | | 0.009 | 5.641 | 0.0 | 0.004 | | 0.012 | 0.349 | 0.727 | 0.003 | | 0.012 | 0.239 | 0.811 | -0.035 | | 0.017 | -2.077 | 0.038 | -0.187 | | 0.02 | -9.515 | 0.0 | -0.091 | | 0.019 | -4.868 | 0.0 | -0.101 | | 0.019 | -5.368 | 0.0 |
| **Often Mild Activity (1.0)** | -0.143 | 0.021 | -6.682 | 0.0 | -0.096 | 0.015 | -6.241 | 0.0 | -0.043 | | 0.009 | -5.035 | 0.0 | -0.004 | | 0.011 | -0.351 | 0.726 | -0.002 | | 0.01 | -0.241 | 0.81 | 0.031 | | 0.013 | 2.414 | 0.016 | 0.166 | | 0.012 | 14.128 | 0.0 | 0.08 | | 0.012 | 6.571 | 0.0 | 0.09 | | 0.011 | 8.343 | 0.0 |
| **Drink Alcohol (0.0)** | 0.202 | 0.015 | 13.28 | 0.0 | 0.102 | 0.016 | 6.225 | 0.0 | 0.096 | | 0.011 | 8.51 | 0.0 | -0.106 | | 0.025 | -4.183 | 0.0 | -0.031 | | 0.011 | -2.769 | 0.006 | -0.044 | | 0.008 | -5.568 | 0.0 | -0.047 | | 0.012 | -3.912 | 0.0 | -0.14 | | 0.016 | -8.513 | 0.0 | -0.145 | | 0.031 | -4.738 | 0.0 |
| **Drink Alcohol (1.0)** | -0.123 | 0.015 | -8.469 | 0.0 | -0.062 | 0.012 | -5.0 | 0.0 | -0.059 | | 0.008 | -7.579 | 0.0 | 0.064 | | 0.013 | 4.807 | 0.0 | 0.019 | | 0.006 | 2.941 | 0.003 | 0.027 | | 0.004 | 6.292 | 0.0 | 0.028 | | 0.007 | 4.364 | 0.0 | 0.086 | | 0.007 | 12.135 | 0.0 | 0.088 | | 0.012 | 7.22 | 0.0 |
| **Education (0.0)** | 0.894 | 0.066 | 13.492 | 0.0 | 0.134 | 0.036 | 3.721 | 0.0 | -0.015 | | 0.087 | -0.168 | 0.867 | -0.148 | | 0.029 | -5.159 | 0.0 | -0.257 | | 0.05 | -5.175 | 0.0 | -0.329 | | 0.044 | -7.431 | 0.0 | -0.471 | | 0.063 | -7.502 | 0.0 | -0.591 | | 0.117 | -5.037 | 0.0 | -0.492 | | 0.187 | -2.629 | 0.009 |
| **Education (0.5)** | 0.894 | 0.066 | 13.492 | 0.0 | 0.134 | 0.036 | 3.721 | 0.0 | -0.015 | | 0.087 | -0.168 | 0.867 | -0.148 | | 0.029 | -5.159 | 0.0 | -0.257 | | 0.05 | -5.175 | 0.0 | -0.329 | | 0.044 | -7.431 | 0.0 | -0.471 | | 0.063 | -7.502 | 0.0 | -0.591 | | 0.117 | -5.037 | 0.0 | -0.492 | | 0.187 | -2.629 | 0.009 |
| **Education (1.5)** | 0.84 | 0.115 | 7.271 | 0.0 | 0.135 | 0.035 | 3.84 | 0.0 | 0.04 | | 0.045 | 0.89 | 0.373 | -0.124 | | 0.053 | -2.336 | 0.019 | -0.24 | | 0.08 | -3.02 | 0.003 | -0.324 | | 0.059 | -5.531 | 0.0 | -0.424 | | 0.082 | -5.198 | 0.0 | -0.553 | | 0.064 | -8.632 | 0.0 | -0.458 | | 0.17 | -2.701 | 0.007 |
| **Education (2.5)** | 0.84 | 0.115 | 7.271 | 0.0 | 0.135 | 0.035 | 3.84 | 0.0 | 0.04 | | 0.045 | 0.89 | 0.373 | -0.124 | | 0.053 | -2.336 | 0.019 | -0.24 | | 0.08 | -3.02 | 0.003 | -0.324 | | 0.059 | -5.531 | 0.0 | -0.424 | | 0.082 | -5.198 | 0.0 | -0.553 | | 0.064 | -8.632 | 0.0 | -0.458 | | 0.17 | -2.701 | 0.007 |
| **Education (4.5)** | 0.664 | 0.143 | 4.654 | 0.0 | 0.163 | 0.029 | 5.596 | 0.0 | 0.057 | | 0.039 | 1.463 | 0.143 | -0.063 | | 0.056 | -1.114 | 0.265 | -0.135 | | 0.084 | -1.608 | 0.108 | -0.229 | | 0.052 | -4.393 | 0.0 | -0.275 | | 0.127 | -2.169 | 0.03 | -0.534 | | 0.072 | -7.368 | 0.0 | -0.477 | | 0.099 | -4.826 | 0.0 |
| **Education (5.5)** | 0.443 | 0.046 | 9.537 | 0.0 | 0.195 | 0.021 | 9.408 | 0.0 | 0.061 | | 0.026 | 2.326 | 0.02 | -0.015 | | 0.018 | -0.793 | 0.428 | -0.04 | | 0.023 | -1.778 | 0.075 | -0.151 | | 0.031 | -4.835 | 0.0 | -0.122 | | 0.035 | -3.475 | 0.001 | -0.48 | | 0.073 | -6.566 | 0.0 | -0.494 | | 0.064 | -7.683 | 0.0 |
| **Education (6.5)** | 0.443 | 0.046 | 9.537 | 0.0 | 0.195 | 0.021 | 9.408 | 0.0 | 0.061 | | 0.026 | 2.326 | 0.02 | -0.015 | | 0.018 | -0.793 | 0.428 | -0.04 | | 0.023 | -1.778 | 0.075 | -0.151 | | 0.031 | -4.835 | 0.0 | -0.122 | | 0.035 | -3.475 | 0.001 | -0.48 | | 0.073 | -6.566 | 0.0 | -0.494 | | 0.064 | -7.683 | 0.0 |
| **Education (7.5)** | 0.443 | 0.046 | 9.537 | 0.0 | 0.195 | 0.021 | 9.408 | 0.0 | 0.061 | | 0.026 | 2.326 | 0.02 | -0.015 | | 0.018 | -0.793 | 0.428 | -0.04 | | 0.023 | -1.778 | 0.075 | -0.151 | | 0.031 | -4.835 | 0.0 | -0.122 | | 0.035 | -3.475 | 0.001 | -0.48 | | 0.073 | -6.566 | 0.0 | -0.494 | | 0.064 | -7.683 | 0.0 |
| **Education (8.5)** | 0.361 | 0.058 | 6.173 | 0.0 | 0.152 | 0.013 | 11.944 | 0.0 | 0.051 | | 0.02 | 2.599 | 0.009 | 0.007 | | 0.019 | 0.372 | 0.71 | 0.012 | | 0.021 | 0.576 | 0.565 | -0.083 | | 0.029 | -2.843 | 0.004 | -0.111 | | 0.028 | -4.009 | 0.0 | -0.379 | | 0.054 | -6.959 | 0.0 | -0.402 | | 0.05 | -8.077 | 0.0 |
| **Education (9.5)** | 0.254 | 0.037 | 6.857 | 0.0 | 0.132 | 0.009 | 14.119 | 0.0 | 0.048 | | 0.012 | 3.919 | 0.0 | 0.036 | | 0.019 | 1.926 | 0.054 | 0.05 | | 0.017 | 2.981 | 0.003 | -0.053 | | 0.018 | -2.943 | 0.003 | -0.083 | | 0.02 | -4.222 | 0.0 | -0.338 | | 0.051 | -6.649 | 0.0 | -0.373 | | 0.049 | -7.626 | 0.0 |
| **Education (10.5)** | 0.254 | 0.037 | 6.873 | 0.0 | 0.131 | 0.009 | 14.254 | 0.0 | 0.046 | | 0.012 | 3.706 | 0.0 | 0.037 | | 0.019 | 1.96 | 0.05 | 0.053 | | 0.017 | 3.168 | 0.002 | -0.052 | | 0.018 | -2.876 | 0.004 | -0.082 | | 0.02 | -4.083 | 0.0 | -0.338 | | 0.051 | -6.643 | 0.0 | -0.376 | | 0.05 | -7.531 | 0.0 |
| **Education (11.5)** | 0.063 | 0.013 | 4.808 | 0.0 | 0.07 | 0.009 | 7.467 | 0.0 | 0.093 | | 0.009 | 10.508 | 0.0 | 0.036 | | 0.01 | 3.643 | 0.0 | 0.047 | | 0.01 | 4.9 | 0.0 | -0.007 | | 0.011 | -0.629 | 0.529 | -0.014 | | 0.009 | -1.525 | 0.127 | -0.119 | | 0.02 | -5.895 | 0.0 | -0.171 | | 0.027 | -6.223 | 0.0 |
| **Education (12.5)** | -0.119 | 0.023 | -5.238 | 0.0 | -0.056 | 0.01 | -5.4 | 0.0 | 0.018 | | 0.017 | 1.044 | 0.296 | -0.036 | | 0.017 | -2.138 | 0.033 | -0.007 | | 0.013 | -0.517 | 0.605 | 0.064 | | 0.025 | 2.618 | 0.009 | 0.027 | | 0.013 | 2.148 | 0.032 | 0.149 | | 0.024 | 6.337 | 0.0 | 0.135 | | 0.023 | 5.913 | 0.0 |
| **Education (13.5)** | -0.128 | 0.021 | -6.116 | 0.0 | -0.061 | 0.01 | -5.799 | 0.0 | 0.019 | | 0.015 | 1.257 | 0.209 | -0.027 | | 0.013 | -2.11 | 0.035 | 0.004 | | 0.011 | 0.34 | 0.734 | 0.038 | | 0.009 | 4.043 | 0.0 | 0.033 | | 0.011 | 3.119 | 0.002 | 0.162 | | 0.021 | 7.845 | 0.0 | 0.134 | | 0.021 | 6.522 | 0.0 |
| **Education (14.5)** | -0.128 | 0.021 | -6.116 | 0.0 | -0.061 | 0.01 | -5.799 | 0.0 | 0.019 | | 0.015 | 1.257 | 0.209 | -0.027 | | 0.013 | -2.11 | 0.035 | 0.004 | | 0.011 | 0.34 | 0.734 | 0.038 | | 0.009 | 4.043 | 0.0 | 0.033 | | 0.011 | 3.119 | 0.002 | 0.162 | | 0.021 | 7.845 | 0.0 | 0.134 | | 0.021 | 6.522 | 0.0 |
| **Education (15.5)** | -0.207 | 0.023 | -8.996 | 0.0 | -0.127 | 0.013 | -9.969 | 0.0 | -0.131 | | 0.029 | -4.534 | 0.0 | -0.039 | | 0.02 | -1.965 | 0.049 | -0.011 | | 0.022 | -0.489 | 0.625 | 0.039 | | 0.014 | 2.7 | 0.007 | 0.075 | | 0.006 | 12.216 | 0.0 | 0.276 | | 0.019 | 14.787 | 0.0 | 0.3 | | 0.048 | 6.287 | 0.0 |
| **Education (16.5)** | -0.252 | 0.031 | -8.229 | 0.0 | -0.167 | 0.022 | -7.611 | 0.0 | -0.236 | | 0.03 | -7.747 | 0.0 | -0.005 | | 0.026 | -0.183 | 0.855 | -0.113 | | 0.047 | -2.435 | 0.015 | 0.039 | | 0.023 | 1.695 | 0.09 | 0.085 | | 0.011 | 7.497 | 0.0 | 0.314 | | 0.016 | 20.116 | 0.0 | 0.506 | | 0.055 | 9.18 | 0.0 |
| **Education (17.0)** | -0.252 | 0.031 | -8.229 | 0.0 | -0.167 | 0.022 | -7.611 | 0.0 | -0.236 | | 0.03 | -7.747 | 0.0 | -0.005 | | 0.026 | -0.183 | 0.855 | -0.113 | | 0.047 | -2.435 | 0.015 | 0.039 | | 0.023 | 1.695 | 0.09 | 0.085 | | 0.011 | 7.497 | 0.0 | 0.314 | | 0.016 | 20.116 | 0.0 | 0.506 | | 0.055 | 9.18 | 0.0 |
| **Smoking (0.0)** | -0.009 | 0.008 | -1.128 | 0.259 | -0.027 | 0.005 | -5.943 | 0.0 | 0.013 | | 0.005 | 2.598 | 0.009 | 0.015 | | 0.005 | 3.021 | 0.003 | -0.008 | | 0.006 | -1.325 | 0.185 | 0.003 | | 0.005 | 0.653 | 0.514 | -0.001 | | 0.007 | -0.081 | 0.935 | 0.017 | | 0.003 | 5.567 | 0.0 | 0.017 | | 0.009 | 1.857 | 0.063 |
| **Smoking (1.0)** | 0.043 | 0.032 | 1.317 | 0.188 | 0.132 | 0.02 | 6.522 | 0.0 | -0.065 | | 0.025 | -2.647 | 0.008 | -0.074 | | 0.028 | -2.671 | 0.008 | 0.041 | | 0.03 | 1.378 | 0.168 | -0.016 | | 0.025 | -0.62 | 0.535 | 0.003 | | 0.033 | 0.08 | 0.936 | -0.084 | | 0.018 | -4.746 | 0.0 | -0.083 | | 0.058 | -1.444 | 0.149 |
| **Age (60.0)** | -0.237 | 0.03 | -7.916 | 0.0 | -0.138 | 0.012 | -11.098 | 0.0 | -0.043 | | 0.032 | -1.351 | 0.177 | -0.007 | | 0.01 | -0.754 | 0.451 | 0.031 | | 0.012 | 2.635 | 0.008 | 0.115 | | 0.015 | 7.495 | 0.0 | 0.108 | | 0.013 | 8.041 | 0.0 | 0.143 | | 0.01 | 14.047 | 0.0 | 0.265 | | 0.028 | 9.448 | 0.0 |
| **Age (60.5)** | -0.237 | 0.03 | -7.916 | 0.0 | -0.138 | 0.012 | -11.098 | 0.0 | -0.043 | | 0.032 | -1.351 | 0.177 | -0.007 | | 0.01 | -0.754 | 0.451 | 0.031 | | 0.012 | 2.635 | 0.008 | 0.115 | | 0.015 | 7.495 | 0.0 | 0.108 | | 0.013 | 8.041 | 0.0 | 0.143 | | 0.01 | 14.047 | 0.0 | 0.265 | | 0.028 | 9.448 | 0.0 |
| **Age (61.5)** | -0.231 | 0.024 | -9.466 | 0.0 | -0.145 | 0.014 | -10.477 | 0.0 | -0.044 | | 0.032 | -1.372 | 0.17 | -0.005 | | 0.008 | -0.642 | 0.521 | 0.032 | | 0.012 | 2.69 | 0.007 | 0.116 | | 0.017 | 7.022 | 0.0 | 0.107 | | 0.013 | 8.507 | 0.0 | 0.145 | | 0.012 | 12.259 | 0.0 | 0.261 | | 0.023 | 11.561 | 0.0 |
| **Age (62.5)** | -0.231 | 0.024 | -9.466 | 0.0 | -0.145 | 0.014 | -10.477 | 0.0 | -0.044 | | 0.032 | -1.372 | 0.17 | -0.005 | | 0.008 | -0.642 | 0.521 | 0.032 | | 0.012 | 2.69 | 0.007 | 0.116 | | 0.017 | 7.022 | 0.0 | 0.107 | | 0.013 | 8.507 | 0.0 | 0.145 | | 0.012 | 12.259 | 0.0 | 0.261 | | 0.023 | 11.561 | 0.0 |
| **Age (63.5)** | -0.231 | 0.024 | -9.466 | 0.0 | -0.145 | 0.014 | -10.477 | 0.0 | -0.044 | | 0.032 | -1.372 | 0.17 | -0.005 | | 0.008 | -0.642 | 0.521 | 0.032 | | 0.012 | 2.69 | 0.007 | 0.116 | | 0.017 | 7.022 | 0.0 | 0.107 | | 0.013 | 8.507 | 0.0 | 0.145 | | 0.012 | 12.259 | 0.0 | 0.261 | | 0.023 | 11.561 | 0.0 |
| **Age (64.5)** | -0.234 | 0.026 | -9.072 | 0.0 | -0.148 | 0.016 | -9.054 | 0.0 | -0.034 | | 0.025 | -1.347 | 0.178 | -0.001 | | 0.009 | -0.098 | 0.922 | 0.032 | | 0.011 | 2.924 | 0.003 | 0.116 | | 0.016 | 7.418 | 0.0 | 0.108 | | 0.012 | 8.969 | 0.0 | 0.141 | | 0.01 | 14.401 | 0.0 | 0.255 | | 0.016 | 16.073 | 0.0 |
| **Age (65.5)** | -0.234 | 0.026 | -9.072 | 0.0 | -0.148 | 0.016 | -9.054 | 0.0 | -0.034 | | 0.025 | -1.347 | 0.178 | -0.001 | | 0.009 | -0.098 | 0.922 | 0.032 | | 0.011 | 2.924 | 0.003 | 0.116 | | 0.016 | 7.418 | 0.0 | 0.108 | | 0.012 | 8.969 | 0.0 | 0.141 | | 0.01 | 14.401 | 0.0 | 0.255 | | 0.016 | 16.073 | 0.0 |
| **Age (66.5)** | -0.233 | 0.026 | -9.061 | 0.0 | -0.148 | 0.016 | -9.003 | 0.0 | -0.033 | | 0.026 | -1.246 | 0.213 | 0.001 | | 0.01 | 0.068 | 0.946 | 0.032 | | 0.011 | 3.013 | 0.003 | 0.115 | | 0.016 | 7.249 | 0.0 | 0.107 | | 0.012 | 9.178 | 0.0 | 0.14 | | 0.011 | 13.304 | 0.0 | 0.253 | | 0.018 | 13.886 | 0.0 |
| **Age (67.5)** | -0.233 | 0.026 | -9.061 | 0.0 | -0.148 | 0.016 | -9.003 | 0.0 | -0.033 | | 0.026 | -1.246 | 0.213 | 0.001 | | 0.01 | 0.068 | 0.946 | 0.032 | | 0.011 | 3.013 | 0.003 | 0.115 | | 0.016 | 7.249 | 0.0 | 0.107 | | 0.012 | 9.178 | 0.0 | 0.14 | | 0.011 | 13.304 | 0.0 | 0.253 | | 0.018 | 13.886 | 0.0 |
| **Age (68.5)** | -0.227 | 0.026 | -8.887 | 0.0 | -0.144 | 0.015 | -9.735 | 0.0 | 0.006 | | 0.019 | 0.304 | 0.761 | 0.006 | | 0.01 | 0.57 | 0.569 | 0.037 | | 0.009 | 3.945 | 0.0 | 0.094 | | 0.011 | 8.656 | 0.0 | 0.107 | | 0.012 | 9.056 | 0.0 | 0.127 | | 0.008 | 16.821 | 0.0 | 0.219 | | 0.018 | 11.873 | 0.0 |
| **Age (69.5)** | -0.227 | 0.025 | -8.947 | 0.0 | -0.145 | 0.016 | -9.223 | 0.0 | 0.009 | | 0.021 | 0.428 | 0.669 | 0.008 | | 0.013 | 0.592 | 0.554 | 0.037 | | 0.009 | 3.91 | 0.0 | 0.092 | | 0.01 | 9.089 | 0.0 | 0.106 | | 0.012 | 8.52 | 0.0 | 0.127 | | 0.007 | 17.948 | 0.0 | 0.216 | | 0.018 | 11.934 | 0.0 |
| **Age (70.5)** | -0.203 | 0.035 | -5.856 | 0.0 | -0.128 | 0.022 | -5.903 | 0.0 | 0.032 | | 0.03 | 1.079 | 0.281 | 0.016 | | 0.018 | 0.898 | 0.369 | 0.039 | | 0.01 | 4.039 | 0.0 | 0.066 | | 0.023 | 2.919 | 0.004 | 0.094 | | 0.02 | 4.599 | 0.0 | 0.117 | | 0.016 | 7.484 | 0.0 | 0.18 | | 0.043 | 4.178 | 0.0 |
| **Age (71.5)** | -0.159 | 0.042 | -3.829 | 0.0 | -0.081 | 0.031 | -2.637 | 0.008 | 0.058 | | 0.022 | 2.595 | 0.009 | 0.022 | | 0.015 | 1.417 | 0.156 | 0.04 | | 0.009 | 4.457 | 0.0 | 0.031 | | 0.02 | 1.576 | 0.115 | 0.062 | | 0.021 | 2.968 | 0.003 | 0.094 | | 0.014 | 6.506 | 0.0 | 0.113 | | 0.032 | 3.558 | 0.0 |
| **Age (72.5)** | -0.154 | 0.039 | -3.923 | 0.0 | -0.078 | 0.03 | -2.584 | 0.01 | 0.06 | | 0.019 | 3.112 | 0.002 | 0.024 | | 0.013 | 1.888 | 0.059 | 0.04 | | 0.009 | 4.454 | 0.0 | 0.029 | | 0.016 | 1.786 | 0.074 | 0.058 | | 0.019 | 3.117 | 0.002 | 0.092 | | 0.012 | 7.844 | 0.0 | 0.107 | | 0.021 | 4.993 | 0.0 |
| **Age (73.5)** | -0.106 | 0.059 | -1.785 | 0.074 | -0.041 | 0.042 | -0.972 | 0.331 | 0.078 | | 0.017 | 4.62 | 0.0 | 0.029 | | 0.014 | 2.092 | 0.036 | 0.03 | | 0.013 | 2.345 | 0.019 | 0.009 | | 0.019 | 0.467 | 0.64 | 0.03 | | 0.031 | 0.968 | 0.333 | 0.063 | | 0.029 | 2.205 | 0.027 | 0.056 | | 0.038 | 1.457 | 0.145 |
| **Age (74.5)** | 0.108 | 0.027 | 3.949 | 0.0 | 0.096 | 0.014 | 6.647 | 0.0 | 0.087 | | 0.024 | 3.687 | 0.0 | 0.043 | | 0.016 | 2.716 | 0.007 | 0.005 | | 0.011 | 0.477 | 0.633 | -0.074 | | 0.024 | -3.134 | 0.002 | -0.061 | | 0.017 | -3.602 | 0.0 | -0.046 | | 0.015 | -3.052 | 0.002 | -0.156 | | 0.033 | -4.781 | 0.0 |
| **Age (75.5)** | 0.108 | 0.027 | 3.949 | 0.0 | 0.096 | 0.014 | 6.647 | 0.0 | 0.087 | | 0.024 | 3.687 | 0.0 | 0.043 | | 0.016 | 2.716 | 0.007 | 0.005 | | 0.011 | 0.477 | 0.633 | -0.074 | | 0.024 | -3.134 | 0.002 | -0.061 | | 0.017 | -3.602 | 0.0 | -0.046 | | 0.015 | -3.052 | 0.002 | -0.156 | | 0.033 | -4.781 | 0.0 |
| **Age (76.5)** | 0.11 | 0.026 | 4.148 | 0.0 | 0.098 | 0.016 | 6.251 | 0.0 | 0.086 | | 0.024 | 3.516 | 0.0 | 0.044 | | 0.016 | 2.771 | 0.006 | 0.005 | | 0.011 | 0.478 | 0.633 | -0.075 | | 0.023 | -3.315 | 0.001 | -0.062 | | 0.017 | -3.557 | 0.0 | -0.047 | | 0.015 | -3.099 | 0.002 | -0.159 | | 0.032 | -4.912 | 0.0 |
| **Age (77.5)** | 0.118 | 0.022 | 5.368 | 0.0 | 0.101 | 0.015 | 6.943 | 0.0 | 0.085 | | 0.023 | 3.624 | 0.0 | 0.047 | | 0.02 | 2.341 | 0.019 | 0.003 | | 0.012 | 0.296 | 0.767 | -0.078 | | 0.022 | -3.63 | 0.0 | -0.066 | | 0.021 | -3.083 | 0.002 | -0.051 | | 0.014 | -3.675 | 0.0 | -0.17 | | 0.032 | -5.311 | 0.0 |
| **Age (78.5)** | 0.228 | 0.058 | 3.903 | 0.0 | 0.155 | 0.043 | 3.586 | 0.0 | 0.081 | | 0.02 | 4.034 | 0.0 | 0.079 | | 0.024 | 3.226 | 0.001 | 0.003 | | 0.018 | 0.151 | 0.88 | -0.126 | | 0.031 | -4.109 | 0.0 | -0.143 | | 0.048 | -2.968 | 0.003 | -0.115 | | 0.035 | -3.289 | 0.001 | -0.294 | | 0.07 | -4.228 | 0.0 |
| **Age (79.5)** | 0.229 | 0.058 | 3.924 | 0.0 | 0.155 | 0.043 | 3.59 | 0.0 | 0.081 | | 0.02 | 4.154 | 0.0 | 0.079 | | 0.024 | 3.367 | 0.001 | 0.003 | | 0.018 | 0.167 | 0.867 | -0.127 | | 0.03 | -4.186 | 0.0 | -0.144 | | 0.047 | -3.044 | 0.002 | -0.116 | | 0.034 | -3.368 | 0.001 | -0.295 | | 0.07 | -4.245 | 0.0 |
| **Age (80.5)** | 0.555 | 0.079 | 7.066 | 0.0 | 0.29 | 0.036 | 8.093 | 0.0 | -0.009 | | 0.04 | -0.237 | 0.813 | 0.056 | | 0.026 | 2.148 | 0.032 | -0.059 | | 0.021 | -2.793 | 0.005 | -0.243 | | 0.043 | -5.71 | 0.0 | -0.263 | | 0.056 | -4.674 | 0.0 | -0.344 | | 0.062 | -5.517 | 0.0 | -0.497 | | 0.086 | -5.789 | 0.0 |
| **Age (81.5)** | 0.554 | 0.078 | 7.128 | 0.0 | 0.294 | 0.039 | 7.573 | 0.0 | -0.008 | | 0.038 | -0.208 | 0.835 | 0.046 | | 0.02 | 2.355 | 0.019 | -0.064 | | 0.025 | -2.516 | 0.012 | -0.238 | | 0.038 | -6.302 | 0.0 | -0.259 | | 0.053 | -4.916 | 0.0 | -0.343 | | 0.061 | -5.58 | 0.0 | -0.499 | | 0.087 | -5.718 | 0.0 |
| **Age (82.5)** | 0.554 | 0.078 | 7.128 | 0.0 | 0.294 | 0.039 | 7.573 | 0.0 | -0.008 | | 0.038 | -0.208 | 0.835 | 0.046 | | 0.02 | 2.355 | 0.019 | -0.064 | | 0.025 | -2.516 | 0.012 | -0.238 | | 0.038 | -6.302 | 0.0 | -0.259 | | 0.053 | -4.916 | 0.0 | -0.343 | | 0.061 | -5.58 | 0.0 | -0.499 | | 0.087 | -5.718 | 0.0 |
| **Age (83.5)** | 0.842 | 0.042 | 20.08 | 0.0 | 0.478 | 0.037 | 13.029 | 0.0 | -0.145 | | 0.051 | -2.816 | 0.005 | -0.209 | | 0.045 | -4.618 | 0.0 | -0.221 | | 0.04 | -5.473 | 0.0 | -0.287 | | 0.055 | -5.263 | 0.0 | -0.31 | | 0.029 | -10.7 | 0.0 | -0.551 | | 0.079 | -6.99 | 0.0 | -0.787 | | 0.115 | -6.842 | 0.0 |
| **Age (84.5)** | 0.842 | 0.042 | 20.08 | 0.0 | 0.478 | 0.037 | 13.029 | 0.0 | -0.145 | | 0.051 | -2.816 | 0.005 | -0.209 | | 0.045 | -4.618 | 0.0 | -0.221 | | 0.04 | -5.473 | 0.0 | -0.287 | | 0.055 | -5.263 | 0.0 | -0.31 | | 0.029 | -10.7 | 0.0 | -0.551 | | 0.079 | -6.99 | 0.0 | -0.787 | | 0.115 | -6.842 | 0.0 |
| **Age (85.5)** | 0.842 | 0.042 | 20.08 | 0.0 | 0.478 | 0.037 | 13.029 | 0.0 | -0.145 | | 0.051 | -2.816 | 0.005 | -0.209 | | 0.045 | -4.618 | 0.0 | -0.221 | | 0.04 | -5.473 | 0.0 | -0.287 | | 0.055 | -5.263 | 0.0 | -0.31 | | 0.029 | -10.7 | 0.0 | -0.551 | | 0.079 | -6.99 | 0.0 | -0.787 | | 0.115 | -6.842 | 0.0 |
| **Age (86.5)** | 0.842 | 0.042 | 20.08 | 0.0 | 0.478 | 0.037 | 13.029 | 0.0 | -0.145 | | 0.051 | -2.816 | 0.005 | -0.209 | | 0.045 | -4.618 | 0.0 | -0.221 | | 0.04 | -5.473 | 0.0 | -0.287 | | 0.055 | -5.263 | 0.0 | -0.31 | | 0.029 | -10.7 | 0.0 | -0.551 | | 0.079 | -6.99 | 0.0 | -0.787 | | 0.115 | -6.842 | 0.0 |
| **Age (87.5)** | 0.842 | 0.042 | 20.08 | 0.0 | 0.478 | 0.037 | 13.029 | 0.0 | -0.145 | | 0.051 | -2.816 | 0.005 | -0.209 | | 0.045 | -4.618 | 0.0 | -0.221 | | 0.04 | -5.473 | 0.0 | -0.287 | | 0.055 | -5.263 | 0.0 | -0.31 | | 0.029 | -10.7 | 0.0 | -0.551 | | 0.079 | -6.99 | 0.0 | -0.787 | | 0.115 | -6.842 | 0.0 |
| **Age (88.5)** | 0.842 | 0.042 | 20.08 | 0.0 | 0.478 | 0.037 | 13.029 | 0.0 | -0.145 | | 0.051 | -2.816 | 0.005 | -0.209 | | 0.045 | -4.618 | 0.0 | -0.221 | | 0.04 | -5.473 | 0.0 | -0.287 | | 0.055 | -5.263 | 0.0 | -0.31 | | 0.029 | -10.7 | 0.0 | -0.551 | | 0.079 | -6.99 | 0.0 | -0.787 | | 0.115 | -6.842 | 0.0 |
| **Age (89.5)** | 0.842 | 0.042 | 20.08 | 0.0 | 0.478 | 0.037 | 13.029 | 0.0 | -0.145 | | 0.051 | -2.816 | 0.005 | -0.209 | | 0.045 | -4.618 | 0.0 | -0.221 | | 0.04 | -5.473 | 0.0 | -0.287 | | 0.055 | -5.263 | 0.0 | -0.31 | | 0.029 | -10.7 | 0.0 | -0.551 | | 0.079 | -6.99 | 0.0 | -0.787 | | 0.115 | -6.842 | 0.0 |
| **Age (90.5)** | 0.842 | 0.042 | 20.08 | 0.0 | 0.478 | 0.037 | 13.029 | 0.0 | -0.145 | | 0.051 | -2.816 | 0.005 | -0.209 | | 0.045 | -4.618 | 0.0 | -0.221 | | 0.04 | -5.473 | 0.0 | -0.287 | | 0.055 | -5.263 | 0.0 | -0.31 | | 0.029 | -10.7 | 0.0 | -0.551 | | 0.079 | -6.99 | 0.0 | -0.787 | | 0.115 | -6.842 | 0.0 |
| **Age (91.5)** | 0.894 | 0.063 | 14.278 | 0.0 | 0.484 | 0.042 | 11.489 | 0.0 | -0.148 | | 0.05 | -2.951 | 0.003 | -0.256 | | 0.051 | -4.994 | 0.0 | -0.256 | | 0.07 | -3.668 | 0.0 | -0.322 | | 0.076 | -4.212 | 0.0 | -0.348 | | 0.048 | -7.242 | 0.0 | -0.586 | | 0.072 | -8.153 | 0.0 | -0.81 | | 0.111 | -7.272 | 0.0 |
| **Age (92.5)** | 1.053 | 0.142 | 7.418 | 0.0 | 0.476 | 0.053 | 8.947 | 0.0 | -0.152 | | 0.055 | -2.768 | 0.006 | -0.365 | | 0.079 | -4.636 | 0.0 | -0.359 | | 0.106 | -3.378 | 0.001 | -0.412 | | 0.093 | -4.411 | 0.0 | -0.433 | | 0.092 | -4.713 | 0.0 | -0.664 | | 0.101 | -6.602 | 0.0 | -0.862 | | 0.122 | -7.089 | 0.0 |
| **Age (93.5)** | 1.053 | 0.142 | 7.418 | 0.0 | 0.476 | 0.053 | 8.947 | 0.0 | -0.152 | | 0.055 | -2.768 | 0.006 | -0.365 | | 0.079 | -4.636 | 0.0 | -0.359 | | 0.106 | -3.378 | 0.001 | -0.412 | | 0.093 | -4.411 | 0.0 | -0.433 | | 0.092 | -4.713 | 0.0 | -0.664 | | 0.101 | -6.602 | 0.0 | -0.862 | | 0.122 | -7.089 | 0.0 |
| **Age (94.5)** | 1.083 | 0.149 | 7.285 | 0.0 | 0.459 | 0.084 | 5.465 | 0.0 | -0.164 | | 0.056 | -2.917 | 0.004 | -0.377 | | 0.073 | -5.166 | 0.0 | -0.371 | | 0.107 | -3.467 | 0.001 | -0.422 | | 0.086 | -4.898 | 0.0 | -0.442 | | 0.093 | -4.755 | 0.0 | -0.672 | | 0.094 | -7.121 | 0.0 | -0.866 | | 0.118 | -7.359 | 0.0 |
| **Age (96.0)** | 1.083 | 0.149 | 7.285 | 0.0 | 0.459 | 0.084 | 5.465 | 0.0 | -0.164 | | 0.056 | -2.917 | 0.004 | -0.377 | | 0.073 | -5.166 | 0.0 | -0.371 | | 0.107 | -3.467 | 0.001 | -0.422 | | 0.086 | -4.898 | 0.0 | -0.442 | | 0.093 | -4.755 | 0.0 | -0.672 | | 0.094 | -7.121 | 0.0 | -0.866 | | 0.118 | -7.359 | 0.0 |
| **Age (99.0)** | 1.083 | 0.149 | 7.285 | 0.0 | 0.459 | 0.084 | 5.465 | 0.0 | -0.164 | | 0.056 | -2.917 | 0.004 | -0.377 | | 0.073 | -5.166 | 0.0 | -0.371 | | 0.107 | -3.467 | 0.001 | -0.422 | | 0.086 | -4.898 | 0.0 | -0.442 | | 0.093 | -4.755 | 0.0 | -0.672 | | 0.094 | -7.121 | 0.0 | -0.866 | | 0.118 | -7.359 | 0.0 |

*Age and Education Distributions*

Figure 13 describes the distribution of “*Age”* and “*Education”.* As can be noticed both distributions are skewed. The effect of the skewness is reflected on the high variability of the EBM outputs in these ranges as reported in Figure 1 in the main manuscript and remaining figures.


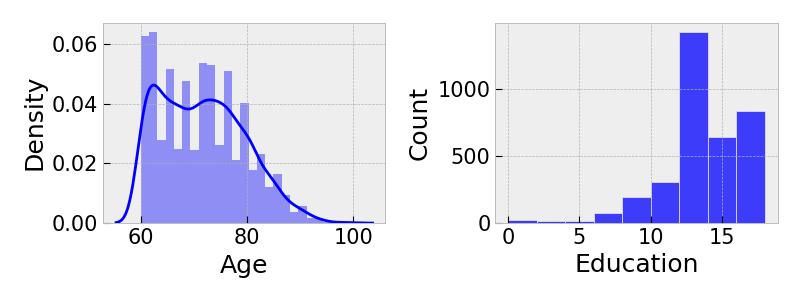


Supplementary Figure 13: Age and Education distribution.

*Model Interpretability Comparison*

We selected a data subject and compared the local interpretations of the EBM model against the local interpretations obtained from LIME and SHAP on the LR, XGB, SVC, RF, and MLP models. For LIME explanations, left, central, and right subplots respectively correspond to the prediction probabilities for each cognitive group, the feature contribution, and the five most important features determined by the technique. The figures show that for most of the models, the important variables are Age, Often Use Computer, Education, Often Do Mild Activity, and Drink Alcohol. The predicted cognitive group of the selected subject of all models is cognitive group 1 except for the MLP model which predicted cognitive group 2. For SHAP explanation figures, the x-axes correspond to the expected log loss of the model, and the y-axes the positive or negative contribution of each feature to the expected log loss. We set the maximum number of features on the y-axes to 10, therefore, the 10^th^ row represents the aggregate contribution of the remaining features. Overall, the cumulative sum of the expected log loss and feature contributions correspond to the value of f(X) in the figures.

While the EBM, LIME, and SHAP provide similar local explanations, i.e., the contribution of each feature to the model outcome, for the global explanations discussed in the main paper, only the EBM model provides the contribution of each independent variable category.


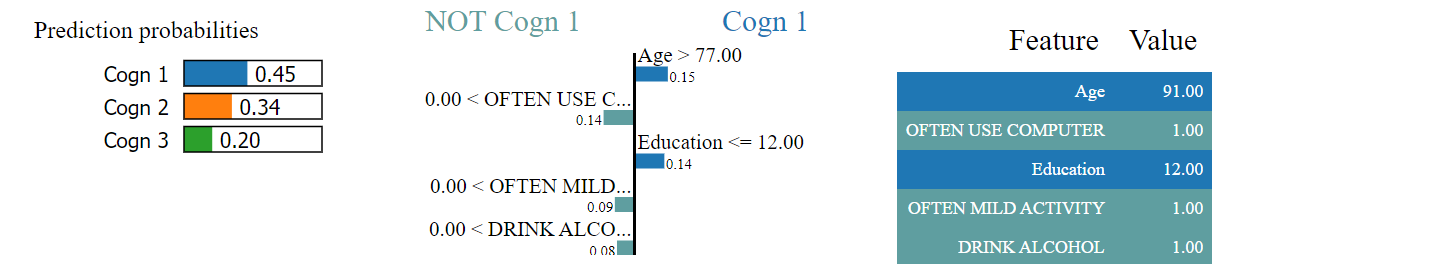


Supplementary Figure 14: Explanations obtained from LIME for the LR model.


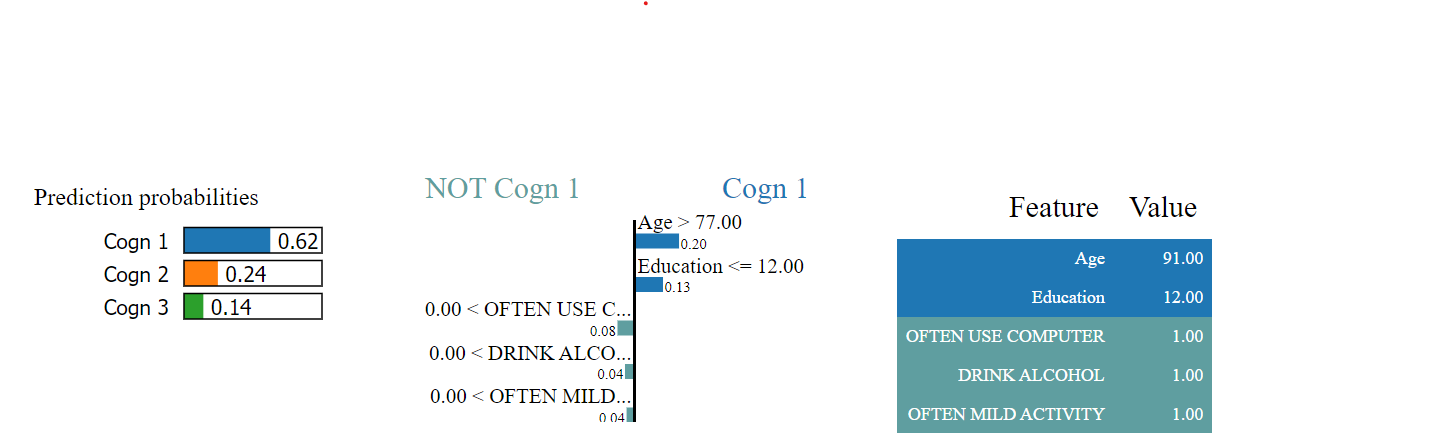


Supplementary Figure 15: Explanations obtained from LIME for the SVM model.


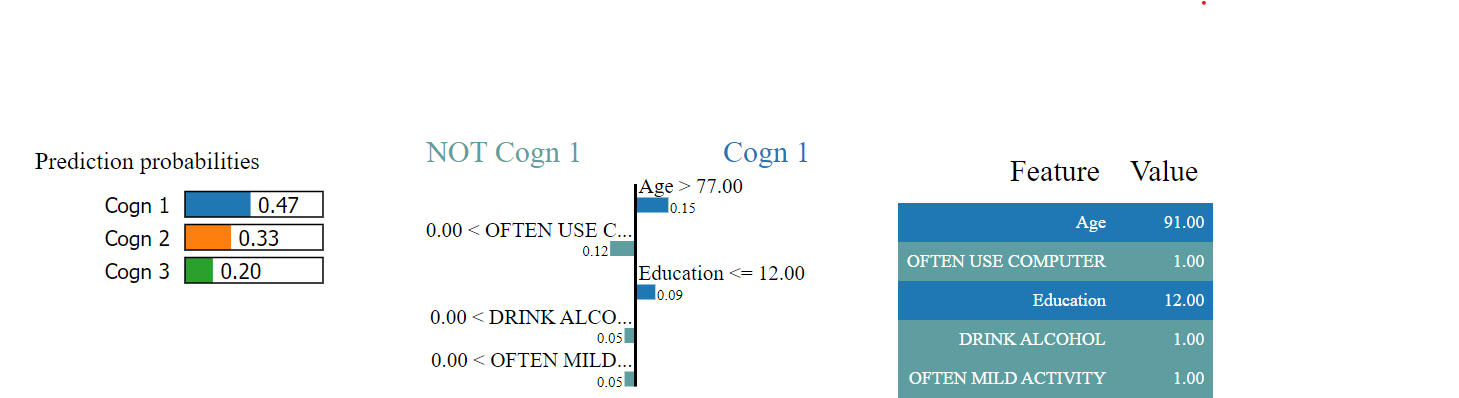


Supplementary Figure 16: Explanations obtained from LIME for the RF model.


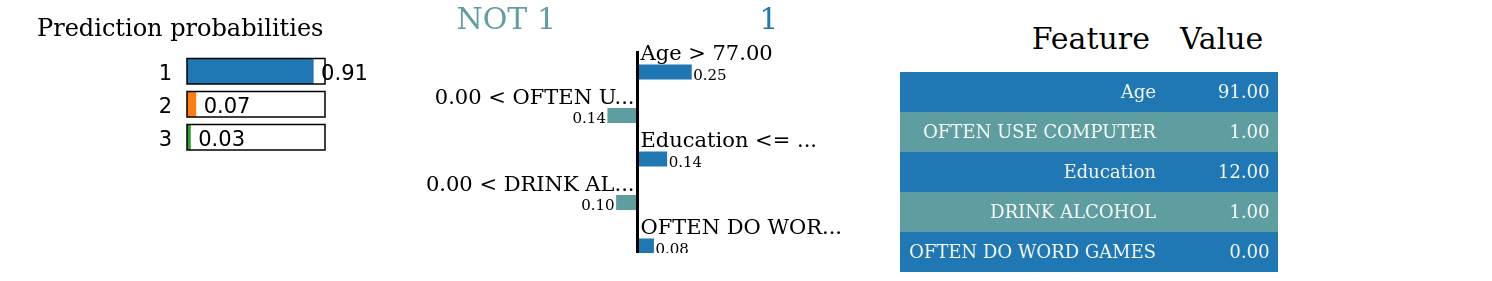


Supplementary Figure 17: Explanations obtained from LIME for the XGB model.


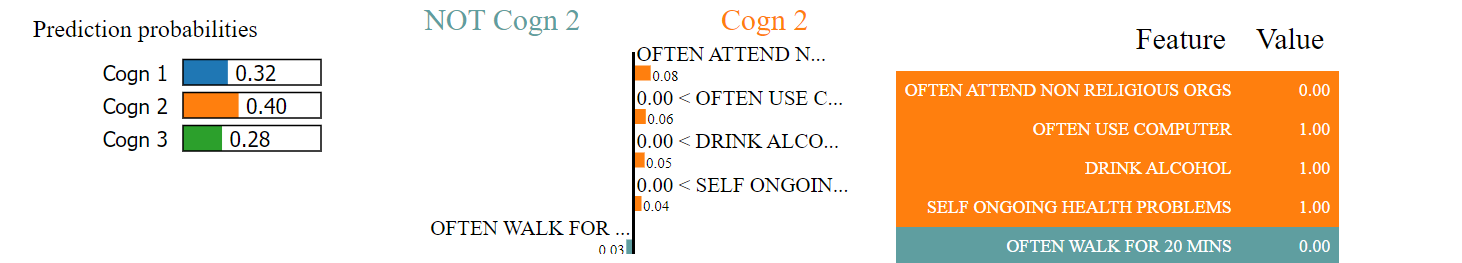


Supplementary Figure 18: Explanations obtained from LIME for the MLP.


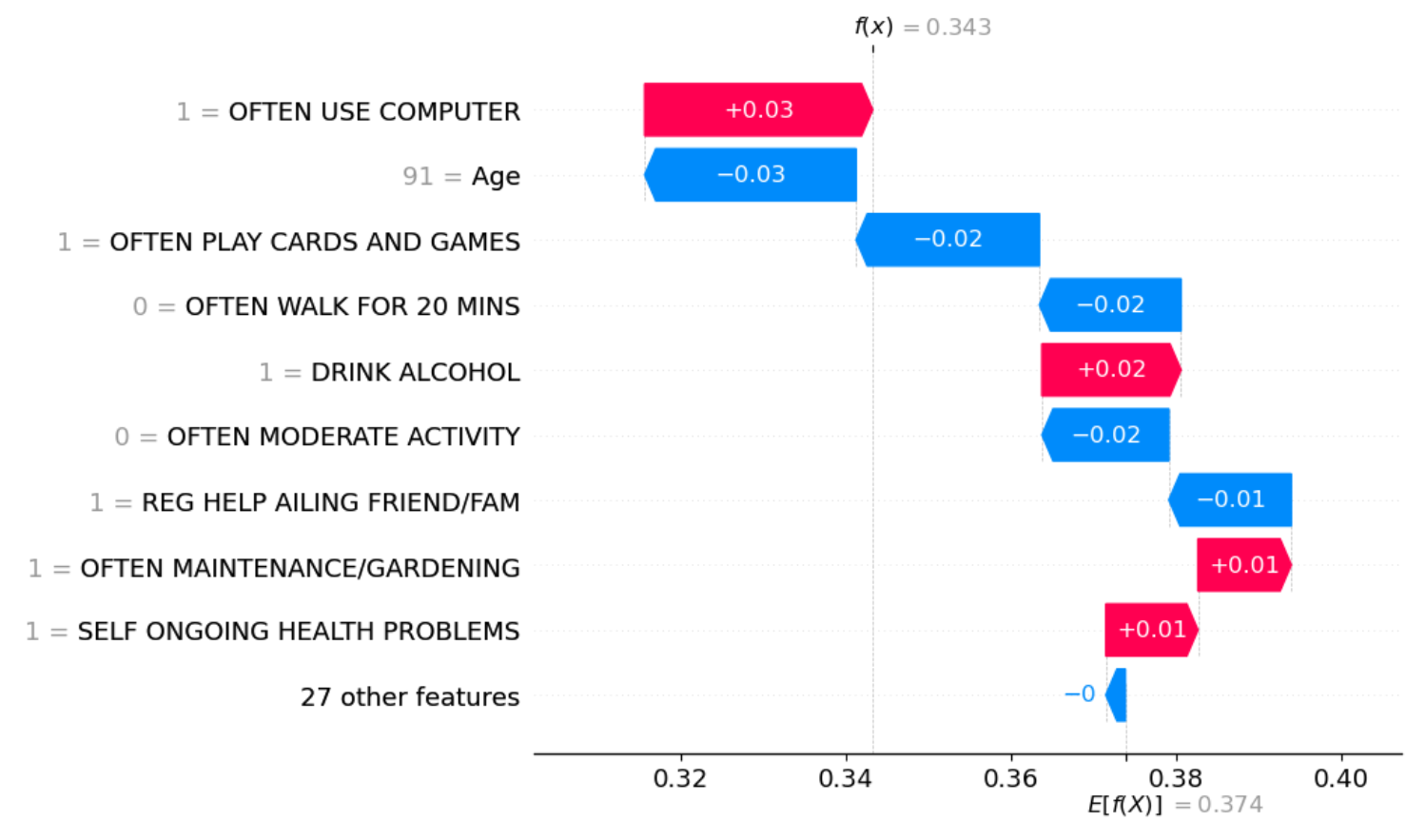


Supplementary Figure 19: Explanations obtained from SHAP for the LR model.


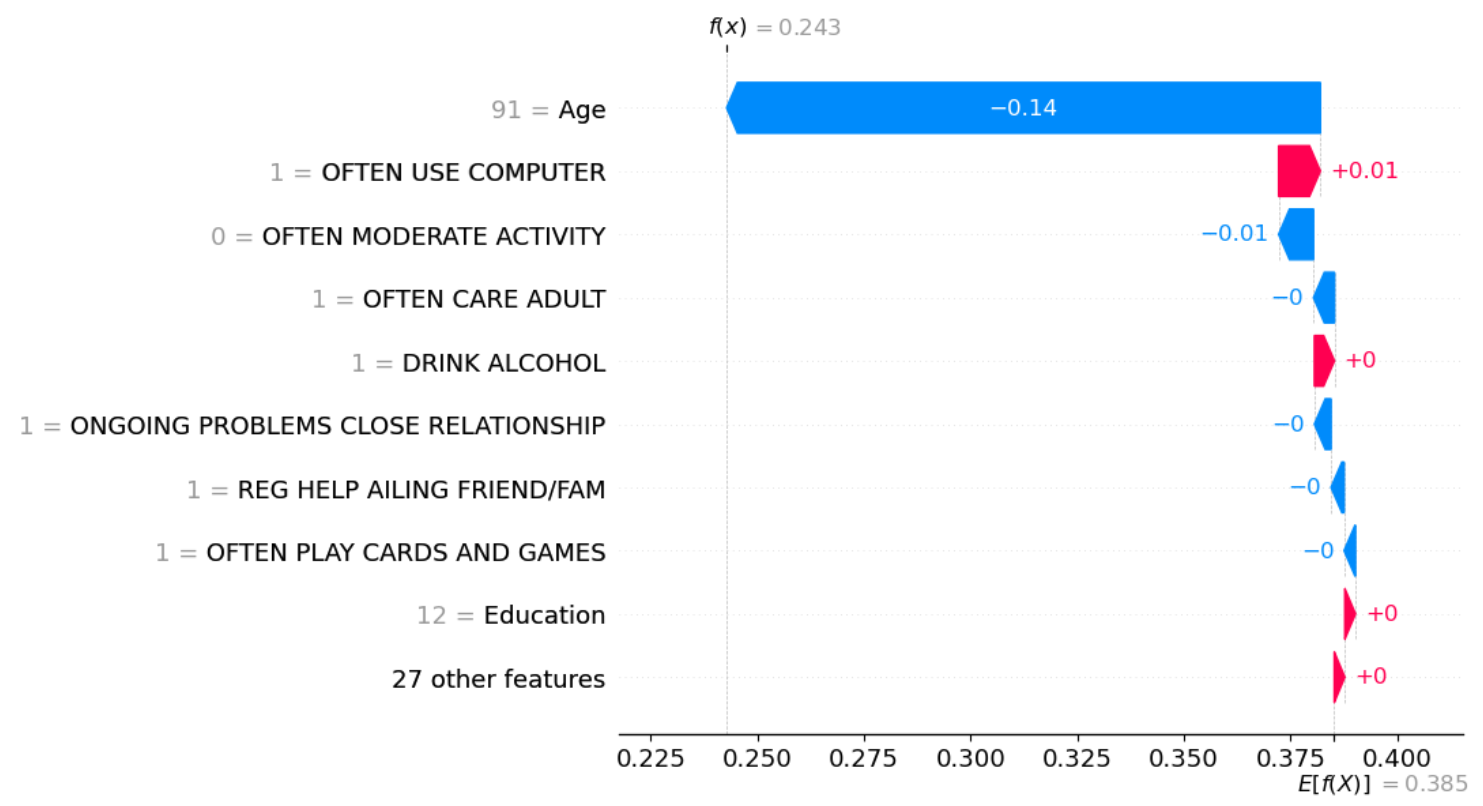


Supplementary Figure 20: Explanations obtained from SHAP for the SVM model.


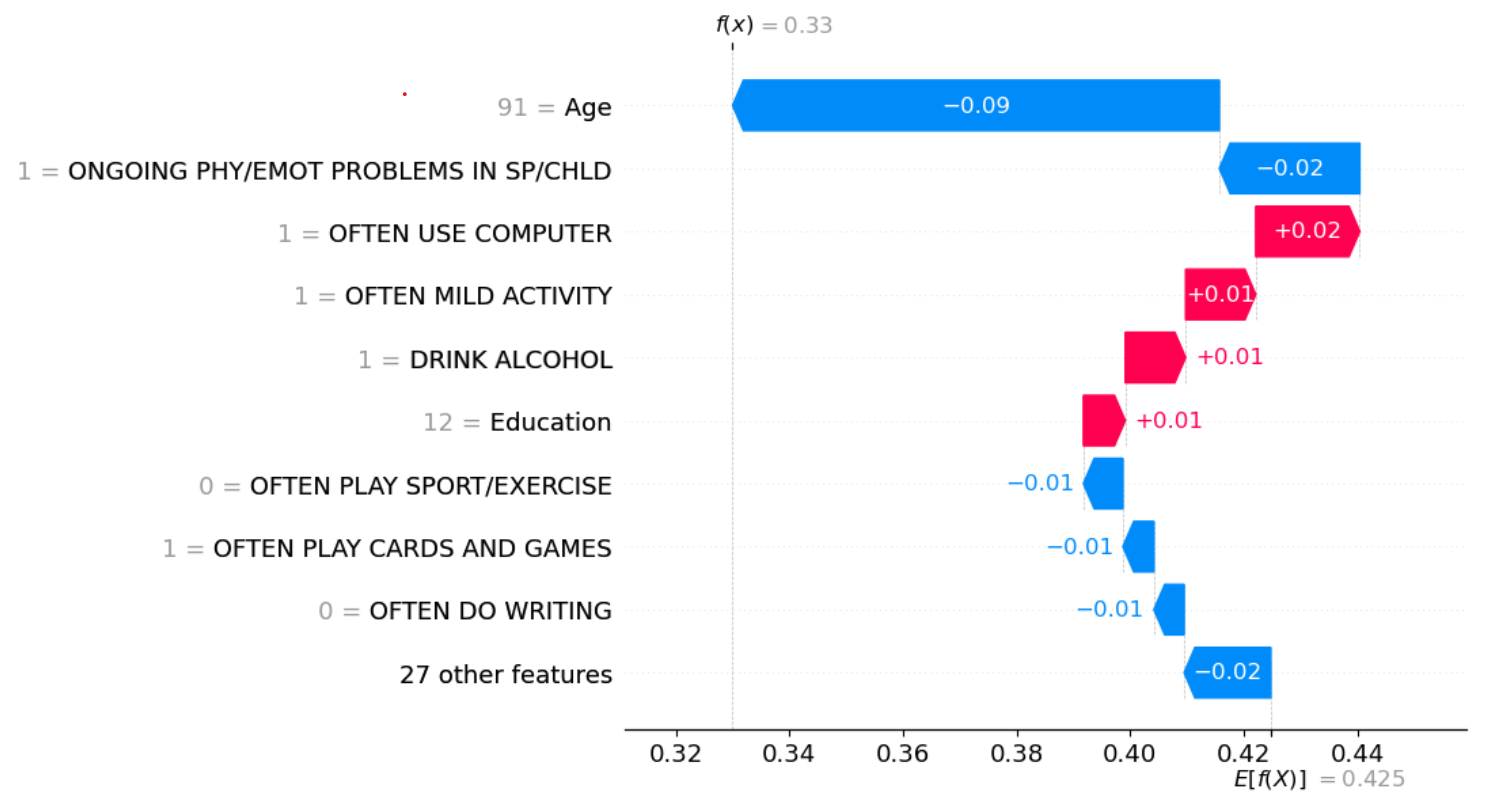


Supplementary Figure 21: Explanations obtained from SHAP for the RF model.


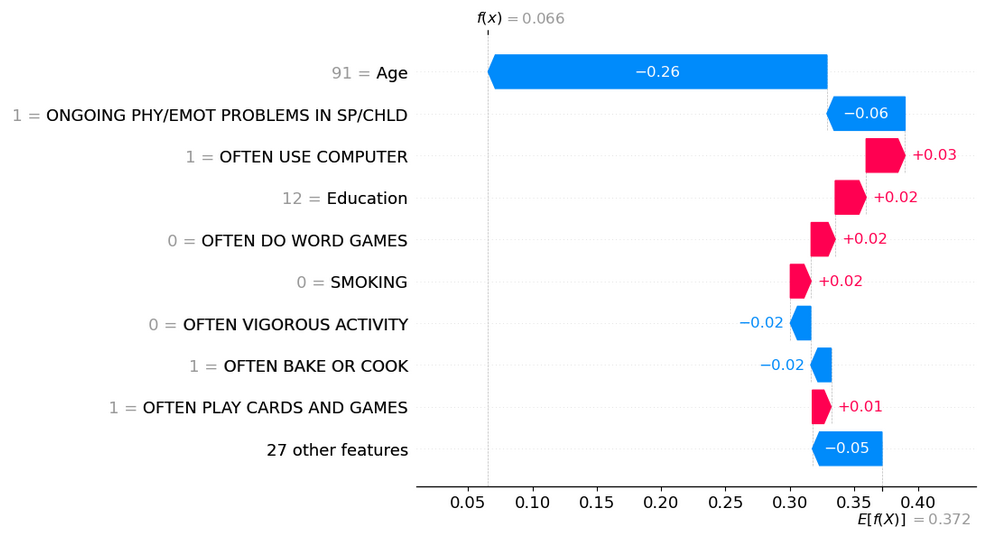


Supplementary Figure 22: Explanations obtained from SHAP for the XGB model.


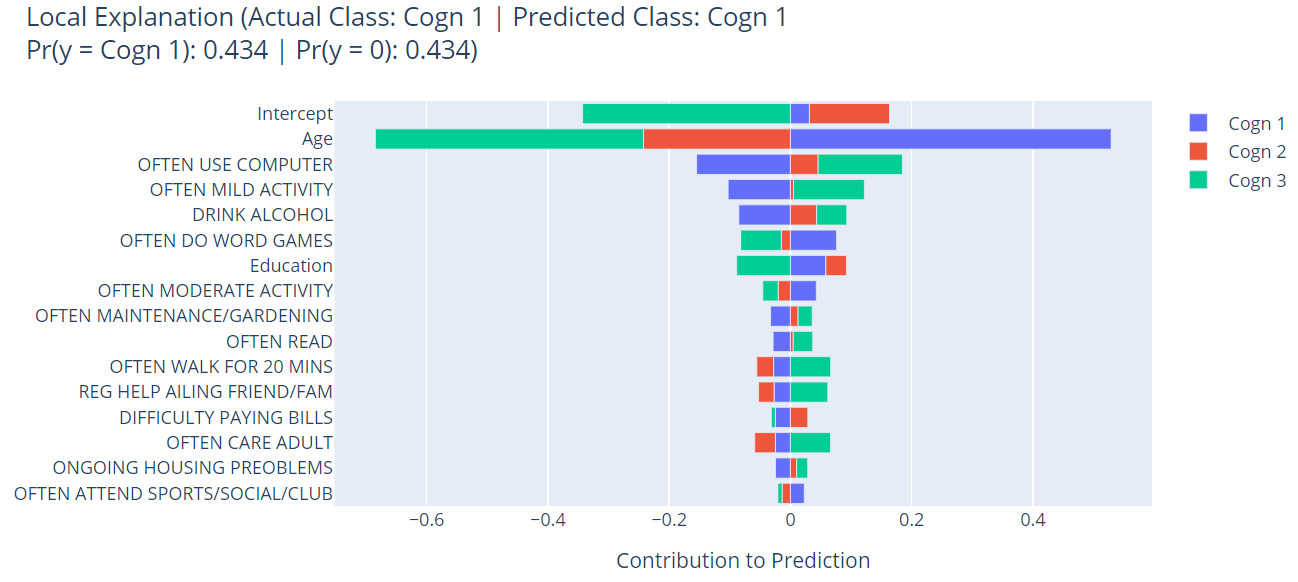


Supplementary Figure 23: Explanations obtained from the EBM model.

Table 7 - Proportion of missing data in original dataset per covariate

| **Covariate** | **% missing data** | **Covariate** | **% missing data** | **Covariate** | **% missing data** |
| --- | --- | --- | --- | --- | --- |
| Ongoing health problems | 65.5% | Often watch television | 65.0% | Age | 0% |
| Ongoing physical/emotional problem in spouse/child | 66.0% | Often do word games | 65.0% | Years of Education | 1.3% |
| Ongoing difficulties at work | 65.6% | Often play cards and games | 65.0% | Smokes | 45% |
| Ongoing financial strain | 65.7**%** | Often do writing | 65.0% | Drinks | 0% |
| Ongoing housing problem | 65.7% | Often use computer | 65.0% | Often do hobby | 65.4% |
| Ongoing problems in close relationship | 65.7% | Often do maintenance/gardening | 65.0% | Often Care Adult | 65.2% |
| Often do activities with grandchildren | 65.1% | Often bake/cook | 65.0% | Ongoing Difficulty Paying Bills | 65.4% |
| Often volunteer with youth | 65.1% | Often sew/knit | 65.3% | Often Attend Sports/Socials/Clubs | 65.0% |
| Often do charity work | 65.0% | Often walk for 20 min | 65.0% | Often Play Sports/Exercise | 65.2% |
| Often do education courses | 65.2% | Often do Vigorous Activities | 0% | Regularly Help Ailing Friends/Family | 65.7% |
| Often attend non-religious organizations | 65.0% | Often do Moderate Activities | 0% | Often read | 65.0% |
| Often pray privately | 65.1% | Often do Mild Activities | 0% | **TOTAL #** | **31646** |

*
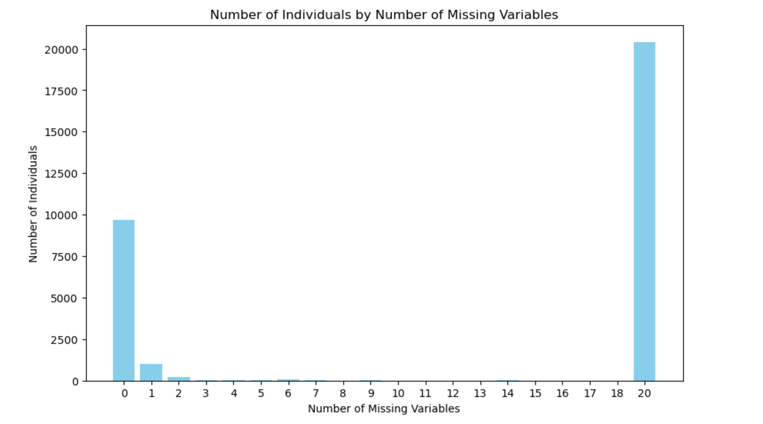
*

Figure 24 - Number of individuals by number of missing covariates
